# Origins and Molecular Features of a Chimeric Type-P4A ATPase—Guanylate Cyclase from Stramenopiles and Alveolates

**DOI:** 10.3390/ijms27156734

**Published:** 2026-07-28

**Authors:** Mark F. Wiser

**Affiliations:** Department of Tropical Medicine and Infectious Disease, Tulane University Celia Scott Weatherhead School of Public Health & Tropical Medicine, New Orleans, LA 70112, USA; wiser@tulane.edu; Tel.: +1-504-988-2507

**Keywords:** type-P4 ATPase, flippase, guanylate cyclase, alveolates, stramenopiles, chimeric protein, bifunctional protein, hybrid protein, fused genes, horizontal gene transfer

## Abstract

A chimeric protein produced from the fusion of a type-P4 ATPase, also called flippase, and a guanylate cyclase (P4GC) was originally described in ciliates and malaria parasites. An extensive search for P4GC homologs was carried out, and the protein’s structural features were determined by sequence alignments and homology modeling. P4GC is found in all alveolate lineages and in some stramenopile lineages. This suggests that the gene fusion event occurred in a common ancestor of stramenopiles and alveolates after the divergence of rhizarians, although alternative evolutionary scenarios cannot be excluded. This scenario necessitates the subsequent loss of P4GC in some stramenopile lineages. Alternatively, the gene fusion may have arisen in an early alveolate lineage and subsequently been transferred horizontally to an ancestor of oomycetes and bicosoecids, or vice versa. The sequence homology of the flippase module is not well preserved in some lineages, whereas the structure and sequence homology of the cyclase module is highly conserved in all lineages. These findings suggest that the cyclase module has been subject to stronger evolutionary constraints than the flippase module, while the latter may have undergone lineage-specific functional diversification. However, despite sequence divergence, homology modeling indicates that the predicted 3-dimensional structures of the flippase modules are highly conserved across all lineages, suggesting that structural constraints have preserved its functional role(s) within P4GC. Together, these findings indicate that P4GC has undergone lineage-specific functional diversification while maintaining a highly conserved guanylate cyclase module throughout alveolate and stramenopile evolution.

## 1. Introduction

DNA is sometimes rearranged or recombined to generate a fused gene that results in a chimeric protein [1]. In many cases, these chimeric proteins are enzymes that function in the same metabolic pathway, and this presumably facilitates the kinetics of the pathway. For example, the shikimate pathway consists of seven enzymes to produce the precursor of aromatic amino acids and other aromatic compounds. Some of these shikimate-pathway enzymes are fused in various eukaryotic lineages [2], including a supergene that has five of the enzymes [3]. Another example is the bi-functional dihydrofolate reductase-thymidylate synthase found in several eukaryotic lineages [4].

A chimeric protein without an obvious functional connection between the fusion partners is a fusion between a type-P4 ATPase and a guanylyl cyclase that was initially reported in ciliates and malaria parasites [5]. This chimeric P4-ATPase/guanylyl cyclase (P4GC) has also been reported in *Toxoplasma* [6] and other coccidia [7]. Two paralogs of P4GC are found in the malaria parasite that are designated as alpha (α) and beta (β) [8]. P4GC was also detected in oomycetes in a survey of chimeric genes but was not specifically discussed [9]. Chimeric genes appear to be more abundant in oomycetes.

**Figure 1:**
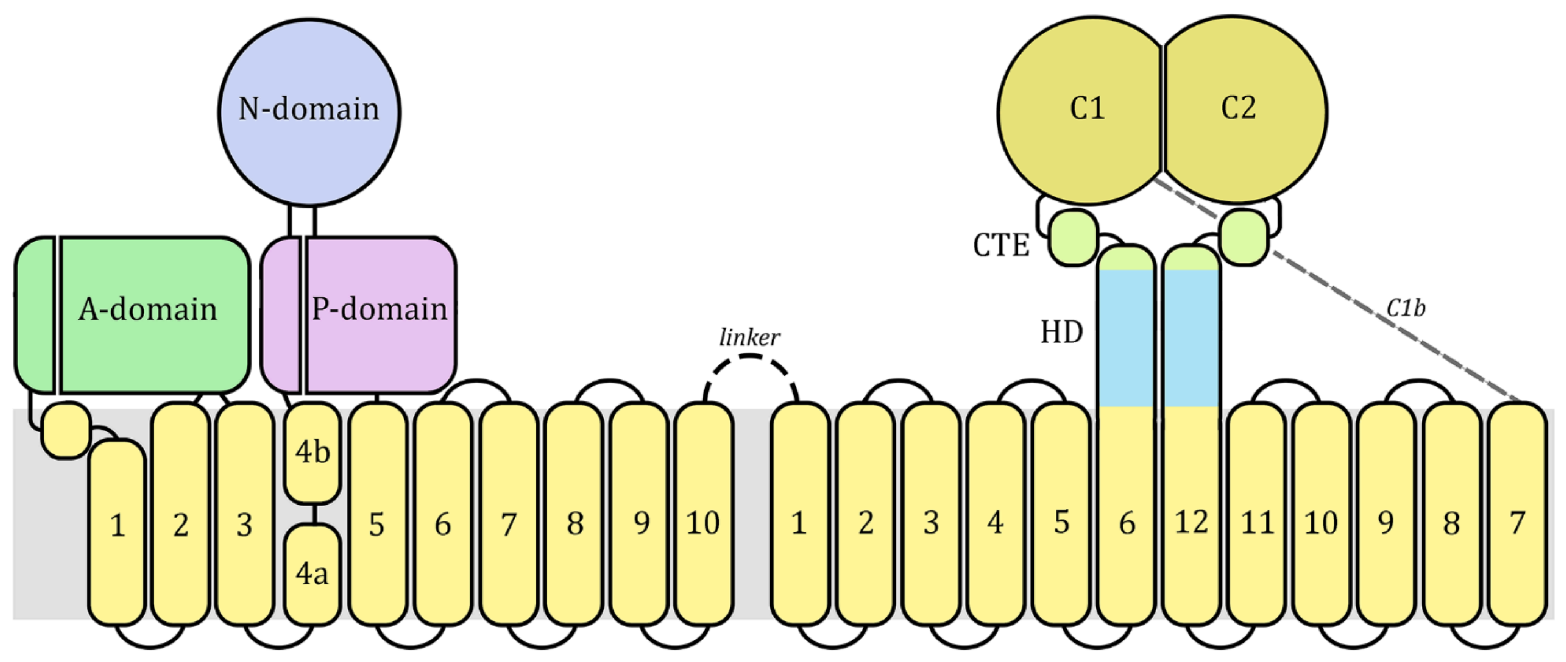
Schematic representation of P4GC with major domains labeled and transmembrane helices numbered as described in the text.

P-type ATPases comprise a large and ubiquitous gene family of membrane transport proteins that are defined by canonical structural features [10]. The first and foremost is the highly conserved phosphorylation motif DKTGT. During substrate transport, the aspartate (D) is transiently phosphorylated via ATP hydrolysis, and the protein conformation changes associated with phosphorylation–dephosphorylation cycles facilitate movement of substances across membranes. All P-type ATPases are comprised of four highly conserved domains (Figure 1) called the actuator (A) domain, the phosphorylation (P) domain, the nucleotide-binding (N) domain, and the membrane (M) domain [11]. The A-domain has phosphatase activity to remove the phosphate. The N-domain binds ATP so that the γ-phosphate is adjacent to the phosphorylated aspartate residue, and the P-domain contains the DKTGT motif. The M-domain is formed by six core transmembrane helices (cTM1–6) and a variable number of supporting transmembrane helices (sTM). A substrate-binding groove that opens to the extracytoplasmic side of the membrane (i.e., extracellular or luminal) is formed by the cTM. There is a highly conserved proline in the fourth transmembrane helix (cTM4) that forms a ‘kink,’ which is an important component of the cargo-binding groove.

There are five distinct types of P-type ATPases based on sequence, structural features, and substrate specificity [10]. The type-P4 ATPases are only found in eukaryotes and function as transporters of lipids from one layer of the lipid bilayer to the other [12]. Therefore, type-P4 ATPases are also called flippases. Flippases have four sTM helices, designated as sTM7–10, and an extended signature motif of (S/T)DKTGTLT, as well as other defining sequence motifs [13]. Three subtypes of type-P4 ATPases have been identified and are denoted with letters A–C.

Guanylyl cyclase (GC) converts GTP to cyclic GMP (cGMP), which functions in a wide range of cellular signaling pathways [14,15]. The second messenger cGMP is produced in response to a wide variety of upstream sensing events and mediates downstream events by either cGMP-dependent protein kinase (PKG) or cGMP-dependent ion-gated channels. GC is a member of the class-III cyclase family, and two forms designated as IIIa and IIIb have been described [16]. Class IIIa GC is membrane associated and class IIIb GC is soluble. The membrane form is characterized by a membrane domain with 12 transmembrane helices and is a pseudo-heterodimer composed of two cyclase modules. Each cyclase module has six transmembrane helices designated as M1–M6 and M7–M12, respectively. Similarly, two complementary catalytic domains (C1 and C2) form the catalytic domain with the substrate-binding site at the interface of C1 and C2. Between the membrane and catalytic domains is a helical domain (HD) formed by extended α-helices from M6 and M12 that interact in a coil–coiled fashion to form a stalk (Figure 1). The helical domain promotes the dimerization of cyclase-1 and cyclase-2 [17]. Between the HD and catalytic domain is a 19-amino acid region called the cyclase transducer element (CTE) that is characterized by a highly conserved leucine-proline (LP) pair that temporarily disrupts the α-helix [18]. A variable region of intrinsically disordered structure, called c1b, connects the cyclase-1 and cyclase-2 modules.

Relatively little work has been conducted on P4GC, and most of the information is available from the medically important *Plasmodium* and *Toxoplasma*. In this study, an extensive BLAST search using the tools available at the National Center for Biotechnology Information (NCBI) was carried out, and P4GC was identified in some stramenopiles and all alveolates. Stramenopiles are a diverse group that includes unicellular heterotrophs called bicosoecids, macroscopic brown algae (e.g., kelp), microscopic diatoms, fungus-like oomycetes, and the intestinal commensal *Blastocystis* [19]. They were previously called heterokonts due to the presence of two distinct flagella: a long anterior-facing flagellum with hairlike structures and a shorter posterior-facing flagellum. The stramenopiles are part of a supergroup, called SAR, that also includes the alveolates and the rhizarians [20]. The rhizarians are the more basal group, with the stramenopiles and alveolates diverging later and forming sister clades. The alveolates include ciliates, dinoflagellates, and apicomplexans [21].

Sequence alignments and homology modeling were carried out on P4GC sequences to address questions about the origin and possible function(s) of this chimeric protein. The sequence and three-dimensional structure of the cyclase module are highly conserved, and P4GC primarily functions as a GC. In some phyla, the flippase module sequence is partially conserved and missing some key motifs involved in ATPase activity. However, the three-dimensional structure is highly conserved, suggesting an important role of the flippase module in facilitating GC activity.

## 2. Results

### 2.1. Identification of Chimeric P4GC Genes

The non-redundant database and several complete genomes at NCBI were searched for *P4GC* genes as defined by a type-P4 ATPase (i.e., flippase) module and a nucleotide cyclase module within a single polypeptide. *P4GC* genes were found in some stramenopiles and all alveolates. No P4CG was detected in any other eukaryotic group. The protein accession numbers, gene ID numbers, taxonomic groups, and E-values of the conserved domains were recorded (Table S1). A total of 177 *P4GC* genes from 132 species were identified (Table 1). Sequences with major anomalies or missing large portions of the protein after an initial examination of the BLAST alignments or subsequent alignments were not subjected to more detailed analyses or three-dimensional (3D) structural modeling. This resulted in 121 relatively complete P4GC genes from 94 species.

#### 2.1.1. Stramenopiles

Stramenopile P4GC sequences are primarily represented by oomycetes, which are major plant pathogens [22], with 35 *P4GC* genes in 30 species (Table 1). Some of the genes had sequence irregularities, resulting in 19 complete genes from 18 species. Potential P4GC paralogs were identified in *Aphanomyces* with three copies in *A. invadans* and *A. stellatus*, two copies in *A. astaci*, and single copies in *A. euteiches* and *A. cochlioides*. Subsequent analyses revealed sequence irregularities in highly conserved regions, suggesting that most of these paralogs may be pseudogenes or contain errors.

An extensive search for P4GC in rhizarians and other stramenopiles was carried out, including searches of genomes that have not been fully annotated. No *P4GC* genes were detected in the genomes of the rhizarians, diatoms, or *Blastocystis*. *P4GC* genes were detected in three species of bicosoecids, which are phagotrophic flagellates of marine or freshwater environments. In two of the species, a portion of the N-terminus was missing. However, no P4GC was detected in bicosoecid *Halocafeteria seosinensis*.

A potential P4GC candidate was detected in the brown alga *Sphaerotrichia firma* (Table S1). This potential P4GC was found on an 8175 bp contig and only contained one cyclase module, and the flippase module was primarily transmembrane helices and lacked flippase sequence motifs. Furthermore, other unrelated partial genes were found on this rather small DNA contig. Doing a BLASTP search of the open reading frame corresponding to the cyclase module identified soluble GC. Thus, this contig is likely the result of aberrant contig assembly, and there is no *P4GC* orthologue in the brown algae. In addition, no class-IIIa guanylate cyclases were found in rhizarians, brown algae, diatoms, or *Blastocystis*. The soluble guanylate cyclase (i.e., class-IIIb) was detected in rhizarians, brown alga, and diatoms, and no cyclases were detected in *Blastocystis*.

#### 2.1.2. Ciliates

As previously reported in *Tetrahymena* and *Paramecium* [5,23], ciliates also have *P4GC* genes (Table 1). Several paralogous *P4GC* genes (up to 13) were detected in most ciliate species. Ciliates have high levels of duplicated genes due to massive gene duplication in ciliates [24]. Thus, iterative BLAST searches were carried out to identify a single allele from each species that exhibits the most homology to the *Blepharisma stoltei* P4GC sequence. *B. stoltei* only has one *P4GC* gene, and *Blepharisma* is the earliest branching ciliate [25]. *B. stoltei* and *Stentor coeruleus* have very high E-values (0.0) for the flippase module, whereas the other species are considerably lower (Table 1). Both *P4GC* alleles from *Ichthyophthirius multifiliis*, a well-known fish pathogen, were missing a large portion of the flippase module and substantial amounts of conserved sequence.

#### 2.1.3. Perkinsids and Dinoflagellates

Perkinsids are a sister group to the dinoflagellates and diverged close to the divergence of dinoflagellates and apicomplexans [26]. Three P4GC sequences were found in two species of perkinsids. One of the *Perkinsus chesapeaki* sequences had anomalies in the N-terminal region, and the *P. marinus* P4GC sequence had anomalies in the cyclase module. Sixteen P4GC sequences were detected in ten dinoflagellate species. Most of the *P4GC* duplications (i.e., paralogs) contained major sequence anomalies.

#### 2.1.4. Early Branching Apicomplexans

At the base of the apicomplexan branch are the colpodellids and related chromerids [27]. Colpodellids are primarily free-living predators, and chromerids are photosynthetic autotrophs. Some pathogenic colpodellids have also been described [28]. Two paralogs of the *P4GC* gene were found in the chromerid *Vitrella brassicaformis* and a single copy in *Chromera velia*. No complete genomes of *Colpodella* or other predatory colpodellid species are available.

Gregarines are primarily pathogens of invertebrates and are an early-branching clade in the apicomplexan tree [29]. P4GC was detected in two gregarine species, and in both species, two paralogs were identified. However, one of the *P4GC* genes from *Gregarina niphandrodes* lacked substantial portions of the flippases module, and the other gene lacked portions of the cyclase module. Similarly, one of the P4GC genes from *Porospora gigantea* lacked portions of the flippases module.

Cryptosporidia are closely related to gregarines [30], and single copies of the *P4GC* gene were found in all available *Cryptosporidium* species as well as in four additional species in which the genomes are not completely annotated (*C. baileyi*, *C. cuniculis*, *C. equi*, and *C. viatorum*). The sequence from an unidentified *Cryptosporidium* species isolated from cows was nearly identical to *C. parvum* and not further analyzed. Cryptosporidia had notably lower CD values for the flippases module than other phylogenetic groups (Table 1).

#### 2.1.5. Coccidia and Piroplasmids

P4GC was detected in 14 coccidian species, with 10 species having major anomalies, especially in the flippases module. Some of the *Eimeria* species exhibited rather low E-values in the flippases domain. As previously reported [7], paralogs of *P4GC* were detected in *Eimeria tenella.* However, both alleles had sequence anomalies that precluded further characterization.

*P4GC* genes were identified in 14 species of piroplasmids. The gene from *Babesia ovata* was missing some of the M-domain of the ATPase module, and the *Cytauxzoon felis* gene was missing conserved sequence within the cyclase module. In addition, a partial sequence that was missing the second cyclase domain was detected in *Cardiosporidium cionae*; a nephromycid, which is possibly a sister group to the piroplasmids [31].

#### 2.1.6. Haemosporidans

As previously reported [8], two distinct chimeric P4GCs, called alpha (α) and beta (β), have been described in malaria parasites (i.e., haemosporidans). Twenty-seven *P4GCα* and 26 *P4GCβ* genes were detected (Table 1). No P4GCβ gene was found in *P. ovale walliker*, and this is presumed to be due to an incomplete genome sequence. In addition, the P4GCβ sequences from *Haemoproteus tartakovskyi* and *P. brasilianum* were incomplete. There is a somewhat large difference in the flippase E-values between P4GCα and P4GCβ of the heamosporidans (Table 1), with P4GCα having E-values in the 10^−100^ range and P4GCβ having E-values in the 10^−50^ range.

### 2.2. Sequence Homology

The 121 complete P4GC sequences were aligned using secondary structures and highly conserved sequence motifs from flippases [13] and cyclases [32] as guides. The alignment is characterized by regions of high homology interspersed with variable regions (Figure S1). The flippase module contains more and larger variable regions than the cyclase module. The rather long linker sequence between the flippase and cyclase modules is highly variable, as is the c1b sequence between cyclase-1 and cyclase-2. The domains, sequence motifs, and highly conserved residues of the flippase and cyclase modules are readily identified in the alignments. Overall, the cyclase module exhibits more conservation than the flippase module.

Many sequence motifs are conserved in all P4GC sequences, whereas some motifs exhibit less conservation in some lineages. This variation in conserved sequence motifs is only observed in the flippase module, and the cyclase module exhibits a high level of sequence homology with known cyclase motifs (Figure S1). To quantify this homology in the flippase module, the relative conservation of these conserved signature residues was estimated as a homology score (Table 2). Stramenopiles, some ciliates, perkinsids, dinoflagellates, and colpodellids exhibit a high level of sequence homology (>0.94). Some ciliates, gregarines, *Cryptosporidium*, and P4GCβ of *Plasmodium* exhibit lower homology (<0.71), whereas P4GCα of *Plasmodium*, piroplasmids, and coccidia exhibit an intermediate level of homology. The level of homology is relatively constant among the species within a specific lineage except for ciliates. *Blepharisma* and *Stentor* exhibit a high level of sequence homology, whereas *Paramecium* and *Tetrahymena* exhibit a lower level of homology.

**Figure 2:**
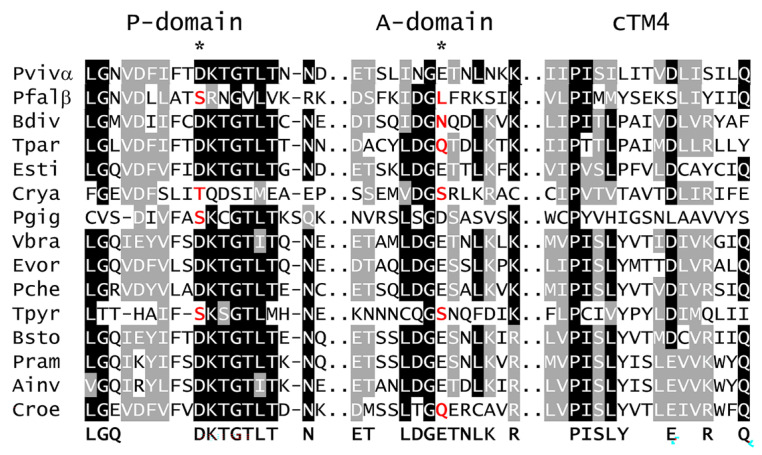
Homology of flippase sequence motifs. Shown are the alignments of three important flippase sequence motifs of P4GC representing various phylogenetic groups. Alignments are derived from the annotated alignment (Figure S1), and species are defined in Table 2. Asterisks denote the phosphorylated aspartate (D) of the DKTGTLT motif and the glutamate (E) of the LDGET motif. Deviations from these D and E residues are colored red. Below the alignments are the highly conserved signature residues. Shading indicates 70% identity (black) or 70% similarity (grey). All highly conserved residues are shown in Figure S1, and additional motifs are shown in Figure S3.

The highly conserved DKTGTLT signature motif is also altered in some of the species with low homology (Figure 2). Most notable is *Cryptosporidium*, which essentially lacks this motif. Partial conservation of the motif is seen in P4GCβ, gregarines, and *Tetrahymena*. Another important sequence motif is the LDGET of the A-domain. The glutamine (E) residue is important for the dephosphorylation of the phosphorylated aspartate (D). Loss of homology in the LDGET motif is observed in P4GCβ, piroplasmids, gregarines, *Tetrahymena*, and bicosoecids. Another potentially important sequence motif is the kink region of cTM4, which forms the cargo-binding groove. In particular, the PISLY motif is also highly conserved in type-P4A ATPases [13], as well as in most of the P4GC sequences. The greatest deviation is found in gregarines and *Tetrahymena*. Other sequence motifs and highly conserved residues are shown in Figures S1 and S2.

### 2.3. Phylogeny of P4GC

**Figure 3:**
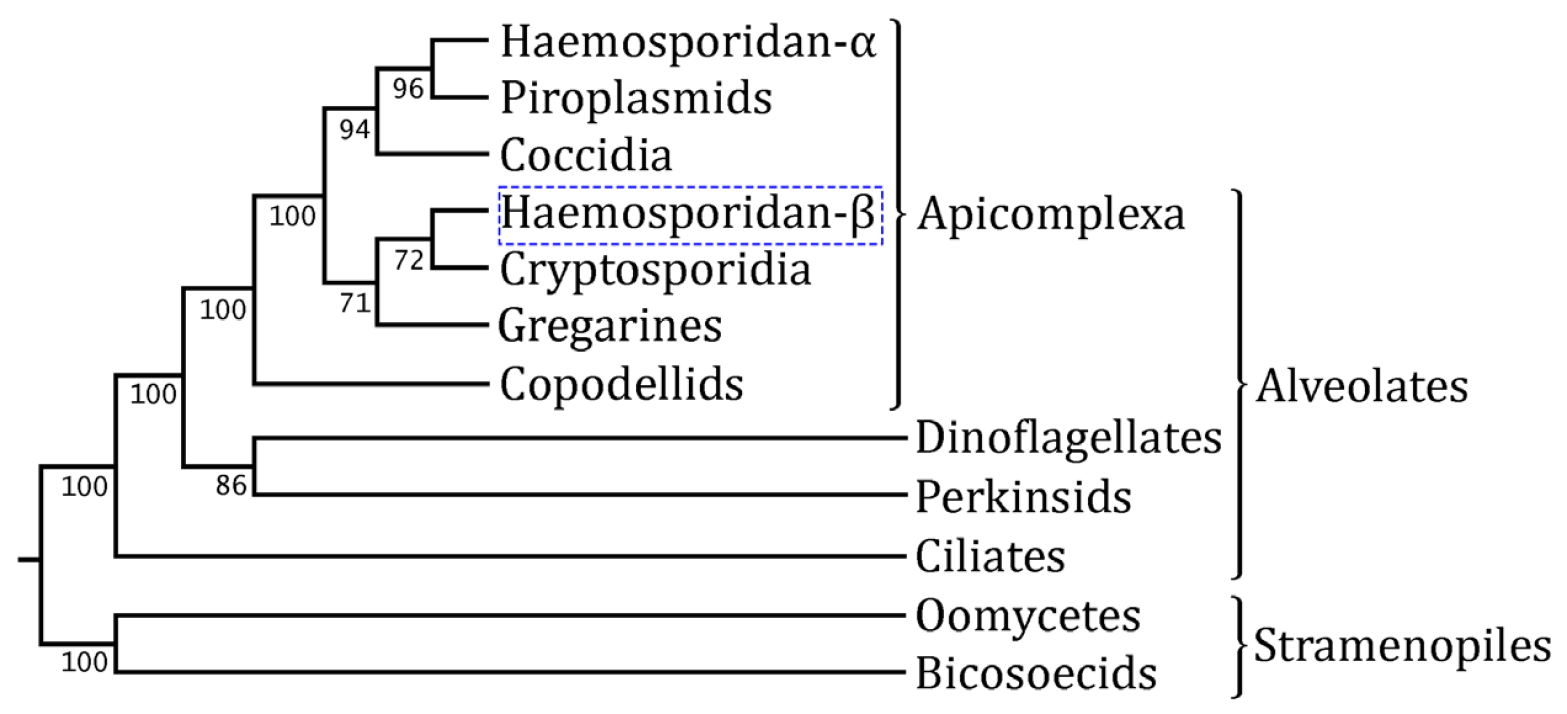
Summary cladogram of maximum likelihood phylogenetic tree. The maximum likelihood phylogenetic tree (Figure S3) was redrawn to include only major phylogenetic groups and clades. The expected branching patterns were observed except for haemosporidan-β (blue dashed box). Bootstrap values shown at the nodes are substantially lower for this clade.

A maximum likelihood tree was generated from the P4GC alignment (Figure 3). The tree topology conformed to the accepted phylogeny of the alveolates and stramenopiles [21]. The only exception was P4GCβ of the haemosporidans. P4GCβ is likely the result of a gene duplication shortly after the divergence of the haemosporidans and piroplasmids; thus, P4GCα and P4GCβ would be expected to form sister clades. Instead, P4GCβ forms a clade with cryptosporidia and gregarines. However, it is well known that following gene duplication, one gene often undergoes rapid evolution and forms a distinct paralog [33]. This likely happened in the early evolution of haemosporidans, and the two paralogs were retained and likely evolved to have different functions. In addition, the bootstrap values of this clade are low.

Other potential paralogs of P4GC were also observed in oomycetes, perkinsids, dinoflagellates, colpodellids, gregarines and *Eimeria tenella* (Table S1, Figure S3). However, these duplications were observed at the species level or perhaps the genus level in *Aphanomyces*. In addition, most of these potential paralogs were missing substantial conserved sequence and could not be aligned. These paralogs may be evolving into pseudogenes and, thus, may not be of functional significance. The paralogs of ciliates were not investigated, since ciliates exhibit massive genome duplication that complicates analysis of paralogs [24].

The P4GC subclades within the major phylogenetic groups also conformed to known phylogeny (Figure S2). Oomycetes form two major clades that are primarily represented by the genera *Phytophthora* or *Aphanomyces*. The ciliates *Tetrahymena* and *Paramecium* form one subclade and *Blepharisma* and *Stentor* form a second subclade. Symbiotic dinoflagellates form a subclade that excludes the non-symbiotic *Polarella*. Cryptosporidian subclades correspond to *C. hominis* and related mammalian and avian parasites, a distinct clade of ruminant parasites (*C. bovis*, *C. ryanae*, and *C. xiaoi*), and a clade consisting of *C. serpentis*, *C. muris*, and *C. andersoni*. The *C. serpentis* clade consists of stomach parasites, and their homology has been previously noted [34]. The tissue-cyst forming coccidia form a separate subclade from the intestinal coccidia as previously reported [35]. Piroplasmids segregate into *Theileria* and *Babesia* subclades consistent with known piroplasmid phylogeny [36]. P4GCα exhibits three major clades corresponding to avian malaria parasites, Laverania, and the mammalian parasites excluding Laverania, which is consistent with accepted phylogenies [37]. The mammalian parasites could be further separated into vivax-like, rodent parasites, *Hepatocystis*, ovale-like, and malariae-like subclades as previously reported [38]. In the case of P4GCβ, the phylogeny (Figure S3) did not conform to the accepted phylogeny of the haemosporidans.

### 2.4. Structural Homology 

Known 3D protein structures were queried by Phyre^®^ with representative P4GC sequences from the various phylogenetic groups (Table S2). The top-scoring templates that correspond to the flippase module were all type-P4A ATPases. The two most prominent templates were Dnf1 and Drs2 of *Saccharomyces cerevisiae* and the human orthologs of these proteins [12]. The Dnf1 protein (PDB Acc. No. 7drx) was chosen as the template for subsequent homology modeling studies [39]. Substantially fewer templates were identified with the cyclase modules, and generally three or fewer templates were identified per query sequence. The predominant template for the cyclase module was the mammalian class-IIIa adenylate cyclase-8 (AC8), and this template (PDB Acc. No. 8buz) was used for subsequent modeling studies [40].

#### 2.4.1. Flippase Module

The homology models of the P4GC sequences exhibit the expected P-type ATPase structure. Overall, the predicted structures are quite similar to the template and to each other (Figure S4). Even the less conserved P4GC sequences yielded 3D structures that are quite similar to the template. For example, the homology of the *Ichthyophthirius* and *Gregarina* sequences was so low that they were not included in the alignment. Nonetheless, those sequences produced homology models typical of flippase (Figure S4, Imul and Gnip panels). The most obvious difference between the homology models is the amount of intrinsically disordered loops (IDL). IDL are formed by the variable regions between conserved domains (Figure S1) and are particularly prevalent in haemosporidans, coccidia, cryptosporidia, ciliates (except *Blepharisma*), and bicosoecids. The large IDL of type-P5 ATPases from the malaria parasite and other apicomplexans has been previously discussed [38,41].

Minor differences in the M-domain were also sometimes observed due to transmembrane helices not being completely modeled. The difficulty in modeling transmembrane helices has been previously noted [42]. For example, sometimes homology modeling started after cTM2 or the last 2–3 sTM helices were not modeled. In these cases, the M-domain appears narrower, and in the case of missing sTM, the C-terminal regulatory domain is also absent (Figure S4, Imul, Pgig, Gnip, and Bdiv panels).

Considering the lower sequence homology of conserved sequence motifs in some lineages (Table 2), a detailed analysis of the A-, N-, and P-domains was also carried out with a focus on the genes with less homology.

#### 2.4.2. A-Domain

**Figure 4:**
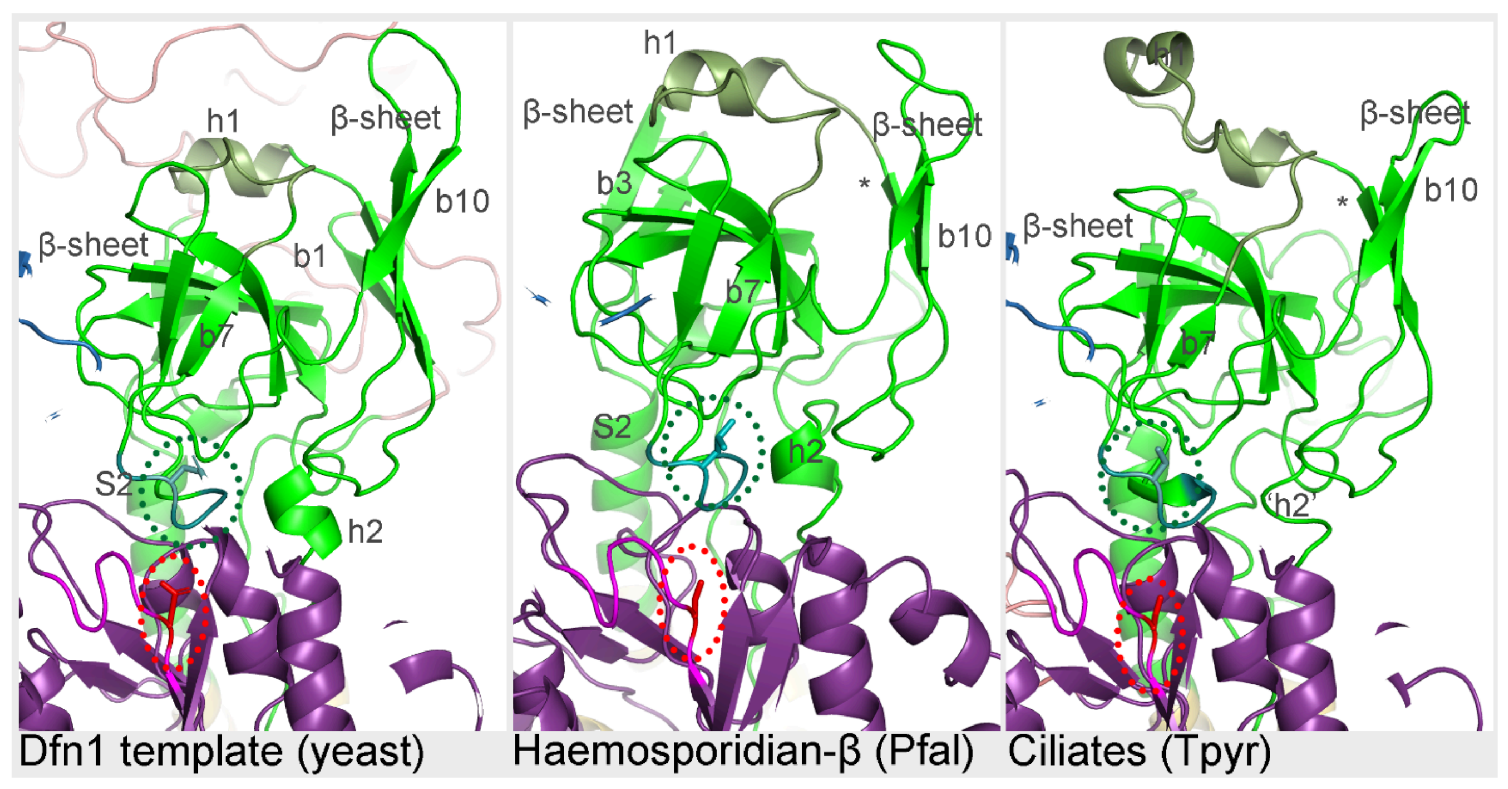
A-domain structure. Select models that demonstrate differences between species are shown and compared to the template. Some of the β-stands (b#) and helices (h#) and the stalk associated with cTM2 (S2) are labeled. The A-domain consists largely of two facing β-sheets composed of four β-strands (b1-b8-b9-b10) and seven β-strands (b3-b2-b4-b11-b5-b6-b7). The outer β-strands are labeled. The helical region between b7 and b8 is in a different shade of green with a short helix (h1). The asterisk (*) denotes positions where b1 was not modeled. The dotted teal oval highlights the conserved E of the LDGET motif (teal) and the dotted red oval highlights the conserved D (red) of the DKTGTLT motif (magenta). Note that b3 is in a different position in Pfal as compared to the template and Tpyr. See Figure S1 for positions of secondary structures in the primary sequence alignment.

The A-domain consists of a small segment from the N-terminus of the protein and the region between cTM2 and cTM3. Two anti-parallel β-sheets that approximately face each other are formed. One β-sheet is relatively flat and is composed of four β-strands, and the other β-sheet is composed of seven β-strands with some twisting (Figure 4). A helical region lies between the two β-sheets. This structure (i.e., the two β-sheets and the helical region) sits on a pedestal-like structure composed of the α-helical stalk from cTM2 and a short α-helix that connects to cTM3.

Homology scores for the A-domain are quite high, and only P4GCβ and P4GC of gregarines and *Tetrahymena* are below 0.7 (Table 2). Accordingly, the A-domains of P4GC are quite similar to the template, and there are only minor differences between the various groups (Figure 4). For example, modeling often started after N-terminal β-strand-1. Other minor differences include the region between β-strand-7 and β-strand-8 in a few of the species, a different shape of one of the β-sheets in P4GCβ, and PyMOL did not depict the second helix as a ribbon in *Tetrahymena*. The conserved E of the LDGET motif drives the dephosphorylation of the DKTGTLT motif [43], and the position of this residue is conserved and is juxtaposed with the phosphorylated D. Thus, despite the differences in sequence homology, the predicted structure of the A-domain is highly conserved.

#### 2.4.3. N-Domain

**Figure 5:**
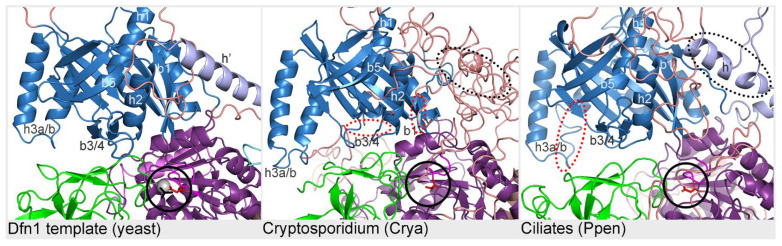
N-domain structure. Select models that demonstrate differences between species are shown and compared to the template. Some of the β-stands (b#) and helices (h#) are labeled. A twisting and curving β-sheet is formed from nine anti-parallel β-strands (b2-b1-b11-b10-b9-b8-b7-b6-b5). On the convex side of the curve, there are two helices (h3a/b and h4a/b), both with internal bends, and α-helix-1 and -2 are found on the concave side of the β-sheet. A small anti-parallel β-sheet composed of b3 and b4 is in a gap between b5 and h2 and appears to form a base. An IDL (salmon color) with a variable amount of predicted α-helical structure (designated h′ and colored light blue) is adjacent to the N-domain. The dotted black ellipses denote other manifestations of this region. B1 and b11 are the junctions with the P-domain (purple), and this junction is called the hinge region. The cleft formed by the hinge region is the ATP-binding site and is close to the DKTGTLT motif (magenta) of the P-domain (black circle). The side group of the phosphorylated residue is shown in red, and the grey sphere represents Mg^2+^. BeF_3_ is also shown in the template (black sticks). The red dotted ellipses denote the truncated b1 and missing b2 and the missing b3 and b4 in *Cryptosporidium*. Helix-3b in ciliates is not depicted as a ribbon (red-dotted oval). See Figure S1 for positions of secondary structures in the primary sequence alignment.

The N-domain is formed from a single linear sequence that starts and ends with β-strand-1 and β-strand-11. A primary feature of the N-domain is a twisting and curving β-sheet formed from nine anti-parallel β-strands. Alpha-helices flank the β-sheet on both the concave and convex sides, and a small anti-parallel β-sheet composed of two β-strands forms a base to this structure. A variable region is predicted to form a helix flanked by IDL in the template. This variable region is the most notable difference between the template and P4GC on first inspection (Figure 5). The predicted structures of this region manifest as helices that are similar to the template, fragmented helices, or random coils. This helix is set apart from the core of the N-domain and is probably not a component of the N-domain. The predicted N-domains of the various P4GC sequences are remarkably similar to the template. The only substantial difference is in the P4GC of cryptosporidia, which lacks the two-stranded β-sheet at the base of the N-domain, and cryptosporidia are missing β-strand-2 and part of β-strand-1, which form one edge of the β-sheet.

#### 2.4.4. P-Domain

**Figure 6:**
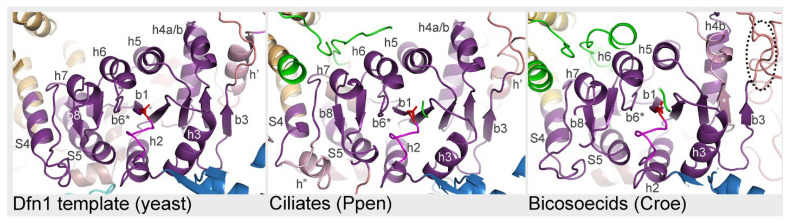
P-domain structure. Select models that demonstrate minor differences between species are shown and compared to the Dnf1 template. Some of the β-stands (b#) and helices (h#) are labeled. The primary feature of the P-domain is a twisting parallel β-sheet (b8-b7-b6*-b1-b2-b5-b4-b3) sandwiched between two rows of α-helices (h7-h6-h5-h4a and S4-S5-h2-h3). The wavy strand between b7 and b1 is not depicted as a ribbon and is designated as b6*. The P-type ATPase motif (magenta) immediately follows b1, and the side chain of the phosphorylated residue is shown in red. B4 and h4a of the bicosoecid P4GC are derived from a variable region and shown in a lighter shade and marked with asterisks. Two variable helices designated as h′ and h″ and colored in a lighter shade are sometimes observed. The black dotted oval highlights the lack of helix′ and its replacement with IDL. See Figure S1 for positions of secondary structures in the primary sequence alignment.

The P-domain consists of a small segment between cTM4 and the N-domain and a larger segment between the N-domain and cTM5. In addition, a bent α-helix located between cTM6 and sTM7 appears to interact with a short helix in the first P-domain segment to form a collar around the stalks of cTM4 and cTM5. This collar may also interact with the regulatory domain. The core structure of the P-domain is a modified Rossmann fold [11] formed from a twisting parallel β-sheet sandwiched between two rows of α-helices. Like the A- and N-domains, the P-domains of P4GC are quite similar to each other and to the template. One difference is a variable region that exhibits a range of manifestations, including helices, fragmented helices, or IDL (Figure 6). In addition, a short α-helix after the stalk of cTM4 is occasionally observed in a region that usually forms an IDL. In P4GC from bicosoecids, β-strand-4 and helix-4a exhibit the expected structure but are derived from the sequence of a large variable region between β-strand-3 and β-strand-4.

#### 2.4.5. Cargo-Binding Groove

Another key element of flippase is the cargo-binding groove formed by the cTM. Conserved polar residues on cTM2, cTM4, and cTM6 stabilize the polar headgroup of the lipid cargo near the entry site on the outer leaflet [44]. A hydrophobic ‘gate’ is found near the exit site of the inner leaflet that is formed primarily by eight conserved nonpolar residues. The modeled P4GC structures form a cargo groove that is quite similar to the experimentally determined groove of flippase (Figure S5a). The only exception is P4GC from the gregarine *Porospora gigantea*. In the other species, there are some substitutions of polar residues with non-polar residues or vice versa in the entry site and hydrophobic gate, respectively. In addition, there is also a high level of sequence conservation in the key residues making up the cargo-binding groove (Figure S5b). In particular, the positions of polar and non-polar residues are conserved. These rows of polar residues may provide tracks that form salt bridges with the polar headgroup of the lipid as it moves through the groove [44]. The high level of sequence and structural conservation implies that the flippase module may be capable of binding and flipping lipids.

#### 2.4.6. Cyclase Module

Guanylate cyclase is a pseudo-heterodimer with two cyclase modules. Each module consists of a transmembrane domain composed of six membrane-spanning helices, a helical domain, a highly conserved CTE, and the catalytic domain. Between cyclase-1 and cyclase-2 is an IDL often designated c1b. The two helical domains form a coiled-coil stalk with the globular catalytic domain on top and the CTE between the stalk and catalytic domain. Each monomer of the catalytic domain has a curved anti-parallel β-sheet surrounded by α-helices on the convex side. Two catalytic domains dimerize to form a wreath-like structure when viewed from the top, with the α-helices on the outside and the concave portions of the β-sheets forming the substrate-binding site (i.e., active site).

**Figure 7:**
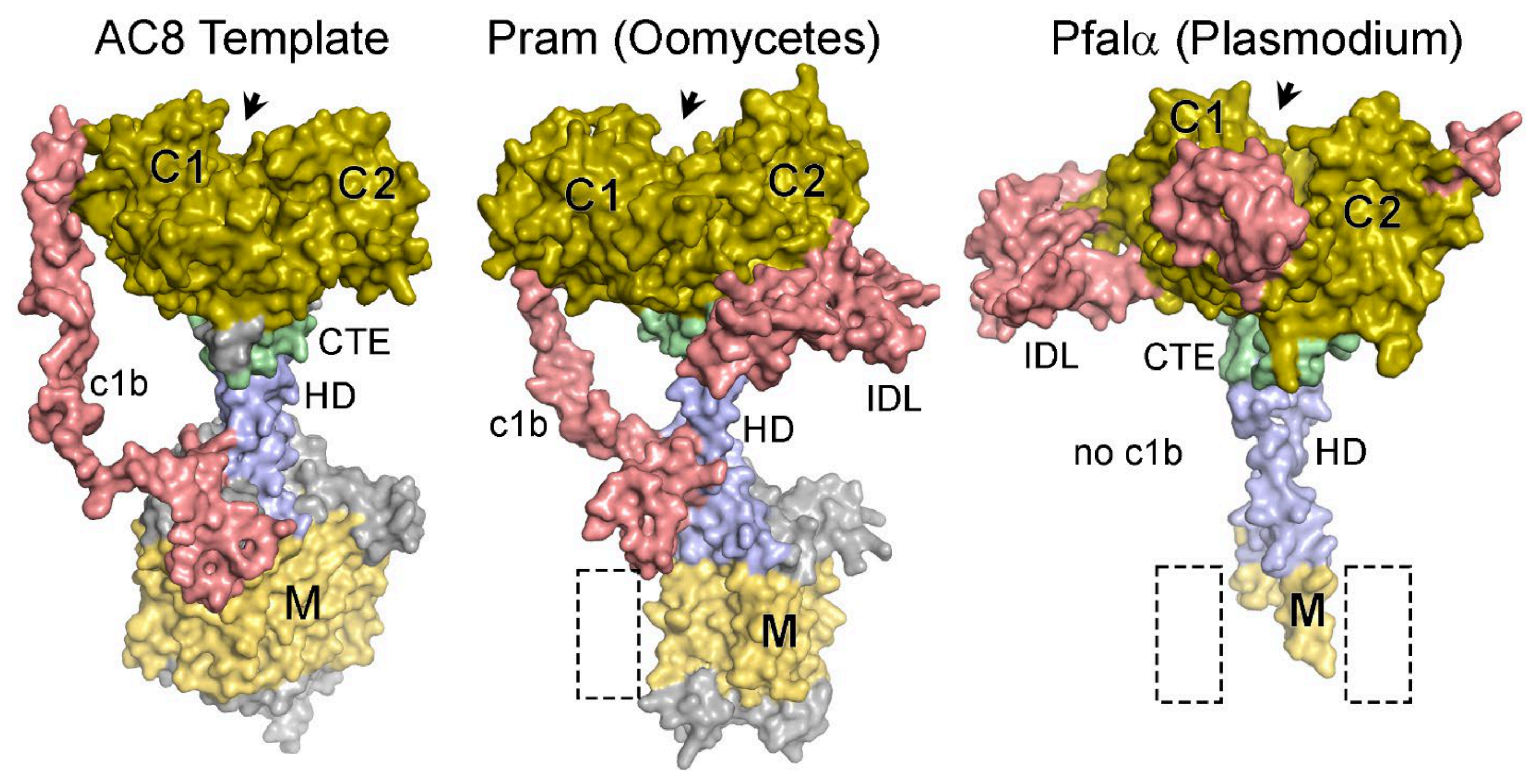
Homology models of the cyclase modules. Modeling of P4GC with the AC8 template produced structures as a single polypeptide with cyclase-1 and cyclase-2 connected by c1b (e.g., Pram) or the two cyclase modules were modeled as separate polypeptides (e.g., Pfalα) that could be dimerized in PyMOL. Membrane domains (M, yellow), helical domains (HD, light blue), cyclase transducer elements (CTE, light green), and catalytic domains (C1 and C2, gold) are denoted with abbreviations and colors. The c1b connector and other intrinsically disordered loops (IDL) are colored salmon. Arrowheads denote the substrate binding grooves. Single-polypeptide models (e.g., Pram) generally lacked transmembrane helices M1–M5, as indicated by the dashed box. Modeling of separate polypeptides (e.g., Pfalα) generally started in M6 and M12 for the cyclase-1 and cyclase-2 modules, respectively, and the missing transmembrane helices M1–M5 and M7–M11 are indicated by two dashed boxes.

Homology modeling of representative P4GC proteins using AC8 from *Bos taurus*, a class-IIIa cyclase, as a template generally recapitulated the known structure of cyclases. Models were either a single polypeptide or two polypeptides corresponding to the cyclase-1 and cyclase-2 modules, respectively (Figure 7). In a few of the single-polypeptide models, the dimer was not formed. The most notable discrepancy in P4GC models was in the M-domain, especially in cyclase-1, where the transmembrane helices were not modeled for many of the P4GC sequences. The difficulty in modeling transmembrane helices is well known [42]. In the models where cyclase-1 and cyclase-2 were modeled as separate polypeptides, a dimer could be formed in PyMOL most of the time. IDLs were present in many of the P4GC proteins. However, the IDLs were not as numerous nor as large as the IDLs of the flippase modules. These differences between the cyclase-module models are not attributed to specific lineages. The secondary structures making up the catalytic domain are highly conserved and are nearly identical to the template in all P4GC (Figure S6).

## 3. Discussion

Overall, the analyses indicate that P4GC originated early during SAR evolution and was retained under a relatively strong evolutionary constraint. While the guanylate cyclase module is highly conserved in both sequence and structure, the flippase module exhibits greater sequence divergence despite retaining a conserved structure. These contrasting evolutionary patterns suggest differential selective pressures acting on the two modules and provide a novel paradigm for the functional evolution of chimeric proteins. For example, one might surmise that the two modules initially functioned independently as flippase and guanylate cyclase—especially considering the long stretch of flexible sequence between the two modules. Subsequently, the flippase module could have evolved to support the cyclase module, and the specifics of this evolution could be different in the various lineages. Likewise, there could have been a functional repurposing of P4GC in the various lineages.

**Figure 8:**
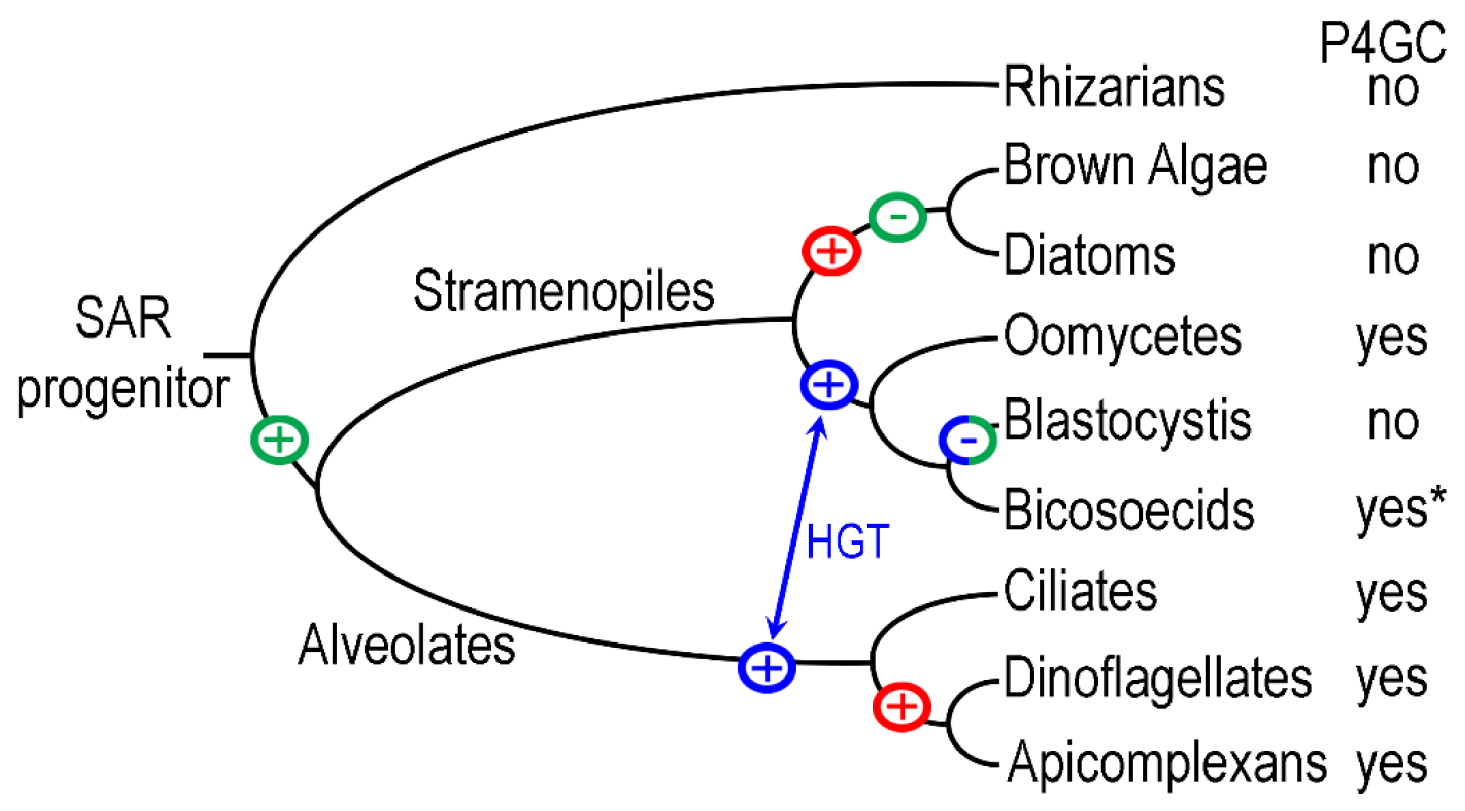
Possible origins of P4GC. The tree depicts the currently accepted phylogeny of alveolates [21] and stramenopiles [19]. The presence of P4GC is indicated on the right. The asterisk (*) indicates that P4GC was found in three of four bicosoecids with sequenced genomes. P4GC could have originated in a common ancestor of stramenopiles and alveolates (green oval with +) and was subsequently lost in photosynthetic stramenopiles (green oval with −). Alternatively, P4GC could have originated in either stramenopiles or alveolates and transferred horizontally (HGT) to the other (blue oval with +). All three scenarios require a loss of P4GC in *Blastocystis* (two-tone circle with −). The red ovals depict the gain (+) of plastids (i.e., photosynthesis).

A thorough search revealed that P4GC exists in all alveolates and some stramenopiles. No evidence of P4GC in other phyla, including rhizarians, was detected. These findings are consistent with a gene fusion event occurring in a common ancestor of the stramenopiles and alveolates after the divergence from rhizarians, although horizontal gene transfer is a plausible alternative explanation (Figure 8). Origin of P4GC in a common ancestor of stramenopiles and alveolates would necessitate the subsequent loss of P4GC from photosynthetic stramenopiles and *Blastocystis*. Alternatively, P4GC could have arisen in an early alveolate and was subsequently transferred horizontally to a common ancestor of the oomycetes and bicosoecids, or vice versa. Since bicosoecids and *Blastocystis* are believed to share a common ancestor [19], all three scenarios require P4GC gene loss in *Blastocystis*. Notably, *Blastocystis* has no cyclase genes and has lost numerous other genes possibly due to its commensal lifestyle [45].

There is also a question about the origin of the membrane-associated GC (i.e., class IIIa) in SAR. The only class III GC found in SAR is P4GC. Only the soluble GC (i.e., class IIIb) is found in rhizarians, diatoms, and brown alga, all of which lack P4GC. Thus, it appears that the acquisition of a membrane form of GC and the gene fusion are contemporaneous events. The high level of conservation of sequence and structure in the cyclase module indicates that P4GC functions as a guanylate cyclase. Indeed, *Paramecium* P4GC has been demonstrated to have guanylate cyclase activity with minimal adenylate cyclase activity [5,23], as well as both P4GCα and P4GCβ in *Plasmodium* [8]. In contrast, neither ATPase nor phospholipid flippase activity has been experimentally demonstrated for P4GC.

### 3.1. The Flippase Module Is a Necessary Component of P4GC

The sequence conservation of the flippase module, as determined by the homology score, ranges from somewhat low (55–71%) in *Tetrahymena*, *Paramecium*, gregarines, cryptosporidia, and P4GCβ of haemosporidans to very high (>96%) in oomycetes, *Blepharisma*, dinoflagellates, and colpodellids. In species with low homology, the highly conserved sequence motifs are affected (Figure S3). Most notable are the aspartate in the signature DKTGT motif and the glutamate of the LDGET motif. Both these residues are necessary for the ATPase activity. This raises the possibility that ATPase activity has been modified or lost in some lineages, although experimental validation is required. The flippase module may therefore be under weaker evolutionary constraint than the cyclase module. Since all eukaryotes have several flippase genes [13], there may be less evolutionary pressure to maintain the flippase function of P4GC.

However, many of the flippase sequence motifs are highly conserved in all P4GC proteins (Figure S3), and several studies have demonstrated that the flippase module is essential. For example, expressing the flippase and cyclase modules of P4GCβ as separate proteins had a severe impact on malaria parasite biology [46]. Similarly, mutation of the aspartate (D) residue in the signature DKTGT motif of P4GCα to an asparagine (N) led to non-viable parasites in *Plasmodium* [47], and substitution of the aspartate (D) for glutamate (E) led to non-viable parasites in *Toxoplasma* [48]. Thus, it is possible that in some lineages, P4GC has ATPase or flippase activity. Alternatively, as often suggested, the flippase module may play a supporting role or facilitate GC activity and not require ATPase or flippase activity [6,46,47,49]. For example, the flippase module may be needed to ensure correct localization or orientation in the membrane. In that regard, many flippases have an accessory subunit (CDC50), and this subunit is needed for the correct localization of P4GC to the plasma membrane [46,50]. Another protein that potentially plays a chaperone-like role in the targeting and function of P4GC is the unique GC organizer (UGO) initially identified in *Toxoplasma* but found in all apicomplexans [48].

Homology modeling indicates that the structure of the flippase module is highly conserved, even in the species with lower sequence homology. All the predicted structures of P4GC are quite similar to each other and similar to the known structure of flippase. This includes a high level of structural conservation in the ATP and lipid-binding sites. Only minor structural deviations were observed, suggesting that the overall structure has remained evolutionarily conserved. These results imply that there is evolutionary pressure to maintain the flippase structure even with the loss of sequence homology associated with ATPase activity. This preservation of flippase structure suggests that the flippase module is integral to the function of P4GC. The high level of structural conservation and partial sequence conservation suggests that the flippase module may primarily contribute to regulation, membrane localization, or structural coupling of the cyclase domain rather than phospholipid transport.

### 3.2. Possible Role of P4GC in Apicomplexa

All the studies addressing the function of P4GC have been carried out in the medically important *Plasmodium* and *Toxoplasma*. The inability to disrupt *Plasmodium* P4GCα [49,51,52] and the *Toxoplasma* orthologue [6,53] indicates that the P4GC gene is essential. In contrast, it is possible to disrupt P4GCβ and recover viable blood-stage parasites [51,54,55]. Accordingly, P4GCα and P4GCβ are expressed in different stages during the malaria parasite life cycle. P4GCα is expressed in asexual blood stages and gametocytes and appears to be expressed at higher levels in schizonts and merozoites [46,47] and is localized to vesicle-like structures of unknown origin [47,52]. No specific function has been assigned to P4GCα other than to suggest an involvement in parasite egress from the infected host cell [47]. *Toxoplasma* P4GC may also be involved in parasite egress [6], as well as host cell invasion [56].

P4GCβ is expressed in gametocytes, zygotes, and ookinetes and is essential for the completion of the life cycle through the mosquito [51,55]. Disruption of the P4GCβ gene had no significant effect on gametogenesis [54] nor on the subsequent development to ookinetes [46]. Specifically, disruption of P4GCβ disrupts microneme secretion, gliding motility, and invasion of the mosquito midgut [46]. The P4GC of *Toxoplasma* is also involved in microneme secretion and gliding motility [6,56]. Interestingly, in both *Plasmodium* [46] and *Toxoplasma* [56] the development of the invasive motile forms (i.e., ookinetes and tachyzoites, respectively) involves the movement of P4GC from intracellular vesicle-like structures to a plasma membrane location at the apical end. This is possibly correlated with the activation of microneme secretion at the apical end. Microneme secretion exposes adhesins that are critical for gliding motility and cell invasion [57]. The regulation of microneme secretion has been previously associated with cGMP or cGMP-dependent protein kinase [58,59]. Parasite flippase, independent of P4GC, has also been associated with microneme secretion [60]. Since gliding motility involves pulling protein complexes through the lipid bilayer, a possible explanation is that membrane lipid asymmetry facilitates efficient gliding motility.

The duplication of P4GC in haemosporidans led to paralogs specializing in either the blood stage or the mosquito stage of the infection. Since *Toxoplasma* has no stage analogous to the ookinete stage and does not exhibit vector transmission, a single P4GC gene may be sufficient. Piroplasmids, though, are also vector transmitted by ticks and have a stage analogous to the ookinete, called the kinete [61]. This implies that the orthologue of P4GCα in piroplasmids functions in both the blood stage and vector stage of the life cycle, and a P4GC paralog is not necessarily needed for vector transmission. It should also be noted, even though the relevance is questionable, that the biology of ookinetes and kinetes is a little different. Ookinetes of haemosporidans are formed in the vector gut and cross the intestinal epithelium and form an oocyst stage beneath the basal lamina of the vector gut. In piroplasmids, the kinetes are formed intracellularly within gut epithelial cells and eventually migrate to and invade the salivary glands and form sporoblasts. Both oocysts and sporoblasts undergo sporogony to produce sporozoites.

There are also questions about the upstream sensing events associated with cGMP and GC as well as the downstream effector events. Regarding *Plasmodium*, the downstream events only involve PKG since there is no evidence of cyclic nucleotide-gated ion channels [49]. Several potential substrates of PKG have been identified, but no specific mechanisms of action have been elucidated [62]. Potential upstream activators of GC and the subsequent microneme secretion include a decrease in extracellular potassium [63], phosphoinositol [64], parasitophorous vacuole acidification [65], and phosphatidic acid [48]. In regard to the latter, it has been suggested that the flippase module senses phosphatidic acid, thereby regulating guanylate cyclase activation. The highly conserved structure of the cargo-binding groove (Figure S5) suggests that lipid binding is possible.

### 3.3. Possible Roles in Other Phyla

Although the data does strongly suggest that P4GC plays an important role in activation of microneme secretion and motility, micronemes and gliding motility are restricted to apicomplexans. This means that the role(s) of P4GC in stramenopiles, ciliates, and dinoflagellates is likely different from that in apicomplexans. Furthermore, the role of P4GC in earlier diverging apicomplexans, such as colpodellids, gregarines, and cryptosporidia, could be different from that in later diverging apicomplexans, such as coccidians, piroplasmids, and haemosporidans. The early diverging apicomplexans tend to be free-living or extracellular, whereas the later diverging apicomplexans are obligate intracellular parasites that depend on microneme secretion not only for motility, but also for host cell invasion [29].

The localization of P4GC to the ciliary membrane in ciliates [5] suggests a possible role in cilia function. Additionally, cGMP does regulate cilia beating in ciliates as well as metazoa [66]. In marine vertebrates, such as starfish, a flagellar receptor GC plays a key role in sperm chemotaxis by activating flagellar beating [67]. Somewhat related, *Blastocystis* has no genes associated with flagellar activity and is also lacking cyclase genes [45]. However, the microtubule-based motility of cilia and flagella is radically different than the actin-myosin-based gliding motility.

In addition, except for *Blastocystis*, which has no cyclase genes, there is a dichotomy between membrane GC (i.e., class-IIIa) and soluble GC (i.e., class-IIIb) within SAR. In the species that have P4GC (alveolates, oomycetes, and bicosoecids), there is no gene for the soluble GC. In contrast, species without P4GC (rhizarians, brown alga, and diatoms) only have a gene for soluble GC. This may have some implications about the role(s) of guanylate cyclases in stramenopiles.

### 3.4. Limitations

A major limitation in this study, as well as in many other studies, is the large imbalance in available sequence data and determined 3D structures to serve as templates for homology modeling. Most data are derived from humans, other metazoa, and model organisms. There is also a fair amount of data available from agriculturally important plants and animals and from a smattering of eukaryotic pathogens. For example, the vast majority of the work on P4GC is from *Plasmodium* and *Toxoplasma*. Similarly, potential flippase and cyclase templates for homology modeling were primarily from animals, especially humans, and yeast. Because P-type ATPases and class III guanylate cyclases adopt highly conserved folds, the available templates are expected to provide reliable models of the overall domain architecture. Nevertheless, lineage-specific structural differences cannot be excluded. Similarly, biochemical data is limited to *Plasmodium* and *Toxoplasma*, and there is a lack of experimental data across multiple lineages. Therefore, the functional inferences herein are based on this limited biochemical data from important human pathogens and the conservation of the sequence and structure across all lineages. Stramenopiles, ciliates, and dinoflagellates are extremely large and diverse phyla, and relatively few sequences are available. In contrast, there are a lot of sequences from pathogens, such as malaria parasites, piroplasmids, and *Cryptosporidium*. There were also numerous sequences available from oomycetes, which are important plant pathogens [22]. 

Despite this imbalance in the available sequences, the results are probably not substantially affected, since the phylogenetic trees conformed to the known phylogenies. Nonetheless, more sequences from stramenopiles may help in resolving the question about the origin of P4GC and potential horizontal gene transfer events. Similarly, there are only four available genomes from the bicosoecids, which makes it difficult to determine the significance of P4GC being found in three of the four species.

## 4. Materials and Methods

### 4.1. BLAST Searches

The previously characterized P4GC sequence from *Plasmodium falciparum* (Gene ID PF11_0395), ciliates (Acc. Nos. AJ238859 and AJ238858), and *Toxoplasma gondii* (Gene ID TgGT1_254370) were used as queries in the initial BLASTP searches of non-redundant protein sequences at NCBI [68]. Genes that had a P-type ATPase and a cyclase combined in a single protein as determined by conserved-domain (CD) analysis [69] available at NCBI were recorded, including the E-values of the best-scoring CD along with the protein accession and gene ID numbers (Table S1). The type-P4 ATPase CDD accession numbers include cI21460, cd02073, and tigr01652 and cyclase CDD accession numbers include cI11967, pfam00211, smart00044, cd07302, and COG2114.

If sequences contained major deletions or irregularities in the alignments associated with either the BLAST searches or subsequent alignments, then a notation was made, and those sequences were not included in the alignments. The newly identified complete sequences were used as queries in secondary BLASTP searches against the same or related taxonomic groups to ensure that no P4GC sequences were missed. In cases where complete sequences were available from multiple strains of a single species, the reference strain for that species was chosen unless there were anomalies in that sequence. A few sequences were altered to correct errors, such as inappropriate introns or start codons.

Several genomes were also searched using TBLASTN (Table 3) with a query from the same or closely related phylogenetic group using tools available at NCBI. If necessary, reading frames and exons were determined using the Open Reading Frame program at NCBI. Supplemental Table S1 contains a description of all the sequences identified and analyzed in this study, and a supplemental file of the sequences in FASTA format is also included in Supplementary Materials.

### 4.2. Sequence Alignments

Sequences were aligned with ClustalW within MEGA12 [78]. Initially, the alignments were carried out within phylogenetic groups corresponding to (1) stramenopiles, (2) ciliates, (3) perkinsids plus dinoflagellates, (4) colpodellids plus gregarines, (5) cryptosporidia, (6) coccidia, (7) piroplasmids, (8) haemosporidans α-type, and (9) haemosporidans β-type. These alignments were then manually adjusted using conserved sequence motifs and structural features, such as transmembrane helices and secondary structures as determined by Swiss Model (see below), as guides. In cases where the transmembrane domains were not well modeled, CCTOP [79] was used to predict transmembrane helices and assist in the alignment. This process of manually adjusting the alignments according to conserved structural features substantially increased the number of aligned identical and similar residues.

The phylogenetic group-specific alignments were then merged into a single alignment and readjusted, resulting in 121 complete P4GC sequences. This alignment is available in Supplementary Materials (P4GC_Align.fas). Figure S1 is an annotated alignment with representatives from the phylogenetic groups. Annotations include the domains, consensus sequence, conserved motifs, and positions of secondary structures. The standard single-letter amino acid code was used for the consensus sequence as well as symbols for similar amino acids [80]. These include Φ for hydrophobic sidechains (VILMFYW), ψ for large hydrophobic sidechains (VILM), Ω for aromatic sidechains (FYW), π for small sidechains (GAS), z for polar sidechains (NQSTKRHDE), with plus (+) designating positively charged sidechains (KRH) and minus (−) designating negatively charged sidechains (DE).

Previously described highly conserved residues and sequence motifs of flippases [13] and cyclases [32] are included in Figure S1. A sequence homology score was developed for the flippase module to approximate the degree of homology between the various phylogenetic groups. For each of the highly conserved residues, one point was given for an exact match or conservative replacement (R/K/H, E/D, S/T, I/L/V/M, Y/F, or G/A), a half point for similar residues (i.e., either polar or nonpolar), and zero points for no conservation. The average for all conserved residues within a domain was calculated and then the average was calculated from the domain scores to produce an overall score. A homology score of 1.0 represents 100% homology, and the score approximates the percent sequence homology.

### 4.3. Homology Modeling

Representatives from the various phylogenetic groups and subclades were then used as queries in a Phyre^®^ search [81]. The predominant template for the type-P4A ATPase domain was Dnf1 [39] from *Saccharomyces cerevisiae* (PDB Acc. No. 7drx) and the predominant template for the cyclase domain was adenylate cyclase-8 (AC8) [40] from *Bos taurus* (PDB Acc. No. 8buz). Homology modeling was carried out using Swiss Model [82] available at https://swissmodel.expasy.org/ with Dnf1 (PDB Acc. No. 7drx) and AC8 (PDB Acc. No. 8buz) as templates.

Previous modeling of type-P5 ATPases indicated that Swiss Model was superior to AlphaFold [41]. This was due to a high level of hallucination associated with IDL and AlphaFold was strongly biased towards modeling the cargo-binding site after cation-transporting P-type ATPases.

Images were generated with PyMOL 3.1.6.1 (Schrödinger LLC, New York City, NY, USA). The various domains were demarcated in different colors, and the color names refer to the names given by PyMOL. The color scheme was as follows: M-domains in both flippase and cyclase (yellow orange); cTM4 kink (orange); A-domain (green); loop and helix between the two b-sheets of the A-domain (smudge); N-domain (sky blue); variable helix in N-domain (light blue); P-domain (violet purple); signature P-type ATPase motif (magenta) with phosphorylated residue in red and showing side chain; helices forming collar at base of the P-domain (dirty violet); variable helix in P-domain (violet); regulatory domain (aqua marine); cyclase helical domain (light blue); CTE (pale green); cyclase catalytic domain (olive); intrinsically disordered loops (IDL) or variable regions (salmon); and secondary structures from variable regions (light magenta).

### 4.4. Phylogenetic Analysis

Variable regions were removed from the alignment described above and phylogeny determined by the Maximum Likelihood method and the Jones-Taylor-Thornton (JTT) model plus Freg [83]. Preliminary analysis determined that the JTT model was the optimal model. Bootstrap values were calculated from 500 replicates. Analyses were carried out and trees were constructed with MEGA12 [78].

## 5. Conclusions

P4GC is more widespread than previously thought and is found in dinoflagellates as well as some stramenopiles. The protein likely functions as a guanylate cyclase, and there are some questions about the functionality of the flippase module. The flippase module, whether it has flippase/ATPase activity or not, has a highly conserved structure and is likely an integral part of the protein that probably plays a supportive role to the cyclase module or facilitates GC activity. In *Plasmodium* and *Toxoplasma* P4GC may play a role in triggering microneme release and gliding motility. Such a role would be restricted to the Apicomplexa, and thus, P4GC likely plays other roles in other lineages.

## Figures and Tables

**Table 1:** Number of P4GC sequences detected and analyzed.

	CD E-Values ^a^		Number of Sequences (and Species) ^b^	
Group (# Subclades) ^c^	Flippase	Cyclase	Identified	Complete
Bicosoecids (n.a.)	e-97–e-132	e-26–e-56	3 (3 spp.)	1 (1sp.)
Oomycetes (2)	0.0	e-32–e-56	35 (30 spp.)	19 (18 spp.)
Ciliates (2)	e-11–0.0	e-49–e-52	13 (13 spp.) ^d^	8 (8 spp.)
Perkinsids (n.a.)	0.0	e-29–e-47	3 (2 spp.)	1 (1 sp.)
Dinoflagellates (2)	0.0	e-29–e-49	16 (10 spp.)	8 (7 spp.)
Colpodellids (n.a.)	0.0	e-30–e-47	3 (2 spp.)	3 (2 spp.)
Gregarines (n.a.)	e-8–0.0	e-22–e-44	4 (2 spp.)	1 (1 sp.)
Cryptosporidia (3)	e-25–e-34	e-30–e-36	14 (14 spp.)	13 (13 spp.)
Coccidians (2)	e-16–e-119	e-27–e-42	15 (14 spp.)	4 (4 spp.)
Nephromycids (n.a.)	e-96	e-15	1 (1 sp.)	0
Piroplasmids (2)	e-90–e-144	e-32–e-47	14 (14 spp.)	12 (12 spp.)
Haemosporida-α (3)	e-83–e-105	e-30–e-48	27 (27 spp.)	27 (27 spp.)
Haemosporida-β (x)	e-34–e-54	e-31–e-41	26 (26 spp.) ^e^	24 (24 spp.) ^e^
Total	-	-	177 (132 spp.)	121 (94 spp.)

^a^ Range of expected (E)-values from Conserved Domain analysis with e-XX representing 10^-XX^ and 0.0 approaching infinity for the flippase and cyclase modules (Table S1). ^b^ Number of genes (and species) initially identified in BLAST searches and number of complete genes with no major sequence abnormalities. ^c^ Major taxonomic groups and number (#) of subclades within that group. Not applicable (n.a.) indicates too few representatives to determine subclades, and the x indicates the subclades did not conform to well-accepted phylogeny. ^d^ Numerous paralogs were identified in the ciliate species, and only a single gene from each species was chosen. ^e^ These were not added to the species total.

**Table 2:** Homology of P4GC with conserved flippase residues.

	Domain				
Species (Group)	M	A (E)	N	P (D)	AVG
Pfalα (haemosporidan)	0.85	0.77	0.76	0.84	0.80
Pvivα (haemosporidan)	0.91	0.95	0.82	0.80	0.87
Pfalβ (haemosporidan)	0.59	0.66 (L)	0.58	0.60 (S)	0.61
Bdiv (piroplasmid)	0.70	0.84 (N)	0.69	0.85	0.77
Tpar (piroplasmid)	0.57	0.80 (Q)	0.71	0.75	0.71
Esti (coccidian)	0.85	0.96	0.84	0.86	0.88
Crya (cryptosporidian)	0.39	0.73 (S)	0.56	0.52 (T)	0.55
Pgig (gregarine)	0.96	0.59 (D)	0.66	0.65 (S)	0.71
Vbra (colpodellid)	0.96	0.96	1.00	0.99	0.98
Evor (dinoflagellate)	0.96	0.93	0.98	0.96	0.96
Pche (perkinsid)	1.00	0.86	0.95	0.96	0.94
Ppen (ciliate)	0.57	0.75 (N)	0.68	0.58	0.64
Tpyr (ciliate)	0.52	0.66 (S)	0.50	0.53 (S)	0.55
Bsto (ciliate)	0.93	0.98	0.97	0.98	0.97
Pram (oomycete)	1.00	0.95	0.92	0.98	0.96
Ainv (oomycete)	1.00	0.95	0.95	0.96	0.97
Croe (bicosocecid)	1.00	0.79 (Q)	0.92	0.95	0.91

Homology scores were calculated as described in the Materials and Methods and represent the amount of conservation, with 1.0 being 100% conserved. Scores for the M-, A-, N-, and P-domains of P4GC and the average (AVG) of those scores are shown. Parentheses in the A-domain indicate substitutions for the conserved E of the LDGET motif. Parentheses in the P-domain indicate substitutions for the conserved D of the DKTGTLT phosphorylation motif. Species abbreviations are defined in Supplemental Table S1.

**Table 3:** Genomes searched with P4GC as a query using TBLASTN.

Accession Number	Species	Group	Ref
GCA_000512085.1	*Reticulomyxa filose*	Rhizaria	[70]
GCA_036867785.1	*Plasmodiophora brassicae*	Rhizaria	[71]
GCA_008330645.1	*Cafeteria roenbergensis*	Stramenopile (bicosoecid)	
GCA_945788115.1	*Cafeteria burkhardae*	Stramenopile (bicosoecid)	
GCA_965644315.1	*Caecitellus pseudoparvulus*	Stramenopile (bicosoecid)	
GCA_001687465.1	*Halocafeteria seosinensis*	Stramenopile (bicosoecid)	
GCA_000151665.1	*Blastocystis hominis*	Stramenopile (commensal)	[72]
GCA_047495695.1	*Thalassiosira rotula*	Stramenopile (diatom)	[73]
GCA_000310025.1	*Ectocarpus siliculosus*	Stramenopile (brown algae)	[74]
GCA_018398765.1	*Chromera velia*	Colpodellid	[75]
GCA_000585135.1	*Chromera velia*	Colpodellid	
GCA_001593455.1	*Cryptosporidum baileyi*	Crytosporidia	[76]
GCA_004337835.1	*Cryptosporidum cuniculis*	Crytosporidia	
GCA_029747635.1	*Cryptosporidum equi*	Crytosporidia	
GCA_014529505.1	*Cryptosporidum felis*	Crytosporidia	
GCA_004337795.1	*Cryptosporidum viatorum*	Crytosporidia	
GCA_051169045.1	*Cryptosporidum serpenti*	Crytosporidia	[34]
GCA_020283715.1	*Cytauxzoon felis*	Piroplasmid	[77]

Shown are the NCBI Accession Numbers of the genomes, species, taxonomic groups, and references if published.

## Data Availability

Contact the author to receive data generated by this study. The alignment in fasta format and a text file of the P4GC sequences are included in the Supplementary Materials.

## Supplementary Materials

The following supporting information can be downloaded at https://www.mdpi.com/article/10.3390/ijms27156734/s1.

## Abbreviations

The following abbreviations are used in this manuscript:

AC8	Adenylate Cyclase-8
Acc. No.	Accession number
A-domain	Actuator domain of P-type ATPase
BLAST	Basic Local Alignment Search Tool
C1/2	Catalytic domain (cyclase)
CDC50	Cell division cycle-50 (accessory protein of type-P4 ATPase)
CD	Conserved Domain
CDD	Conserved Domain Database
CTE	Cyclase Transducing Element (cyclase)
cTM	Core transmembrane helix (flippase)
GC	Guanylate Cyclase
HD	Helical Domain (cyclase)
IDL	Intrinsically Disordered Loops
M-domain	Membrane domain (flippases and cyclase)
NCBI	National Center for Biotechnology Information
N-domain	Nucleotide binding domain of P-type ATPases
P4GC	Type-P4 ATPase and guanylate cyclase chimeric protein
PDB	Protein Database
PKG	Cyclic GMP dependent protein kinase
SAR	Stramenopile-Alveolate-Rhizaria supergroup
sTM	Supporting transmembrane helix (flippase)
UGO	Unique GC organizer
