# Supplementary material for: Origins and Molecular Features of a Chimeric Type-P4A ATPase—Guanylate Cyclase from Stramenopiles and Alveolates
Source: Int J Mol Sci. 2026 Jul 28;27(15):6734. doi: 10.3390/ijms27156734 (PMC13466520; doi:10.3390/ijms27156734)
Supplement: Supplementary file 1 [file ijms-27-06734-s001.zip › Supplemental Figure S1.pdf]

Supplemental Figure S1. Annotated alignment of select P4GC sequences. Sequences were aligned with ClustalW within MEGA-12 as described in the Materials and Methods. Shown are representatives from the phylogenetic groups corresponding to: haemosporidans α-type (PvivA), haemosporidans β-type (PfalB), piroplasmids (Tpar, Bdiv), coccidia (Esti), cryptosporidia (Crya), gregarines (Pgig), colpodellids (Vbra), dinoflagellates (Evor), perkinsids (Pche), ciliates (Bsto, Tpyr), oomycetes (Ainv, Pram), and bicosocecids (Croe).The domains and transmembrane helices are denoted above the alignments. The domains of the flippase module are membrane (M), actuator (A), nucleotide binding (N), and phosphorylation (P). The transmembrane helices are designated as core (cTM1-6) and supporting (sTM7-10). Helices extending beyond the lipid bilayer and forming parts of the A- or P-domains are designated as stalks (S) and maintain the number of the cTM. Following sTM10 there is a small region corresponding to the regulatory (reg) domain of flippases and this is followed by a long linker connecting the flippase and cyclase modules. The guanylate cyclase module consists of two cyclase modules separated by intrinsically disordered structure called c1b. The cyclase modules form a dimer with a membrane domain consisting of 12 transmembrane helices (M1-12), a helical domain (HD1-2), a cyclase transducer element (CTE1-2), and a catalytic domain (C1-2). The first line below the alignment shows a consensus sequence in which 70% of the residues are identical (black shading) or similar (gray shading) using the standard single-letter amino acid code or the following symbols for similar amino acids: Φ for hydrophobic sidechains (VILMFYW), ψ for large hydrophobic sidechains (VILM), Ω for aromatic sidechains (FYW), π for small sidechains (GASP), ζ for polar sidechains (NQSTKRHDE), plus (+) for positively charged sidechains (KRH), and minus (-) for negatively charged sidechains (DE).The next line below the alignment are highly conserved (cons) residues and motifs of flippases and cyclases. The next line denotes the positions of the secondary (2o) structural elements with α-helices designated with h, β-stands designated with b, and turns designated with t. These secondary structural elements are numbered in the order as they appear in the flippase domains as designated by the capital letter after the number. Helices with bends are denoted as a or b. The asterisk denoted secondary elements that are likely present, but that were not depicted as such in PyMOL.

```
PvivA -------------------------------------------------------------------------------------------------------MMERKKMHEKANDGYKAKYSGKLFANHANMKNQNVSENKQPNDFTTAEAAWMEKKQKLYKD
PfalB ------------------------------------------------------------------------------------------------------------------------------------------------MKTQTLSLMNINGKRK--FL
Bdiv  ----------------------------------------------------------------------------------------------------------MTSKAVTRKQSKIGPSSQSPPATKVGETSSATTCDEKQQVTSTNKDELITKRHNLCFG
Tpar  -----------------------------------------------------------------------------------------------MDDSSIKRFKSHNVSYSFPESKSDSRETLNSYKKLLISDKRSSYRDDHTGLNDTRKRAEPDKAHSLYLG
Esti  MADPPGRPLPPLAGSSNRAPSGRYSGSGPPPPSRPPPSAAAAAHQQQPALRSEGNGRRGAKRNKVHSSKYKVRKHGASTLASAAHLEGQDEEHHYPLESGAEEEGTSRRHRRRGTKQHHGDSEGDMGATRKHPSKTIIGKLGMTAAGSAGRTALMEQKRKLYKD
Crya  ------------------------------------------------------------------------------------------------------------------------------------------------MLDHATETNTLYGEDESIKT
Pgig  --------------------------------------------------------------------------------------------------------------------------------------------------------------------
Vbra  -------------------------------------------MDGGSAVSPEAIHVMPTATHSNHHLPPQQSQGQWAEHITSGTHPQGSLSSMWWQNIMKRVPLRVGSAHNNAAPTRDLSDIPSGGRAGSQRASVASVGMSVASDPAHERRLSNLSNVGKLFR
Evor  ------------------------------------------------------------------------------------------------------------------------------------------------------MKPEEATETPTAAT
Pche  --------MSTSPDDHSPFAGVFPDQSLARSGSEPSRARRLQHRATAGSFTPQVAAVNERLRPRLQTQSTRGLARAKEIAREVIPNFLRGRSSSSSQVTPFDFSEDDFPSDFSDIANGDYSLRNSIQPSSSSSNESIQLSFRPYVSDAPAEFHVQNNRTLSELT
Tpyr  -----------------------------------------------------------------------------------------------------------------------------MASRATIQEQIFKQFQESKKRKEKEEKDEESTRTYSRKI
Bsto  ------------------------------------------------------------------------------------------------------------------------------------------------------------MGEDKLFK
Pram  -----------------------------------------------------------------------------MALSLHPLAFRSGISSFPAPTSEVSLASAKVDATSPADAVTATHTKKSHGKPGWLARVFRRNRVTDFPRQAGFCDDSDDSEDLIKSR
Ainv  ----------------------------------------------------------------------------------------------------------------------------MAVAPLDATKSTESLRQAESANASFPGRRMQTSATTSESG
Croe  --------------------------------------------------------------------------------------------------------------------------------------------------------------------

              |->     A-domain          <-||->          cTM1         <-|            |->       cTM2       <-||  S2 |->  A-domain
PvivA TNNQPLWDFRKIQIN-PTDTDELISFPTNQICSNKYGTYSFLFKSLYEQFLRLPNIWFLIICLLEFMPAFQNLSNYM-YYTKHSSIYLLAFFVCISIVKNVYEDSRRSNI---DSQINNRLCHVV----------------------DGSNSQLKAVRWMDLTV
PfalB GTNNKIY--RKVIIN-PTSEDDIQKFCRNYFRIYNFSLYNFIRRLISFDAILVYSLFLTVYIFSE-INHGET-KK---YLFIDTAISLFFNIILLIVIESLFELKKLKDV---KNANSQYYLRIV----------------------PKMSYFEKVM-TKDIKV
Bdiv  TKLQPEWESRSLKIN-PVHSDDIQRFVGNEQHLHKWTLARFVLRIVGINIRRLPYIWGILLFVINLVTHSLLLRKGSPKLPWYSNGAVIFTALFISLLTNALGSVKRYFF---GRCLDFKKYHIL----------------------QARKARLNTVRSCDLVV
Tpar  TKIQPAWETRTLKIN-PVTSDDLSRFLGNEHHTQRWSIISQIMFILLYNLRRLPFLWSIFIFLCEVIA-PFILPSGVPRRAWYSSAIPLFVSLFMSIFVNVLEFTKRYNL---GRFLDNKKCVLL------------------------KDGKLSSVRSGDVAV
Esti  TTQQPLWGIRKLTIN-PIDKDDLRRFPENAVHTTKYTFLSFLPKALWKQFHRLPNWWLV-AVLILEFIPLPRFHAL------SPWSTFLPLAFTLGV-CLVK-ELLLDLQRRATDRWRNFRVCTII----------------------DGRRPQLRLVHWESLRV
Crya  IRKKPVWISRKIVLDISGKNKNENNYSKNEYSHSKYNYLQFIIVTFWGKCKKIPIIIGL-ISFIISLFMNIE-GK------YKWLFLAPSLFFSIFPPFIV-DLYIYTKMRIADYKVNNKHCFVF----------------------ESNKMEFILTKWKYIKV
Pgig  ------------------------------MTTSLYSTTTFIPMNLWHQLRRLPNLWLMGVLVV-RMIVFFLGQYNE-RLGNILLSFLPVFLTVVSTAIC--DQFFDSRRRQFDAVLNQRYVSVV---------------------KPGKNRSLKLVHQSRLNV
Vbra  GTEGEDGTRLIRFYASEGARGGGEDLPDNTVRTSRYTVLNMVPKNLFEQFKRAANFWFLWISIL-QLLPQELSPT------NKWATIMPLSVVIFI-TMCK-DAIEDHRRALNDRSVNNFPVQKI----------------------DPYLKESKIVKWKHLKV
Evor  PASSRFQEGRRRIAFG-PNVRKQQIFSSNYVSTTRYKLWTIVPLNLFEQFHRVANIWFLIVSTL-QMLPFKISPT------TSWATLVPLCGMLFV-TFCK-DAYEDLKRWRDDCRVNQQTCKVFS--------------------GNDGRDNFDTVKWSEVKV
Pche  ASDPDATVAEELGLFC-LTSAQHRTDFHNRISSTKYHLWSFVPVNLWEQFHRVANVWFLLVGIC-QMLPFDLSPT------SKWATIAPLVLVLGV-TMAK-DGIEDYRRYRNDTKVNKRLCRVVV---------------KATDDDGQDIGGLELMPWENVTA
Tpyr  ELMHGELINKQQILQEKICQTNKITYYKDD-KYIVFRFLLGLFKHYFYRFGNSATIIIIFILLL-TPVYNMLAERLSFNQEDYLYIYIGQLAFGLVAILIR-DLYIFQTHQGLLSQLNQSQYTLFQKFSDDKILQIVRNRQDENGEISQKVQTYTSAEVSSIQI
Bsto  TSSAPGSDFRQISIDRGRASELIVFPTNYI-TTAKYTILTLLPKNLFEQFHRIANIWFLIVSIL-QLL-PLDLSP------TSNWATIAPLSLVLTVTLFK-DVYLDWCRHKMDRKINNKIVNIW----------------------DENQGKFIEIKWKDLEV
Pram  LLFVNDAQRTMEYAAYMQKHHRSDPYWTNTVRSSKYTALNFLPKSLFEQFRRVANFYFLIISLL-QL--CTDLSP------TNEYSTIGPLMLVLTATMIK-EGLEDRGRHQQDWIVNHGKTEALNGTLEG----------KKKEVAEARLGDFQTIFWKNLRV
Ainv  SPPGRVLFVNDRQATRAAMAKLGLSYAGNVVRTSKYSVLSFLPKNLMEQFRRVANFYFLIISLL-QL--ATPYSP------TNQYSTVGPLVLVLVATMIK-EAVEDKVRHDADKTVNHGKTLVFVE----------------------ALGGYREMLWQELQV
Croe  MNLVEAEPSEVWIGPAAAGRSAAQGTTPNAISTAKYSPWLFIPQCLVEQFYRTANVYFLAIAVV-QI--VTDLSP------TNQYSTLVPLLAVILLSMAK-EGVEDWRRHSADSTSNKAPVRVLRA------------------------GGWAVAERQEIRP
70%            ζ   Φ           F  N   T +Y            ζF R    Ω        ζψ                                   -          D  ψN     ψ                                ψ  ζζψ V
cons                                N     KY    F P     QF    N  F                                                       D   N                                        W
2o             bbbbb                                                                                                          bbbbb                            bbbbbb    b
                b1A                                                                                                            b2A                               b3A
```

```
                                                  A-domain
PvivA GSIIRLIEN-EQVPADILLLSCNNN-EGLVYIETSLINGETNLNKKCCVNE-TR------NETSIYGI-CNIRGRIICEKPNSNME-------SFNGSLKLDA-------------------------------------------------------------
PfalB GNIIRIFQG-DEFPADVVILYVKNN-ANAI-VDSFKIDGLFRKSIKYAVDK-YK------IDKDYLKMLSEINGVIRCELPNKNIF-------CFQGNFKLDK-------------------------------------------------------------
Bdiv  GNILKLTCD-EDVPADMILLACSNP-DGQVHIDTSQIDGNQDLKVKFCAKD-TR------LEASIHSF-ANIRGQVTCDRPSSDTE-------SFNGTIRLRG-------------------------------------------------------------
Tpar  GNILKLSCD-EQVPADCVVLYTSNP-DGALYLDACYLDGQTDLKTKYSIND-FK------IESSIRSY-NNLKGNIICDKPCPDMN-------KFSGLLKIKG-------------------------------------------------------------
Esti  GNIVRLTDD-EEVPADVVVLATSNP-EGIAYVETSKLDGETTLKFKQGVKE-TR------TETYPLAIA-GIRGRVVCEKPTSSMD-------QFTGSLKLDA-------------------------------------------------------------
Crya  GDLICILDG-EQFPADVVILHSS---GKMAYISSEMVDGSRLKRACEPTCSQTD------FERAVKDII-LTPGKITCDVPSDDIY-------HFEGSLKWEG-------------------------------------------------------------
Pgig  GEIIRLSRM-EEVPADMILFSTSMPSGVAKI-NVRSLSGDSASVSKQAVRK-TR------DVIQLNLVS-KIKGLLTFESSVSDVNTMKTSNHSFTGRLHLKG-------------------------------------------------------------
Vbra  GDIVVLESE-QDVPADIVVLATSG-DEGECYVETAMLDGETNLKLKHAVEL-TK------NDNQIRLIA-ALRGFIKCELPNKRLD-------SFSGTIGKDE-------------------------------------------------------------
Evor  GNFVKLFRD-QPVPADMLLVYSTT--GSVAYVDTAQLDGESSLKPKHSADE-TQ------HMIRNASLR-SVEGHMEADLPNELVG-------RFDGCLYLKG-------------------------------------------------------------
Pche  GSIVYLSRG-EPVPADILLVASSNS-DGVVYVETSQLDGESALKVKQALPEARR------MFRSLPLVSECV-GSMTCDAPNGFIN-------EFNGLFRLDG-------------------------------------------------------------
Tpyr  GDIILLKKG-DLCPADCLILDMKES---FCSAKNNNCQGSNQFDIKRP-PQVTQILSSSKIKGYHFEYRKILNGRIEFVSSSENQY-------SFEGYVKLNK-------------------------------------------------------------
Bsto  GNVVLLTEENCQVPADIMLLTSSHQ-EKLVYIETSSLDGESNLKIRNCVYEIASIFENESPDSAIKKLYHLDEGVLRTEQPNNRLH-------AFDGSLKLKG-------------------------------------------------------------
Pram  GHVVKLRDQ-EQVPADIVLLFSSQE-NGEAMCETSSLDGESNLKVRHCIKW------NQITPQTPRDFADTVHGEIRCEAPNKRLY-------SFDGVMQISNATTQAAGEPASRQGRT---------------------------------------------
Ainv  GDVVKVLDG-ESFPADLVILETSFM-DGQAQVETANLDGETDLKIRTRPDW-------KVDLTSVGMFPSMVTAEIRCEAPNRRLY-------TFDGVVKMKQ-------------------------------------------------------------
Croe  GDIVEVRTD-EQFPADLVILATSAP-DGRAMVDMSSLTGQERCAVRSAVHL------TQGLSNDASRLAK-LSAGVVMVAPSADLG-------SVHGQIHVWPDGTMPPKSIDLSTAVDVPWSTVTTATGPVSMEPGKWTTEATPGAAPAGPVSGPASAAASGW
70%   Gζψψζψ ζζ EζφPADψψψL   S         ψETζζLDGζζζ ζφKζ                                    Φ   ψζGζψζCD P ζζψ        FζG ζKψ  ^->
cons  GD           PAD                ET  LDGETNLK R
2o    bbbbb          bbbbb        bbbbb          bbbbb                    hhhhhhh  bbbbbbb                bbbbb
       b4A            b5A          b6A            b7A                       h1A      b8A                   b9A

                                                    A-domain                                 |->     cTM3      <-|                                      |->   cTM4
PvivA --------------------------------HPRATSLSINNVVFKGSYIKNTDYIFGVIIYTGTDTKIMKNILKNVYKSGNV---NRELNIYTILTIIFTLICVFISVLCKWTEDDKFKNGTH-------------FLLITVKDNLFESIVKYTLLYANIIPIS
PfalB --------------------------------HPRSLLLNYENFALQSSVLKGAEYIDAVVVYTGADTKKNLNIPQKIEENKTF---CIKMNNIVYYLIFMYFVFVVLSIVIKTIFFHKKNSFQN------------------SRDSFLSMLEDFVGLYILVLPIM
Bdiv  --------------------------------HPRARNVHIDNFVMKGSIIRNTGAVYGVITHTGPDTKVAQNIGRGKHTCK-LGSMEGIINHFTVVIACIYLLCLFVSTGTRWSVLLVSYRR-K-------------RGTATVIESLLFVMARFILIYGGLIPIT
Tpar  --------------------------------YPRPCNITVDNFIMKGSILRNTSYVYAVVVYTGPDTKASQNFLRILKPIK-IGCLEQTINGISIFLGLIYVVCLTTSIIVRYVTVLRGVGKFS-------------KNVQSPVNSLLFIIFRFTVLYSPIIPTT
Esti  --------------------------------HPRATSLDLSNFIHRGSHVRNTEWVYGVVVYTGHDTRSL---RSARHPSLKFAHIESEVNSYTLVSFFIIALLILISVMSKSSVQ-DRERVNNVRTEFPSSVSFILGSTEALQNPWLAILRYMALFLAVIPVS
Crya  --------------------------------KPRAISLNKDNFVQMFSKLTFANWVLAVVVYAGKDTRISCK-KHFYENKSKYYPRMKFENTLSLFLTFVITVFIIILFILIFINNESKFDNYSNYFTGS-----NGNYNNIYKSNWKIMIEFILLFNMCIPVT
Pgig  --------------------------------HPRPSNLSTNNFIPAGSVLVSPDWIYGVVVHTRCNRPCERRGRGSDRLFTSN---HFRSRENLAVLVLFLLATIVSSTLLLGCWEAWSVARIKENSPTLL---GRLLYDNEMDIVLTATYTLFLLWSLWCPYV
Vbra  --------------------------------FPRVEPIEQKNFVLKGSKIRNTKWLYGAVVYTGSDTRMMQNSKAAPSKRSRI---ERQTNYLLAIIFSVLLTACI-VSVILALSFPLGE---KVETFFA-------GRQMDYADKIWMIVTFIILYNNMVPIS
Evor  --------------------------------YPRG---GLKNMLLRGSTIRNTSYVIAFVVYTGADTKVVRNSSRSLTSKRSQV--EVTSNKLLTIVFLMLFIISI-ASALARAWLLADATGSKNLSWA----WPLTGGDDLQDNPYMAILTFLIGYNNLIPIS
Pche  --------------------------------GLR-EPASAQNLVLRGSTVSNIKWCCGVVVYVGRDTKIAMNMTANPPMKRSRV--EQQINSYLVFVCGLMFTLSLALMMAFACTSETREEFESFV-----------GLDGDKVSLGRQFLTFLILFNNMIPIS
Tpyr  --------------------------------DPNGENINDQNMIFRGQELMYTEWCVALVLYVGYDCRYFQIYRYPKEKIYFPK--GSINNKIEQYFFLTYLFIIIFTLVSSIIDFTQPF-------------NKEFNSDDADQQTGQVSATYFLEYMIFLPCI
Bsto  --------------------------------HPRAIPIDINQMVLRGSSIRNTRWVLGLVVFSGSDSKLMRNSKTPPHK-RSNM--ERKVNKYLAVVFSLLFLMVFISTSISLSYYYSNKT------------AVEYFSGTTVDNQPLIIFTFLILYNSLVPIS
Pram  ---------------------------HGVASVSEEIPITIENVLLRGMKLCNTKWVVGVVVGGGNDTKLVQNMKAIPSK-FSRL--DRIANRCIFLIFSVLFGVCCFSSVQASLFAVKVHEKPGY------ARAFPFIGKFHPAYFLEAWVTYLILYNNMVPIS
Ainv  -----------------------------DDRPVWEVPLTISNVVLRGMKLTHTAYVEGVVVASGAETKLIQNTKQSPSK-FSRL--DVIANRCILVVFSVLFFVCCVSTAWSTWRSTVYNNRLSSKVH-TAFGTTTNWSYLWNQDILSTFITFLILYNNLVPIS
Croe  VAERAAAGDSPEPTQVLVGGDDVTAARFGLDGHVVEAVGTKRFAPRGSELRVTSFVVGVAVFTGPSTKMALCSGRARSK-RGRM--DAEVNQSIWLIFTLLFLITAGSVAFHSNWQRQNSGAM--------QYLTFLGETRGATDVSRAFVTILILYNNLVPIS
70%                                   PR   ψζ ζNφψφ+GS ψ NTζΩψζπVVVY G DTK                       N                                                       <-^   F   LY ζ  PIS
cons                                               RG   RNT     G     G   K                       N                                                                     PIS
2o                                    bbbbbbb                bbbbbbb     hhhhh
                                        b10A                   b11A        h2A

      cTM4        <-| S4  |-> P-domain
PvivA ILITVDLISILQSVLIENDNRISTFENELSEPSIIDEGDEHGDLRITKSCNFFRKYTFFLNNFSRRNFTKNRFSSTNTEQNSDDRKSFISTFDKLRAFKSYFNNKSSLINSKSHSNTAHNRSSLTENDSLQGKRANRKNHLLARVKNAFFRKDRLVNFEANMRD
PfalB MYSEKSLIYIIQSLRIENDLRMR-----------------------------------------------------------------------------------------------------------------------------------------------
Bdiv  LPAIVDLVRYAFSTNFDGHMDYSFLNDDSASKDQDFPD------------------------------------------------------------------------------------------------------------------------------
Tpar  LPAIMDLLRLLYSPFYNTYSD-SIIKNLN---------------------------------------------------------------------------------------------------------------------------------------
Esti  LPFVLDCAYCIQAWLIQGDAAMTVGAPQQPLGSAFVFGLGGSHGDPCALAGQPRSKAGELASAAAGGGETFNESTGGETSAGGQKEKPASADATTSNNGAAERDMEAE----------------------------------------------------------
Crya  VTAVTDLIRIFEFIKLSFNKSILNDINY----------------------------------------------------------------------------------------------------------------------------------------
Pgig  HIGSNLAAVVYSLTLEADPTLKNL--------------------------------------------------------------------------------------------------------------------------------------------
Vbra  LYVTIDIVKGIQAILIERDPNMFY--------------------------------------------------------------------------------------------------------------------------------------------
Evor  LYMTTDLVRALQAFIMES--------------------------------------------------------------------------------------------------------------------------------------------------
Pche  LYVTVDIVRSIQAYLTQR--------------------------------------------------------------------------------------------------------------------------------------------------
Tpyr  VYPYLDIMQLIIVYKSRKIFDNPFQFLSEQDGRFQEDDEGSNE-------------------------------------------------------------------------------------------------------------------------
Bsto  LYVTMDCVRIIQAQFIRW--------------------------------------------------------------------------------------------------------------------------------------------------
Pram  LYISLEVVKWYQARRIEN--------------------------------------------------------------------------------------------------------------------------------------------------
Ainv  LYISLEVVKWYQAKKIES--------------------------------------------------------------------------------------------------------------------------------------------------
Croe  LYVTLEIVRWFQARRIEG--------------------------------------------------------------------------------------------------------------------------------------------------
70%   L    D  +  Q
cons  LY   E  R  Q    D
```

```
                                                                                                                                                                                      P-domain       <-||->   N-domain
PvivA KHREPFHGHAKSGVENGGKIGVVNGGKIDVENRGDVNGEANCQASSHADKPTPLNTQVAKRRGRTPLRALLPFFRRRRKANRTIGAVKKVDKMKTASKCYFPKDRSTPKEDEYGWGICLNSNMHGDLGNVDFIFTDKTGTL-TNNDMSFNMCSI-GGRTYGGRR
PfalB -----------------------------------------------------------------------------------------------------------------------------NTDSEKPKVFNKNKNDSLGNVDLLATSRNGVL-VKRKELLVSCVI-NNVMYGKKD
Bdiv  ------------------------------------------------------------------------------------------------------------------------PTAPDPLVPTSWPMSPGFFEELGMVDIIFCDKTGTL-TCNELTMTHLVVGDKI-FNIKD
Tpar  ------------------------------------------------------------------------------------------------------------------------KKEPNEVTGGTWTLNSGLCEELGLVDFIFTDKTGTL-TTNNLSITLVTILSKS-FTPKQ
Esti  ----------------------------------------------------------------------------------------------------RSRAPSPSSPTMRSFAEAKGDSTWPS--VHTPNIIPD-LGQVDFVFIDKTGTL-TGNDMTFSMCSVV-GRIYGMKD
Crya  -----------------------------------------------------------------------------NLNLTNNESRNNSLLLASKYLSKINNSNLILDNKNKEDINNNNKSKLECFFHKYNSLDSFGEVDFSLITQDSIM-EAEPMRLKFISLNNGQEYIFKN
Pgig  ----------------------------------------------------------------------------------------------------------------------GAVDDVETRQRCIVKTVTNLPDLCVSDIVFAS-KCGTLTKSQKLTFRACSV-AGWAYGDRF
Vbra  -------------------------------------------------------------------------------------------------------------------------ESEKLAATGAKARTVNLNDDLGQIEYVFSDKTGTITQ-NEMQFVLCSI-NGTTYGDES
Evor  ----------------------------------------------------------------------------------------------------------------------DESMYYKEGDVHCTVRSSGLCEDLGQVDFVLSDKTGTLTQ-NEMCFKACHV-AGQTYGYWS
Pche  ----------------------------------------------------------------------------------------------------------------------DVHLRRGDQS--VIVSASNLNDDLGRVDYVLADKTGTLTE-NCMLFRMCSV-GGTQFGNTD
Tpyr  ---------------------------------------------------------------------------------------------------------------------EFQETRLTESTFTPIDDKQQGDMCLTTHAIFS-KSGTLM-H-NEEFILSSVTIGNKMYKLNI
Bsto  ----------------------------------------------------------------------------------------------------------------------DLEMFNEKMNQPTIVKTIDLNEDLGQIEYIFTDKTGTLTE-NQMEFKKCYI-GEKSYGFLE
Pram  ----------------------------------------------------------------------------------------------------------------------DPKMYCSITGRGVTARTSNVNEDLGQIKYIFSDKTGTLTK-NQMLFKVCSI-GGIIFDGS-
Ainv  ----------------------------------------------------------------------------------------------------------------------DPEMLDPVTAKSVQARTSNLNEDVGQIRYLFSDKTGTITK-NEMVLKLISV-GHVVYDNI-
Croe  ----------------------------------------------------------------------------------------------------------------------DPHLRSAQ-GDAPVARTSSLNEDLGEVDFVFVDKTGTLTD-NKMECHAVVI-DGVRFGKEG
70%                                                                                                                                  ζζ ζψζ-ζLGζψ-Ωψφ DKTGTLT  Nζψ       ψ      Y
cons                                                                                                                                        E LGQ       DKTGTLT  N
2o                                                                                                                                            hhhh  bbbbb               bbbbbbbttbbb
                                                                                                                                               h1P   b1P                  b1N    b2N

PvivA QKGKKAKTGKTKPSNCSRTGSSHNGDNVSKKHGSSNRFFKKRDSEDAMVKKISFATNICNESSDEFEQAAEGARKKRDHFPHPHEQRRGKNASENQSENAHTHDADNPKKGDSMFPNYKSSSFWNYQSKSIDRKMSGNSLNECPYLQNSATLSSSISPMSSSRF
PfalB IICSRTNFKLPTLN-----------------------------------------------------------------------------------------------------------------------------------------------------
Bdiv  DIAKLH---------------------------------------------------------------------------------------------------------------------------------------------------------------
Tpar  LVQSYQPASSSNISTNGHTPEKVN---------------------------------------------------------------------------------------------------------------------------------------------
Esti  FRGAWQSKECKTNSELLDASMVEQLRAARADTHSSSMRRHSARSLWTTGHGPHARGVAG----------------------------------------------------QRTSAQEGSSAGGPLNVAFSTAGLHGRGPPLGSSSPRRESD
Crya  GLEVNASPKFSIISGLVWRHHSISWHRNEPSRFINGHMRVVRPNIRSSMFIYKIIHPKK------------LELFNYHSVISAISKLDIFKENNNNSLISDSKSNTKNSEDKVKMRKYYKKTLFKMKELKNTYLDEPSESNIIEYRNTWPPLSLVRTKKSFTN
Pgig  SNQFLDGESDGELSTMRDVRFQMSSHLPSESRTTGEDSEH-----------------------------------------------------------------------------------------------------------------------------
Vbra  LMQDTSLPTIEALSDEYQAEETAGDDPPDAPMAMPNPDK------------------------------------------------------------------------------------------------------------------------HVHTT
Evor  DTPDESREGDTSQGINAEFFGYPSDHIERIPSRSVCFPLSPYMWNVPY---------------------------------------------------------------------------------------------------------------------
Pche  SMIDSRAITSLTSSLKT----------------------------------------------------------------------------------------------------------------------------------------------------
Tpyr  DTLTEVINTKGKPSLQYENGANMIDPKIPSCEAIDAEEGVINNSPTVKRATISTNKQNL---------------------------------------------ERMQACDFEMIALRNNTIQSQTQPSQTTATAPNYEAYEDTLLSKYKEPQQRKE
Bsto  PQHSTRQTQ------------------------------------------------------------------------------------------------------------------------------------------------------------
Pram  NMQLRRAAQASTDDLPGGSDMMLKFGS------------------------------------------------------------------------------------------------------------------------------------------
Ainv  -PVREKRVDGVHRGSFAQGDIQA----------------------------------------------------------------------------------------------------------------------------------------------
Croe  ASPAVSSSSAAEAGADAPAGPGVATGGEVSMT-------------------------------------------------------------------------------------------------------------------------------------

PvivA SAPSSSPCSSACTSAYTSAYSSSCSSSGLSSSSSSASYLSSCGVHHNANSSFLCSSSNNEQHEMPNDFDSLRSYSELGREKHNRTRLQDDTREEDTGRGGGSDGEKYPRESHRESHSESHREPHRESADLSYFFDRNYQTYLTQHKFSRPQNFRINMNTGSVKA
PfalB -------------------------------------------------------------ILDSERKNVSNLLNLDERIFKDPENIFFPTRDFYSFLKLFENKISSIYNPYSSSLSNLLKEKYKNYVNEEILNKNVKLTSFVKSQLTIGYNQICEDD
Bdiv  ---------------------------------------------------------------------------------------------------------------------------------------------------------------------
Tpar  ---------------------------------------------------------------------------------------------------------------------------------------------------------------------
Esti  RSPPSSEFPEVSSSNFTGGNGVASPFSVRWDSGPLNCIRTLEDNSEACRGFFPFSSFDLEGNKSAHQCEGLLARSYSGLHANAVELGGFAARRRAATPSSSESSSFRGVHVSPRGAAQEDGEHSDDSSESTSKKEEKLRVPFLYDNITPPTQFRDTPRDGQIRR
Crya  LNKKESKTYSNRINNKSYLYHPALPIHLFPKRLTILLNQIVLSNKKIRSTNFKLTTKNSDIDLNDNEYKRENSVFGEKSKLINKEENEEILYLMFKNIIKLTNSFYGWKTNQKYELFDEYSAIIKNLLIETINYTLINSFCDTISENKRIEYSLSNDLNVESKL
Pgig  -----------ESGSNDADITSWLTEKDSGLLESCESDCAYPDADVELTSWTTDFSAPMSLRSVDDSDTHNSPAATPRPAMIPYSRGRRSSLWSISSLPANQATRPPPVAGDDDLNPPEPTNENWPPRPERRASISVFSCLDFDSSDESLDSCPAVTQHTVSDV
Vbra  PSAHTDHQHQYQRKTPSPPSVGTRGSIIRTIRTSTGQAVTRDIVAPSPSPQTHAGRRIQRRMSTKSDKTVMDSSSQKTFAVDPALGGAAGGCPGSSQKSIQRSPSSPPPSASKSAPEYAVVRACFTEQHTRSNWASYKTDGTHTSNRESDLGGTTVTVNMYPRK
Evor  ---------------------------------------------------------------------------------------------------------------------------------------------------------------------
Pche  ---------------------------------------------------------------------------------------------------------------------------------------------------------SLTFSAAEKSC
Tpyr  NGESYQFSQNGQNNSEEKNSPASKYEDSPYFNHRVEIGRFSSSPKSYLSGDYN-EPDQIQLRGQAYSEQPSVECSPTEFHSKLENLFLRQKNDSYSISQSGINTLDSSNFNGGIEKFYKNSPQQNSSYTIEGLRDSQTPLDFSSSFHNTSKMGQNSIDNKNDLE
Bsto  -------------------------------------------------------------------------------------------------------------------------------------------------------------------LYH
Pram  -------------------------------------------------------------------------------------------------------------------------------------ASAVRGSSAGRDGNTDGRRKSKHVSVTEKDVRSYS
Ainv  ---------------------------------------------------------------------------------------------------------------------------------------------DRISTRLRAYSAHVMPYSMLTQHGTYPD
Croe  -------------------------------------------------------------------------------------------------------------------PERGIPPAVRSSSGPQTAGAALFAAPVGPSVTEHIDAVPFGDSRLLALLKAGVEA
```

```
                              N-domain
PvivA  RCDFDDKKIYEDLNSNTWRGYYIDEFFKCISLCHAVVPFIQFDYIRNKNLTHFPSRSSKGVSQYMEKYNNHPKQQSNDVVKMRNELNLKTSMEYDCLEEEEHRGVGKFEDRQTKWITEMSDTAKNAKKKNETNLYNYNTLNNLKHKNEKKNNIKFKFLFNKKFR
PfalB  ELSYNCYEIKEDSQKENIQSVKIEDFILGLCGCNRIIIYNEKSLDISMNEYKSDNFMETYSKFETEN-------------------------------------------------------------------------------------------------
Bdiv   ----------SEFEKDGSRNDQLDKLLKLMTLCNASIAVTSSHNLDTFASPSRMATSSSQAYKPYGSRNSDLGCSIPSSINYPLRPSKSKIHFGNVSY------------------------------------------------------------------
Tpar   ------------GKSNREHSEESEHFMRLLCICNPCLNMDSEDGYNSYSSGYKSNYSRAYSFKLSHFKTKSFDRFHFNDSGENFDENPHSVNLDDHFDENSQGVDNSVENEEEDE-------------------------------------------------
Esti   NCDFYDMQIFEDLASADERSHRVNEFLKCMALCNTVVPHLKGHVGSGEALLPASPVQSWTASTGPHTAASALARSMGTTAPISMLGQRGNTRASAVALRDTAAKLGDDRG------------------------------------------------------
Crya   GANYESRNDEYYCDSNINNS-VLIDMLYAMATCNSSVPHLRILDTKYKGIDESELYPNNKIKCGKNNNMNDENKILHSTNNIIRSLSRDSNYKFNYSSSDENGIKY-----------------------------------------QLSRYSIKNSLVSFNDL
Pgig   CQFRDPDFYMHWRTGTVWRRNKIDEFVFAMAVCNDCRPYVELAGVSAAIRKAVDDASR----------------------------------------------------------------------------------------------------------
Vbra   QSVFADAFLKQDLAAGGNQAHYIDEFLRCMALCHTVVPEKNESG------------------------------------------------------------------------------------------------------------------------
Evor   ----DGEPGLGLP---SPNNHLIHNFFLCLATCHTALPERMPPVTPQKRSNGLMQSLQDLDFVSQNDPAMQCLRDEAGVENRMQ--------------------------------------------------------------------------------
Pche   MRDEALLDLLGVPVIVTQDHAFAHVFMLTCALCNTVIAEPSTDSP-----------------------------------------------------------------------------------------------------------------------
Tpyr   FIQEERDFLRDLQKNDQMFASAIKAIGV---AHLTKTK------------------------------------------------------------------------------------------------------------------------------
Bsto   HKKFKFDDPQLWEDLQGEGKAEISAFFEGLALCHTVIVDH----------------------------------------------------------------------------------------------------------------------------
Pram   QMMKFVENPSRFSAKEIHAVELARPFFRCLLLCHSASVALEPGVSGDQSADTLSATGRRTESVEGSMR------------------------------------------------------------------------------------------------
Ainv   HGAGCEETSVVDDNCPLSRGMLAQAFFRCVLLCHTATLSS----------------------------------------------------------------------------------------------------------------------------
Croe   ARLMRLDPASAAICQPTDLTAQAMLFFYGIALCHSVTSPS----------------------------------------------------------------------------------------------------------------------------
70%                             Fϕ  ψ ψCζT ψ
cons                                   CHT
2o                         hhhhhhhhhhhhhh bbb
                                h1N        b3N

PvivA  KKKKSYTSFASSEIYKGAEKKHTGRWNTYLNMKKSKHLFSNEKDNKKSESAYISKRISAVSFKQISKEKLLSLNSYASNRNSEGGAPGVVKKYKAKSNNPDEKSFEFASFVNERGYRNSGGNGGLNNSVHGGPNNSANGPPPMYDDGGTPKRTEERNGMQHSDF
PfalB  --------------------------------------------------------------------------------------------------------------------------------------------------------------------
Bdiv   ---------------------------------------------------------------------------------------------------------------------------------------IEPMASVDFGLKELDLSHNSIDLSSETVD
Tpar   ---------------------------------------------------------------------------------------------------------------------------------------VENLPMYKKTKSKAHFGTVSYINDENVSK
Esti   -----------------------------------APPGGGLWQFPSNPFVQSAAHRSTSLEANGVAEPGHMPQAVLPRGDSCDSNPSPELMRKATKRRKSLGRGVSFKDTHEEYSFLKDGDETLADEPSQSFGGLGMASKASGDFPSLGRSSSPKFVRFLPAA
Crya   STKKSGSKLHFKGKRTIKNKIKALKLLQRKRIANLFGDKRVDKLKIYKNSNLRLYSHDTNEITVNDSSENNNLLTYRYNKEYEKLHNQRRKCNLVTLPREMMLTKYFIKYTVNMRNDISEYKDNNINTILNRIILNKNKSKLKHKKSIFSSNRKRIEFKNILKR
Pgig   --------------------------------------------------------------------------------------------------------------------------------------------------------------------
Vbra   --------------------------------------------------------------------------------------------------------------------------------------------------------------------
Evor   --------------------------------------------------------------------------------------------------------------------------------------------------------------------
Pche   --------------------------------------------------------------------------------------------------------------------------------------------------------------------
Tpyr   --------------------------------------------------------------------------------------------------------------------------------------------------------------------
Bsto   --------------------------------------------------------------------------------------------------------------------------------------------------------------------
Pram   -----------------------------------------------------------------------------------------------------------------------------------------------------------AESRRQSGG
Ainv   --------------------------------------------------------------------------------------------------------------------------------------------------------------------
Croe   --------------------------------------------------------------------------------------------------------------------------------------------------------------------

                                                                                    N-domain
PvivA  DLDKCRDPRITDNYASESNAYLDAIKYQSSSLDEECLIYTSNYLGYRLILRNKNTMCVEIDG--------------NLHKWT-IIGINEFTNKRGRMSV-VVKPEF---------------------------------------MQSGSVLYVKGSDTSILSL
PfalB  -----EEYHENDHDEYHNMEHSDDENINSIEYEDICLYNIIRNTGFSIYCYKNT--LFLYN-------------LMKECKVYFLTCYHDFLRSNK-FSMCILKCGYSINN-----------------------------------EKEGGILYVRGYDFNILPY
Bdiv   NDHDDSFMSSSYPSRSSRS--SNTLYYRSPFKEDECMMDMANNFGYKLAHRSAKQ-IQME--------------VFDVMETWDIIGFNSFSSKRRRMSI-VLRKQ----------------------------------------NQKGSDVFVKGSGETMVTL
Tpar   ELFDLS-------NRESLK--EERRYLTCISKEDECMVNLANDCGYFVSNRTRDR-ISVN--------------CNGKHVNYEILGVNEFTSSRKRMSV-LVTNI----------------------------------------QNNENFLYIKGEAYSMLSI
Esti   CAASISKTSGSSRSQSDSSRLLRYIKYQASSPDEECLVSAASHMGYSLAARDPSS-VVLQ--------------INGQSRRWQVIGVNEFTSKRGRMSV-VLRPAG---------------------------------------WSEGAVMYAKGKLSH-IWA
Crya   LKYSFKSDKNEKVNRYYESHPKSYVQYSGIDENDLTIVNTAASFGMRLVLVNSQH-IVVE--------------SLGRTIFASLIYKYEDSNNDM-ISI-VVKFP----------------------------------------QIRESVLYTRGLAKNMLPL
Pgig   ----------WSTRIRTESHNLNAIRYLAPSREEESITMTASFFGLTLVSRSPTHLTLCH--------------DGEMTNYSVIASFP-YTPKRQRGAVCIQRK-----------------------------------------GEMGATLYVRGHPAQLAER
Vbra   -----------------------TMKYQSASPDEEALVLSAKDIGYELVA-RTSTGMVLT---------------IMGRTVSFEQVGVNEFSSTRRRMSV-IVRELREDGS----------------------------------DTDEGATLYCKGADSVMLSR
Evor   -----------------LLEEANSTAFRSPSPDEEALLAMARDFGFYYKKRNGNR-LTINI--------------RGQEVYYSVLMCNEFSSERKRMSV-LVRQCSGNDPPANPQSSHMVSAFMAPQ------GVTSMENLGGQLEDGQYILFAKGADSVMMER
Pche   ----------------------FGIRFQGASPDEEALVEMAASFGYILVDR-GNNFITLRVSRRLVSRHDSEIDRQWSETVFKVIGINEFTSDRKRMSV-LVQPLQVVEAENGDTTYV--------------------------PESEGSLLLVKGADDVILGL
Tpyr   ----------------YIYSQHQFFTETLNPQDKIIQEFIQQ-IGFQFRCSNKMIDGSKKRF-----QYIYTNIQNETKRFTFLI-PPAFNERKTRLCV-IVK----------------------------------------EDDSKKYTLYLKEELVNFPNY
Bsto   -------------------KEDETV-YQAASPDEEALVIAAHCFGFSF-ISSKNK---------------IYTLKINNECMDYKVLGINEFTSDRKRMSI-VVV--------------------------------------PLSDRSRKPFLICKGADNVMLE-
Pram   FNSFTSNHAAAAEASDAFPSLKGDRKFFGSSPDEVALLNAALEFDCVYERREG---------------DVIHIRLFGKSESYQLLALNEFDSTRKCMSVVVKRLSRDEADQRSSDPKLPRVRDSLGDLEHSLEAELDGLNGDYEAQEQDILVFSKGADTVMFA-
Ainv   -----------------------LNEIRASSPDEETALLRGAIELNCLLKGRTT---------------TTMDISLFGKPESYEILALNEFDSTRKCMSVIVRQLTAP--------------------------------YRESITKDDVLWVFCKGADTTMMS-
Croe   -----TPTTSVSAAAPAAPSLPPWRLYRSTSPDERALLLAARAMGFGFIGRQG---------------NKLVLEVMGQVETWGLIHTHPFSSTRRIMSIVVRS------------------------------------------PTGQVFVFAKGADSAMLPR
70%                               Yζ  SPDDζ ψψ      GF ϕ                                 Ω ψψ  ζEFζSζRK MSψ ψψ                                                ϕψY KG D
cons                                  SPDE A                                                     F S RKRMS                                                         KGAD
2o                             bbbb    hhhhhhhhhhhhhtbbbbbb                       bbbbb bbbbbbbbbbbbb          bbbbbb                                           bbbbbbb hhhh
                                b4N          h2N      b5N                          b6N          b7N              b8N                                               b9N   h3aN
```

```
PvivA LNLKYSRFLERGGKRRRRKRKAHKERRRVRANPTGSPSSKGRHSRKRDQMDASQGKSSASGINTDCECHQCGDHSHHAECHQCGDHSHHAECHQCGDHSHHAECHQCGDHSHH----------------------------------------------AECPH
PfalB IS-------------------------------------------------------------------------------------------------------------------------------------------------------------------
Bdiv  LRSS-----------------------------------------------------------------------------------------------------------------------------------------------------------------
Tpar  I--------------------------------------------------------------------------------------------------------------------------------------------------------------------
Esti  DAAMLPLLSSFEGMSYEDTLGTKYEGGLDPSFDESASAKNSPTGGGMQNGSSRSFNRGATRDPESSSRLGHSLTLTYDTFHEAYHNLYKYSDQRGMSLKSVRGASAGGRFEADA--------------------------------------------------
Crya  INPQSIKSNVESSSNNNYKVNGSLCSDYN----------------------------------------------------------------------------------------------------------------------------------------
Pgig  LCTS-----------------------------------------------------------------------------------------------------------------------------------------------------------------
Vbra  LAPG-----------------------------------------------------------------------------------------------------------------------------------------------------------------
Evor  MRQDDQETSNSRCLNMDMEVSDKLLAESPRHNLNGAIPEGFEAAMASEAGSEGDRPATPDPAPPAPTEPAPPLSLEVADPQGLASVELGAHVASAHLGVPPAPKSGASSFQDDRSYLAAAASGM----------------------------------------
Pche  RRKSDDEAPFAELCG------------------------------------------------------------------------------------------------------------------------------------------------------
Tpyr  ILLE-----------------------------------------------------------------------------------------------------------------------------------------------------------------
Bsto  ---------------------------------------------------------------------------------------------------------------------------------------------------------------------
Pram  ---------------------------------------------------------------------------------------------------------------------------------------------------------------------
Ainv  ---------------------------------------------------------------------------------------------------------------------------------------------------------------------
Croe  LYNSDVARREAARAGPLPTVHSAEASMAEPSHSSDGVQPAYVDGFAGSDGLLSPIPHEDSFHVRSGNHHASAASAAIDPRMRGRLAASPALLPPAVAAGSLLEGGSSHGALDEKERASAGTRTPQTSAPSPQLAPRVHVGHRSRTSSSPEGRGLHRPGSASSVY

                                                                                             N-domain  <-||->  P-domain
PvivA HGTDVHLEADSTGRSESCRFAKRYRQLEKQLRKFSVKGLRTMVFAFKYLNEEETVKYKKMYDDACVSI---YNKEERLEKVAETVETELTYLGITGVKDRLQKKVPKTIEILGQSGIRIWMLTGDN----VEYSLHISFLCKILNSHTKIFHAML---------
PfalB ------------------KEKNNIKKIKNVIKIYTLNYLKVIILCKKQISNEDIAKYIILKSISKKL------SFKFYDLIKLFFLYDLEVIGIIGLKNQLREGVKETFNDVINFDIKSWIFANEC----SKDTYLTALQCNLIVSSSNLFLINYY--------
Bdiv  ----------------TEEQRSEISKLSKSIQKLTSAGLRVIVCAHRVLSREETQAYQRNFSDAVNS---VYSAETLHEEAAFMVEKDLTYIGMVVMKDEIQPGVPETLELLMEAGVRVWMTTGDN----RYSALEAAYQSKLIVNPCQIFDCKL---------
Tpar  ------------------NDQNTKLAIESRLKKNSIFGYRVIICGYRSMTSTEVLEYESSVLMHR----------DGLDELCSRFEKDVNYLGIIMFRDEIQRNIVKTIDLLMDSGIRIWVLTGDN----KEATIQTCYLTKLLVVPTNIFIIQI---------
Esti  ------------------------KLVEQHLKVFSLQGLRTMVLACKYMSQEETDAYQRLYNDAYASV---YSREGRLEEVAEQFERDLEYLGITGVRDKLQAYVPETLGLMMEAGIRVWIATGTSALLASFYTLHICHSCRLLTSQTKVFHAAL---------
Crya  ------------------NRIKSYDDLLYITKKLDYQGYKVYIYAKKVLSKTELNKIRLRRNLTEKNENFKFLLNKSTEDYYINIRNNLDYLGYIGIEYRIQDKVPKTIKSLMEYSIKPWFLFNIS----STNSIRLMHKIFSNYDKIPLIRMDRLPLSNKRKT
Pgig  ---------------HDSKENAVYSISPHHIARATTQGCRAVMFCKKELSFVETESYRMHAADESI-------SNQEKTQYSSDLESNLQYLGLAFLEDDIDDNSTDLVRFFRDTKKQVWLTSGDT----MDHCLTAAYGAQLLEPAAHVWRVSL---------
Vbra  ---------------------QDTSTVAKHLQRFSINGLRTLVLARRVLTQQEYVMFRRQYLEAKTTL---IDRNVRLEQVAERWESRLELLGATAIEDRIQEGVVETIRALLYGGVKVWMLTGDK----QETAINIGHSCGLLETDTTIIYITI---------
Evor  VSWSVRTQPSQEVNVEENKQQKKLKAAKEQLRHFSTHGLRTLVCGYRVLPDAEAECYIKRYGEARGTF---FNRDELMAKVMDEMERDLDLLGITAVEDKIQIGVPETVKMLLDGGIRVWMLTGDC----VETAVNIGSICRLVNKDCRMYYLTSEDGADQSL-
Pche  -----------------VTVEDVADTTVLHIEDFASMGHRTLMIAARHLSVDETTEYLESAKEARHSI---TNRANRLARIAEKFERDLIVIGATAVEDKVQGGVLATVSRLLSAGVKVWMLTGDR----YETALNIARSTGIFPEEPRLLNLSH---------
Tpyr  ------------------KGLNRQDQEKRLKKLIELGQIPIAYCKRELDELEAYQIQNQYENAIKVPEY-YQNKEQIINYIYSPLLTKLDLLCIVGIQEVIRKDSAELVNLLNQSFIKTWMLTGDQF---QKAVPAAYSCRILQKDSQCQTILFD---------
Bsto  ---------------RCLGTSNEIEKLTVNSYEFSVSGLRIMVLAKKELSDEETQAYEKGYNDAMHAL---NDKDKRLADLAEELEQDMQIVGVTAIEDKIQENVPSTIATLIDAGIKIWVLTGDK----QETAINIGYSCKLIKSNMNLIKVNA---------
Pram  ----------------NANKNQDISKLALHVHYFAAMALRTLLLGYRTLSLTEYLEWKCEFDAAKKAL---SNREQLCVEVAKRIEKSVKLLGATGVEDLLQEGVRECISQLSLGGINIWMLTGDK----DETAISVAHMCGLIGPKSKIIIVKG---------
Ainv  ----------LGNATTQGSKPQTRDSITSHLHYFASMGLRTLVFGYKRITLAEFMAWSAAHAKAKTAL---IDREGKLFECARSMETRLTLLGATGVEDQLQDGVTECIETLSAAGINIWMLTGDK----DETAISIANSCGLLSDQSQLVVLNE---------
Croe  RPGSASAAADPLPSMGINATSDARVKLESSIADLWEAGLRTMVVAARQLTAAEEEAWRRATRTAAAS--GRKNRRLMQKAADLVEFGLVGLGATGVHHQLQEGVPETVETLRQSGCRVWMVTGDN----EEPAISVAHAAGMIREASTIVALNG---------
70%                             ψ ζζψζζϕ   GϕR ψψ π R ΨS  E   Ωζζ         +   ψ          E ζψ ϕψG TπψζDζψQζ V  Tψζ L    GI+ψWψψTGDζ    ζζζπψζψπζ   ψψζζ    ψ  ψ
cons                                       GLRTL                                          E      LG T  EDKLQ              G   W LTGDK
2o                       hhhhhhhhhhhhhh   bbbbbbbbbb   hhhhhhhhhhhhh          hhhhhhhhhhh    bbbbbbbbbb      hhhhhhhhh   bbbbbb          hhhhhhh        bbbb
                              h3bN            b10N           h4aN                    h4bN           b11N              h2P         b2P              h3P            b3P

                                                                                                                                P-domain
PvivA ----------------------------------------------------------------------------------------------------ENTNCKKLKREGKALYELFQLEKEEK-KSHEKMCLLINGKNLQTFLNHSD---------------
PfalB ----------------------------------------------------------------------------------------------------------NLKNTHEEGANILFHNFISSLYKLKSNSYAVVINDESIKNIMTNVE---------------
Bdiv  ----------------------------------------------------------------------------------------------------PEDKTDETLVKYGAALYDLFLQQRGEKASSEQLCLVVDGTDLRDFMYNSE---------------
Tpar  ---------------------------------------------------------------------------------KDLLADSITPKRISDSLGSDTDTVTDKIDMLVDNIYNKYILERNN--SNEQLCLIIESDDLEIIMQGNK---------------
Esti  ------------------------------------------------------------------------------------------------------ATRGKRAKREGILLYEMFRDARILKKADEHICLVVTGPNLAAFLNHPD---------------
Crya  VAFLVKIYNELKIIVKNKYLILKDFDFELDSITEGDYNSDSSNKGNSCQSNNNNMNRRTRKSILSLNKKYSDVKFLNNLITISTLNGEFQLSQFYNNNATSNLCEIGESVNLNEWMSWFGNFPGHGVVNSLPGIIFSAHQLTKIFQSRN---------------
Pgig  ----------------------------------------------------------------------------------------------------------LSNSGVQQTLVKLVKQMSINVFSDASRQAIAISGSDAALCMGHEG--------------
Vbra  ---------------------------------------------------------------------------PKPEDKNKNKNKETTEEETPPKPEKSKEKSEKDKAKEAMMGYYEDCIETLKNPQLSRHLAVVVDGETLYKIFDDSE--------------
Evor  ------------------------------------------------------------------------------------------------------EKTRDDLLNKLRRMEACVIEGLKNHSAEAFKYSLVLNGAALYAILEES---------------
Pche  ------------------------------------------------------------------------------------------------QPECTSSEATPDDIVDAKRSEFQKFMHGEISDEVAKSPCIVLDGPVLVYFATSR---------------
Tpyr  -------------------------------------------------------------------------CNNYKSLKSLIKNYIVVLKSKYIGESQIKKNPNDQSTQNAFTRKKTSFINSEEDDKRSIFNIIISGKTFNLIRKN-----------------
Bsto  ------------------------------------------------------KSLDETRNILRGNLTKHISGHEFRRQTSVLDQIRYIGNNNTPGNTVIGDKMMPRYQFVKHDTDLLDGDIKVKNPHLLNQALIVDGLTLSFILSD---------------
Pram  ------------------------------------------------------------------------------------------------------KTKQDCLDEIAKARRKLKREGFWVPGVASQDISLVINGEALEFLLADALTSSAIKAQISYVNA
Ainv  ------------------------------------------------------------------------------------------------------TTQQGCMYQMTSIRKQLRKQGLWKPDVPSREIALVINGDALECLLNESRCEKDNEPGSTSRFA
Croe  ------------------------------------------------------------------------------------------------------DSRSKCEHQLATARAELRSKGRWHPRLVDDGLVCVINGEALSHILAPSPKARRAEHEEIAQRH
70%                                                                                                                                           Φψψ ζ      ψ
cons
2o                                                                                                                                          bbbbb    hhhhh
                                                                                                                                             b4P      h4aP
```

```
PvivA -----------------------------------------------------------------------------------------------------------------------------------------------------------------------
PfalB -----------------------------------------------------------------------------------------------------------------------------------------------------------------------
Bdiv  -----------------------------------------------------------------------------------------------------------------------------------------------------------------------
Tpar  -----------------------------------------------------------------------------------------------------------------------------------------------------------------------
Esti  -----------------------------------------------------------------------------------------------------------------------------------------------------------------------
Crya  -----------------------------------------------------------------------------------------------------------------------------------------------------------------------
Pgig  -----------------------------------------------------------------------------------------------------------------------------------------------------------------------
Vbra  -----------------------------------------------------------------------------------------------------------------------------------------------------------------------
Evor  -----------------------------------------------------------------------------------------------------------------------------------------------------------------------
Pche  -----------------------------------------------------------------------------------------------------------------------------------------------------------------------
Tpyr  -----------------------------------------------------------------------------------------------------------------------------------------------------------------------
Bsto  -----------------------------------------------------------------------------------------------------------------------------------------------------------------------
Pram  NQVIPHASITPHSDPTYHHSTTPDGLRSERDARLSGSMSSKSMG-----------------------------------------------------------------------------------------------------------------------------
Ainv  DTNNESTHSLFGVDTNLVPGHRDSSF-----------------------------------------------------------------------------------------------------------------------------------------------
Croe  RRQRLRSGATPADIDEESDSSSDSDDEAAEEVVGSGASSRSARIRRSGGLCGRAGCCRGSKRRRANRSERVGTDSHNATDRESTTPDTSVATGGTPAYGNLDGLAGGSLDDPSCVAACFCASRGCFCCCCAAESASSLRAGRRTIVRQVSEGAVSAGDYSARNL

                                                                                                                                  P-domain
PvivA ---------------------------------------------------------------------------------------LQTHFLNMACSSDVVIACRITAKQKAFIVQLIKHRLTP-TPNTLAIGDGANDIAMLQEANIGVSIMTPERIISSG---
PfalB ---------------------------------------------------------------------------------------SMKIFLCIAMRATVVLFCKLQNETKGKIIRTLYALTSP-KLTVLGIGTTLNDAYLLKYSSISVFLSLNEHVNILYN--
Bdiv  ---------------------------------------------------------------------------------------MQTYFVNMLCFSDVVVACRLTPFQKAEFVRHVKTRLMP-TPVTLAIGDGLNDVRMMNEAHVGVAVLGASADAAA----
Tpar  ---------------------------------------------------------------------------------------GQQIFMNMACSSDVVIGCLLTPIQKGQLVQMVKTKLIP-TPITLSIGDGINDVQMLQQSHLSICILNTASDASNNKKN
Esti  ---------------------------------------------------------------------------------------LQTCFLNMACCCDVVVAARVTPTQKADMVRLVKKRLTP-QPITLAIGDGGNDVPMLQEASVGVAIRTVRGTSVAG---
Crya  ---------------------------------------------------------------------------------------LSNYFFSIAIMCPYVVISDSISKDISSITDYLRNNVIPKPVILGISGNTGNVSTLSSC-DISISVSNTESIHNKNKSY
Pgig  ---------------------------------------------------------------------------------------TEALLLYLLSMTEVVIAYDFPPSLRADIIRAVHHNV-HRRPVTLAIGQGAGDGPMLVEADIGIAISQPTA-VFAPKLA
Vbra  ---------------------------------------------------------------------------------------IKGMFLGVTLRAHTVIACRVAPAQKAGLVHLVKSCV-RRQPMILAIGDGANDVSMIQEADVGVGIIGNEG-MQAVR--
Evor  ------------------------------------------------------------------------------------AMELQQLFVTVVCNSYSVIACRLSPAQKAAVVELIRTQV-PGNPLTLAVGDGGNDVSMIQKAHVGVGIAGAEG-REAVN--
Pche  -------------------------------------------------------------------------------------RNLATLANILIR-AERVVACRTSPAQKALLAQLVKKT---GSCTILTIGDGANDVPMLKIADIGVGIIGSEG-MQAVR--
Tpyr  -------------------------------------------------------------------------------------QYLNDHFTFLLYFTKNLVGYQFDQFQKTEIIQNLKICL--PNAKILGIGSNISDLRMLTASDYSIEIVDFQKKEIYAR--
Bsto  -------------------------------------------------------------------------------------SVCLRLFVILSTLCKSVLCCRVSPLQKSEILKIIKNHL-VFKPMTLAIGDGANDVSMIQEAHVGIGIVGREG-MQAVN--
Pram  --------------------------------------------------SLPFQSPNMALNSRHRSSSRPGSTRTSITDTNGGVQLAHELFLELASQCRTVVACRLSPMQKAQVVSLMKAA--PGTPLTLAVGDGGNDVSMIQEAHVGIGIYGHEG-MQAVR--
Ainv  ------------------------------------------------------------------AHTHLSVKGLQDQPNTNENDAALALFMQLVTQCCSVVACRLSPIHKAQIIALIKGS--KSRPVTMAVGDGGNDVSMIQEAHIGVGIFGHEG-MQAVR--
Croe  ESAVGATSTCRSEDGGHTSQPTCRCCSREWVCCHNLGSAHGRLFGSSNIDQGTFMWWLLHPVIWFPQWLTAALGISDVTLEAEPLPRERRLLELLKQCRTVLACSMTPAQKAQLVATVKSMV-SPGPVTVAIGDGSNDVAMIREAHVGIGVTGGDC-THAVR--
70%                                                                                                  Fψ ψ      VψψCRψTP QKπζψψ  ψK  ψ    PψTLπIGDG NDψ Mψζζπζψπψπψ
cons                                                                                                           CR  P QK                     GDG ND  MI  A  G G  G EG   QA
2o                                                                                           hhhhhhhhhhhhh bbbb  hhhhhhhhhhhh      bbbbbbbbb   hhhhhhhh   bbbbb
                                                                                                  h4bP      b5P       h5P             b6*P      h6P       b7P

                                                                                                                                             P-domain  <-||->      S5
PvivA --------------------------------------------------------------------------------------------------------------------------------------------YSDYCIKNFCCLRKLLFIYGSKHLY
PfalB --------------------------------------------------------------------------------------------------------------------------------------------ISDYVLQEFKFISELLIL-GRLNRF
Bdiv  --------------------------------------------------------------------------------------------------------------------------------------------YADFVTSHFAGLRSLMFYQGSSSLQ
Tpar  NIQFGGPSNSFNANNTRTGKLNVKLNVPLNGPLNG--------------------------------------------VVNGVPDKDNTPDSADKNDTAESSETTANRHQDKGLGLEKSVYSDSLDTNEKLVINNDIIGYCDFCVDSFSNIRELLFKQGTRMLK
Esti  --------------------------------------------------------------------------------------------------------------------------------------------FADFAISEFRFLQRLLFVHGRLNFM
Crya  DKSTSNINISINNNNKNNNNKHHHHNNNNINNCDEETSKKRDSISINSRDINRSKDSFFMKDLNNNNNELYYAIRNKISPGIYTTTDKNNENYNYNNYNDITKNNSKSGKKCGIKSDLPRSIVSRRKIRQYINNTILTPVTDVTISKFHQVIPILSRAGRITSY
Pgig  SLDSHF--------------------------------------------------------------------------------------------------------------------------------------ESCVFWTPGSSASPIVDPDSSRMMD
Vbra  --------------------------------------------------------------------------------------------------------------------------------------------ASDYAIAQFRFLQHLLFLHGRRNYN
Evor  --------------------------------------------------------------------------------------------------------------------------------------------AADFAIGQFRFLRSLLFLHGRSNIR
Pche  --------------------------------------------------------------------------------------------------------------------------------------------ASDYSLATFYHLGELLLVHGRDCYN
Tpyr  ---------------------------------------------------------------------------------------------------------------------------------------------SNSIVRTMKDIIKQLAVHSKNSYF
Bsto  --------------------------------------------------------------------------------------------------------------------------------------------SSDYAIARFQYLLPLLLKHGRNNYG
Pram  --------------------------------------------------------------------------------------------------------------------------------------------AADFAVVTFRDLSRLLLVHGRWNHR
Ainv  --------------------------------------------------------------------------------------------------------------------------------------------SADFAIGQFRFLSRLILAHGRWNYR
Croe  --------------------------------------------------------------------------------------------------------------------------------------------SADFSVPTFKTLAPLMLTHGRQNKL
70%                                                                                                                                                 πDY ψ ζFζ ψ ζLL   GR ζ
cons                                                                                                                                                  D    QFR     L   HGRN
2o                                                                                                                                                     bbbbb
                                                                                                                                                        b8P
```

```
         |->       cTM5       <-|       |->        cTM6        <-|                                    |->       sTM7       <-|                 |->       sTM8       <-|
PvivA TISIILYWN-FFKNIVLILPVFFYQAYASWSCVRIYPEL---LYTFFNIFWIFIPLIYYAFLQH-N-LNYDVLYNIPLFYALSRRKYNMS---TLKFFPWVVEAIFYSVVVFFFAYTALGENSHLSNGEVVTISTFGNICFLGCFLISIVRLFLEGSLWSPSIL
PfalB SLCKVFLWIIYLKITVVSFY-FFHNFDNYFSGSSA-SSI---LYTQTTFALLHYFLIIAFSAYEID-LPYKFVRRLPYIYQLSRRKYFLN---NNIILLTIIEAILISLTSYYILRLNVFH--------LITHREFTFHIFILNVFITTEKILLLSKTWH----
Bdiv  VIAAVTYWS-FFKSLCFVMPIFYYQGQTDWVGLDLYGSF---IHLLFHLIFTTLPVTV-CALYSSP-LSEPTLTNLPALYTLCRRRFHIN---LLRIGFWILEGILSSICCYISIQASGMESPMFGSGGTLSARAFGLLCSLGALEMSNTRLFMELFLRNNSLG
Tpar  STSLIIYFT-IYKGLVFILPIFYYQIFTNWVGLDLYGSL---ITILYHLVFTFIPLIM-FILFYQD-ISYEIYNHISILYTISRRRIYLN---INNSLYWMLEGILGSFVNYLPLHLLVINNDNIYKSEVISNRTFGLLISVSSIIIFNTKLLINYTPNTGNFT
Esti  RISKVILWT-SFKSILLAFPVFLFQPSAFWSAIELFDPV---LYMLANFLWTTLPVIVYSFSEQDL--PEHLLPCIPVLYTLGRRRLYFN---GFTFSFWVVEGIIYALITYYLLQAAWLDSTALPDG--SNLGFFSFGVILLFGLIIQSNLRILME-THLW--
Crya  RTGISLYNN-AFKIFYLVLPHLIFQFYCQWSGTLVYSTD---LMAFFVVFVTVLPPILYGFTGIDV--PPKLI-DLPFLYIFSKNGYFTN---WLTYLYYLSESIFLSIMSFYFGILIVKNTSPFING--ISSDLLNLSDSQTLYCIIGSLFSFVVKSKSFS--
Pgig  PNLRYRLNAEALQVADMVVPN-TDCITTIIKKYSLPCRLTTTVLLHYTLFMAGAVTMPHVVIV-------SRNAFLPDM-EIGRLCNFPN---MLNVLFQPAIFFFCYKNIMPKPLSPARYAFLVNSI--RTKGLNSVWSLLGTNLQGVLFGSLAAGVTLSSEA
Vbra  RISAIILYS-FFKNICLVIQNFFFFNNGHSGTTLFESW---LLLSYNLIWTSLPIFVHGFIERD--LPQDVCMRFPILYVQGQHRLSFN---ARIFTEWALRGVLYGFINSVVMWMAFRRDAYAID---AS-GQPADYYAMGTISFFTTIVIVTVKLAFETRH
Evor  RIGIVIGYS-FYKNFLLVICMACYAPFNGFSGTTFYDSY---LIMMYNMVFTNLPIFVVGALDVD--VYAPAAFALPKLYLFGANKVYFN---YRMLIAWLGRGVVHAFVNFFVATLFLGGSGGQP----AGLTDYLSLGTWSYWSCVLVANSTLLLHAQV---
Pche  RISLVILYS-FFKNIYLILPNVYFAMSNAYTGTSLYESW---ILMSYNVLWTSMPIILLGATDIT--LPRWLCLHCPLVYIEGRRSISFN---ARKFIAWMVRAAFYAGFTYFAASVSMRYSFSLTSN--GKVVGYAYIGTLVYYSVIIVANVTLLVFCNQ---
Tpyr  QKEDLVIMS-FYRSFLIGIILFFWNTYQSSYGISLLP-----PPYLLQLIYFT-PLVLFIIQQLKIHKWKIPYKKEVVDKVQTLIFIIHNQSVKRDNLFQYLKSVFIQSVLEGLFIFLIFIFQYQDSMVSTDGQVLEKEMIGLFLFQTIHILLFYKYLLNRKCS
Bsto  RLAKVILYS-FYKNFMLVLPLFYFSFLNQYSGTTLYDSY---LQVFFNVIFTSLPIVVVGSMNKD--IESRVVMANPFAYIPGIFNKKFN---LVIFIRWSFFAILHTLLIFILLRGADSFTMDSH----GMMSGLDLFGTAGFFAIIITVTYVVFLEMRD---
Pram  RVARVILFS-FYKNMALIMTLFLYSFYNGYSGQTLYESY---LMVGWNVLYTLLPIFVLGITDED--IRDSAVLRFPFVYRSSLRKSELS---IRGLSFWVANALFHSLLVFFFVTKTLGGVSSRY----SRGNGLFPDGTAVYGALIITVTLKASIHMQCLHR
Ainv  RVSIVILFS-FYKNMALVMTLFMYSFVNGYSGQTMYESY---LIVGWNVLYTLFPILVLGIIDED--ISSDTVLKYPFIFRNNQMGQELN---LSKMRMWVGKALWHSFLVFFLGTFLTYNVFGSYS---MADSSIFLYGTAMYGILVVTVSLKAALVMQRLHR
Croe  RTSLTILYS-FFKNVTLVFTLLLFSNLSGYSGTTLYESY---LGAGWNVAWTFFPILAIGIFDSD--LSRQASMRYPIIYRQIFRGAAFS---VRSLAWWVVQGVLYAGVVTLASYAMFEHSWVLSD---GTAAGIWPTGAVVNLILVVLVSGKLVIHMHS---
70%   ζ   .I ΩS FYKζ  L    F Ωζ  ζ ΩSGζTLYζS    L . ΩN  ΩT  P                    PϕψY         N         Wψ ζπψ    ψ F
cons  R         YKN             S                P                    P  Y
2o                                                                 hhhhhh hhhhhhhh
                                                                    h7aP    h7bP

         |->       sTM9       <-|                      |->       sTM10       <-|     Reg Domain     |->       linker
PvivA ITCLGFFLFVFFPSILFILFAYLSNDYIREVFRQTFM--------WTPLYILLTLWFTTCIICYIFINFVKAVMFPNIYSVVNHWLFEQYQEKHRKYQYVYFLSGKKKFINLKKIGRKFKHRFSRRVHKTYRFKPTMKQLHQEDCSTNKKCPAKQPNLQSSHGS
PfalB IYFFIMAVLIIGILLIYVNIFTLVDCIKNGKCEFSLFQME-----NIYFWTSLFPILYINFIFDKLMKYIKNRIYPDISDYLRKYFLRRICC-----------------------------------------------------------------------
Bdiv  IVFSLLFLLAMPPIFVGLCFLCGGQALRNGAYQVAL---------WQPVYFLIPVWITTGAVCHALSALTQAVVCPDIGNYLRHWVPSQKCN-----------------------------------------------------------------------
Tpar  -FISLLYYFASPPLYIGLVLVSSRRSLRLSSQQILR---------IIPFYILIPIWITFTVILNILSNLLQLYILPNLIKHIKLYIINNNNT-----------------------------------------------------------------------
Esti  TPSFLFTTLGLCTLMYIPVIYLYSASGWPSRGIFGVAPG------IFSWPLLYFVILLWIGVGLMLQVLLAGFKASLFPNIANNVKQWLGQR-----------------------------------------------------------------------
Crya  YLYLASIPLILILTIIILFIST-KFSN--SLYYIYGFSG-------NKSYVLLLSVPLFVSYIVIFNVIWKIILYKFYPNPSTIVRDWWLIH---------------------------------------------IKQYTDCCKNREKAMICISFMNFEYK
Pgig  TTNCWGKYYTLSFLTLNAGFFFVLMSLPR-----------------ITSVTSAVLLLGAVVVIFTFIWIADSGASALILNQAEFLYSWETYV-----------------------------------------------------------------------
Vbra  WTWLFTVVTVISVVFFFP-FLAVYAAIGWPEPQMGGMAE----AVFGSSTFWFAAILSCGTCFLIDFATYYV-RRYLFPSPVDLLDTWLFRS-----------------------------------------------------------------------
Evor  WMDWQILISIISVMLFPPILFIYSNPSMSNLLNPSFNDV--ATNLFESIPGWLSLLLVLALSVLVELAMDA-WRRVNRPDLTTRVQEFQRNK-----------------------------------------------------------------------
Pche  TTAAFLLCCILSVVAFAPLLSVYIYVRRPMVDASNLA----QLLATGYALTWILLLFSLGVGVVSGMASRS---CIFCWIPATPVVKLQRWL-----------------------------------------------------------------------
Tpyr  --WKLWVIGHISLSFLQLNRLWDINVRS------------------VSFVVTNMSLVCSIFLSFTLSLNFLIFMPLFQQKIKKASQQFYDFF-----------------------------------------------------------------------
Bsto  WTWIFVLIASLSLLLFYPFVFLYDVSAF------PTNNLVGVPSRLYKVAHWFFYITLVPLGCFIINLSYFFTRVLWYPTISDLLIKTSQ-------------------------------------------------------------------------
Pram  WTRAHYISLFGGFVTYILFVGAYSQAYRMFPTFDVFRDFQGLAGILFSETLFWTLLFFTSVTCICVDLAIMYLRKMYLPSSNDIILEIDSGL-----------------------------------------------------------------------
Ainv  WTRYHYMAILGGPILYLAFVLAYSEAYGIL-RIQTFNDFFGLGSVVFGGTAFWITICLVGFTSIVADVVVMYISRMYLPTNQDIIEEIDSHL-----------------------------------------------------------------------
Croe  WCFATLGSVLLSFLLWFAFVIVYSVLYTATGLSATRDFSSVALVLMQQPLT-WLGLLLLGTALALELATHHLLRQVWPTSLAILQEWDKGL-----------------------------------------------------------------------
2o                                                                          hhhhhhhhhh

PvivA SSKGFNKGGSSKFGQDIFDGEDISLGCNDDTQFDEEKTYGSARVTLCQQAVHGMENLSLSEGGSEKWDARGETTKGEEEKKRRKKKPGGRKKTRWGSPNGENDEGRQEKKASKATEQRDKPRLESRPRKEHPTGDDLPKHGGEQNGDTAPSIKEVAMKARKGKL
PfalB ------------------------------------------------------------------------------------------------------------------------------------------------------------------
Bdiv  ------------------------------------------------------------------------------------------------------------------------------------------------------------------
Tpar  ------------------------------------------------------------------------------------------------------------------------------------------------------------------
Esti  -----------------------------------------------------------------------------------------------------------------------------------------------------QAEEDRRRRVTGL
Crya  KLFKEIVSYMKTLIWSEWIIIKNHINIGITDNGDEYMDYLTIQNIKVPTLINSISSKYQIHSMERIKKRKESLKRQNVVIQPQINIIQPQIMNKSILENKIEQNSYYYDLNNSEGKTLPKDCIIEVKETVTTPVDERGVTEEGVSNNTEINTNTLNAGFSESNI
Pgig  ---------------------------------------------------------------------------------------------------------------------------------------------------------LILVSWGSL
Vbra  ------------------------------------------------------------------------------------------------------------------------------------------------------------------
Evor  ----------------------------------------------------------------------TTFAGESFAAARPTAFSTMTPIHPASSPSVDHARLGTQNSRSFGTDAKLTTVIPKVPGVERSPCANVLTQTGQELQLQSGISKTERLYPESL
Pche  ------------------------------------------------------------------------------------------------------------------------------------------------------------------
Tpyr  ------------------------------------------------------------------------------------------------------------------------------------------------------------------
Bsto  ------------------------------------------------------------------------------------------------------------------------------------------------------------------
Pram  ------------------------------------------------------------------------------------------------------------------------------------------------------------------
Ainv  ------------------------------------------------------------------------------------------------------------------------------------------------------------------
Croe  -----------------GTPESGDLVARAAEAHRSLAIAKERSRALKKVGGGYMLLQQSKWYATEQMEAIWDVEAMGDAWRPSSRKARASTRPGKHRHVIIGRLQGASNSGAAEMSVTGGKVLSMEEHLRQVAAIERLTSKVVNSQRLLWNALHKRWTRRRDP
```

```
PvivA AADEVTLPPVKGKEANRVAASGGKNGAANGEAANGEAANGEAANGERANGELPNDETANSELPPCEDIREDRTHSASDEYAESSISRIDEALMVEMKNCLNPLRNTFLVDLLPQGKRFRINEEHIFFKNNERMENIPEPLDVNKRNYHIIQNQAEFYDNESTSE
PfalB --------------------------------------------------HNNDKFLSQRKIKGINKFVNFEKNDILLKYIPTPKIYKIKDDPTYYNKSKRSKFLYDTFRKVIDINV-----------------------------------------------
Bdiv  ------CSQHKPADSVATNKTQRRSIVAMVQSIFAKMQNPHIWCDRLLKTLRKELGVHGAGALPMLMPAARPFRVREGYLPGIQEFGMTSSSGFSKGNVGNDMRKVYGNVSSQEKRGSHLIDRLTLHFKDA---------------------------------
Tpar  -------------------------------------------------------INFKNIKN---IFNFKNINIKNLMPVSRPFKVKNIHGVTSKFECGVTENNINKLINIYTLRFKDI--------------------------------------------
Esti  PLDEPRLRLLPGGQDEYGPYCGRRSFLSAIKRVLWVCGFGTFLPAPGPIHQLADRHILAYLRDKFNPLRHSEVSSKLPPPRRFRVNENFVTYGSDKKDKNLGTTGQHWSSARSLTNADDVSMDSNSSGMSFDGDKGRDQVKLVKVSHLINRFSLKFKDM-----
Crya  NSILESSCNSVGNLTIATSCTGNYLHYSNSLYLRKNKFAKPIKINYIGDNRIFIDKYENNINNWKLRVYMHAITIWNPKQIYISHLVPVKKTRVKGIKSLQFVQSLTKYSKEEQSNYIYLKKEYTDILNDNIRVGNIALQNNNLEEDKSGNNKCIDSSNVSKIV
Pgig  LMFTRFFRGGIWKLSALLRKAYDPGATIPDNLCFKPPKMHLDLPDGRTVLWGRKPARSFSHETPDDSVLNSDISEPPPEEMPEETTVAFSGASEALIHPLTLRFRDTHLECAYETSKNDWILPHRRRL------------------------------------
Vbra  ---------------------------RGHYVDDEATLEEVCYRAEVDANPHRNIYRRIVMKDHHKRPDRPSLDSEDPKNPASYVDSKLSIFWLTFHSCLRALRLSVLIISAVILIYALL--------------------------------------------
Evor  VTSLPYSYKRSGYNQGDFRAEAMELKEFAQDSKHPPIKQPGLSLIMEGLVRNRDGSIGLAHLGRWGEEWRARLSYLQWSQISLMSDVLGGKDANHVEEDVTQNIAEKLSQITGMQRELLHLDHLEPRDEETTTGPQAAPVDYRQSRFNVNYTTEFVYNIFTLK
Pche  ---ATVPHAWTRRGVAHIVQEFKERYHQATSSPANSGASS--LDVVARAQSDDDDEYLFSWTR---RIPVRKIVSNTLRFRRTVLRRSRFVSEGSLTTNEIAAQQTMMQVNPRTLEFKNL--------------------------------------------
Tpyr  -------------------------------------------------------DQFKQLVQD---KQPKKSSSSLTKVIRQVFQNRDMERALEELNTDDISISQSKTH------------------------------------------------------
Bsto  ------------------------------------------------------------------IAPEERYAENEPSQQKNNFRLEEYCNALNKVFINKNFKNLTNQDEDSDFYAMNS--------------------------------------------
Pram  GDLPVLPVRNADGSMDNSRASKLVYSSSGRSDEGSYTGG--ISAVGGSARSDKGELTSSLLEGEWSQQFRDFDLPTEHDELSKQLVSVQRELAQENGHPDPLMQEDDAHSSRALRVQPPVHPLTL---------------------------------------
Ainv  ---------------------------------------DDPTDEIPVHPKSEDMTQRKIQSPGVGAIRKDLGFLTEHEELCRKLMRLERAISREVGVHRQELFDVGISSTSEISLHPITV-------------------------------------------
Croe  KRPHHRSSRDAAHRTAAVAPAAAAPVPISSGPVPQAPQLPSSGQAARSSDGMKLSSFHASDEPAASHVQRSSRGLTVPAFVARQRLAFATPAAARDSTVSRASDQRRTSAIEQSDGYTHPQGSGTTLSHRPGQRQKRGSVVLAPAWTAGSEGQVLARASRAGGA

                                                                    |->      M1       <-|                     |->    M2  <-|                    |->      M2     <-|
PvivA FSYTSADSLNDSSGKKKKKKEDAQKEIVKVSNLINRFT--LAFKDMQLESGFQIHKKNKF-YKIFTPWYRFIFILLGVFFLYIWKLESSLSRYWNISSNASTDLFILILSLLLELVLLAATVTTFFQNIFIENFNKIISSVILIITHHVVTYSITHI-------
PfalB ----------------------------------------KYRNQQLNLEYKTYEKGNKLKLRIIVILLFLIYIIIFSSQTIIDI-----------NTKSNIHYITMFYIIYFVLACVLLIYIRIRNK-----ATSTFFFFLSRFLLICGFCIELYDNISN---
Bdiv  ----------------------------------------QLESDYTRDKRRNHYRMNRVWYRSLFLVIGAFYIGSWILDYYLKK-----------AWNLEATKYSFITPLFVTILCVCCALTFHPVYFVNHVNKALGALVISMILHHIIALRLVKPLSS----
Tpar  ----------------------------------------QLESDYRLDKNKKHYYENGHWYKIIFILLFLYYLSSFVVEYLIQK-----------RWFKAPVIYCVIPTVLLEIVLVICIIISFNHRLFIRFINHILYSLVTFMIVQHVSNRFLLRSISW---
Esti  ----------------------------------------QLEADYQIHQKKSFVKRLVPWYRVIFV-FLGLYRIVTFLAEYFIDHYWGSG--------DHVFVWMCIPTVIVVLGLIGATISTFS-HFFMDHFSVVLSSVVFLLLAEHVTLYAAVRMDGI---
Crya  NSLTSITSLNRNIKSSTMNKKKQDSTLD------------LNINIINNSSDYSRINKFSDDVKLLLNPYKLTFKVKQLELEYQREKQI-------------ETYQQRYAIRVVLFVLSLIFLKLGPFAINLSDTNYSWVGIMCVLFCTFIFINFCLTYISGKYS
Pgig  ----------------------------------------VFMLVVAGIMIVLTSGYGVLAMCMALNSGFSSRTTVCGMVSSSIVSDLCRT--FSLVEVCGQCLSTSVVVVVMLPCICPVLLLIGST--------MDFMWRSEALFEIYVVTYFFLRQVTFFFM
Vbra  ----------------------------------------HFIYFYEEG-EQDFDDCHSDDRRLLTANGTLQAGNQGKTRVRLGEDYD-----ESHGDDSQDVLSLGRVWLLFVPLLGPAAYSLTFS--------RFMQRYCAHVMVAVVVVADFVKMFMDAVQ
Evor  FRSK------------------------------------FHEQAFCQNFDHTSRNQFFVSVRSVTWVIIVGFQLVKLIALLAAKD----------------SESIPQTLLIIFGAVAASEIILFPL------RFKIMDSIQASYVAIICCSLMIAKHAYEIVA
Pche  ----------------------------------------ATESRFVIHAHTEAVKQMQAFIRCIILPVFVLWLLQDLVQDSLT-----------------IGRLAVQLFILISGGIIYRATKTYFY-------CRHYTGITIFTALVPILIFYIRRSVTEMDD
Tpyr  -----------------------------------------LFTLKFLQLDTENKYIAMISLKDKRFMQISLIVQILLQELAQALIYSY------------ELKDNPNFNSLSTVSAILTIIWFLTLTS------LYTKYFIQINNLILIVRIISRMNMILTSR
Bsto  ---------------------------------------ITLAFKNPYVENSYKQSMLDKQVRFTRWMIKIIFVLYSIYCIADLSSNSY--------------GDAYIAFRCIMIGVVFSLSFFASS------QIFKKHYELFMVIIIFCALLIKFIFETIAMN
Pram  --------------------------------------EFMGEEHQRLESEFEITFAFRERSRVVLCLKVMAFMIPLYAAYEVLW----------------EHESSYIWIRVGYFVCDLLFMRFARTKFF-------VQNYQLSILLPYSIVGMALTLTI-SDT
Ainv  --------------------------------------EFMGEDHEVLEAGYNKVFMAREVRRIHVLVAMFLFLLPFYAVVEYYW----------------EKDRTRYWTRIGMFCATMMYLGFLHSRFF-------LSHYQLGILVPMAIGGIAFTQAI-AYT
Croe  QLRQHFREMQQAKASGAEGLALSLEDSAAPRVTRFTRQFADDPELERAYHFRFFLGKSLPVTRTGLLMASALGGAFIIYEYFASVAGFGG-----FARRPPAKALTTLLLRMALVVGALLAVLFTYTTTF--------RRNHNSIMFALLLITGIAKTVIVSDA

                               |->     M4   <-| |->    M5    <-|                                      |->       M6     <-||->          HD1        <-||->  CTE1  <-||-> C1
PvivA ----------------------DGVFQAVLFPLYTFVILRLPFVNAVLCNIIFLSLFII-------------------------RFKGDHFLDKKGLAHYIPLFIGVDVFVGFVGYRLEYNQRKNFLLEYSVESSRRKQREILNTMLPPFVVDEMIYSELNEEG
PfalB ------------------DILNVLITYSFTVSYIFFMSFKILEALLVCISILLLTFGVYYEKNKNMIDICTHFCSNPYLSINNLDHMNISCLCKKQIVIFLISLLSFTLICLSMKYYEIFYLKKKFLFRYKQKVNLAKQIEILHTMLPNFLVEYLLISDPKNDG
Bdiv  -------------------------LQPILFPIFTFVILNITFIHALLANIVFVIVTV---------------------------------TNVDTSIHTVPLFVGINVFVAFVGYRLEYNTRKNFLFEFSAQIARKKQSELLNTMLPNFVVSKMINARLNEDG
Tpar  -------------------------LHTVLFPIFTFVILRIPFIISVGLNLIFLINTL--------------------------------ISINSTNIHILPLFFGINIFVGFVGYDLEYKSRSNFIMEFSVLNYRQKQSELLNTMLPKIVVSKMINAKLNENG
Esti  -------------------------LSSVLFPVFTFVILRISFLQAVMWNILFVAAFT----------------------VRYMVGESYNYLSLSDLYDHLPLFLGSIVFVGFVGYRLEYNQRKSFLLDYSVEASRRKQREILNTMLPPFVVDQMINAPLNEDG
Crya  LNVRYQFLLLILISVTSILATIHGFTLVVIFPILSFIVFRLGFIKGLYLTIIGFVCVIIVQF--------------------------IWGIKSTYLLRFLPILIGINSFCGFLGYRSELLYRTQFLLKTYTNDLRERQRQILDTMLPSFIVERLIKTQQIDNN
Pgig  LDPGISDVPIYCIIVFILIRVPFKRAVILFAVYGSVNLARYIWASSYYDACMDNLVHKDALR--NYCPSFIVADLISTGSYLCDYSLMFHKMSASNLFYYIPLMVGVSFFTSLVGFQVELHQRKAFLLNVQADTIQRQQKEILTSLLPDFVVKKMIESAFNMSG
Vbra  RTDGVQFAAMYPIGTFVVFRIPFKHAVWINALHLFLFSVRYTAGAWIFDVEVPTHSQDLPKM------------------------VMFRR-NLYTMCDYWPVLFGITFFTAFVGYRLEYYQRSEFLLDQQFARERRRQKDILNNMLPSFVVDKIVHTKGDW--
Evor  --------------------DVPGYVSDAIMPLFFCAGVRMRPFVVFVFLHMVLLVLHQ----------RLFLTEVTCTVGNGNTASSVCDQNGGLKAEYSVVQFGLVLLVGTYVYVSERFARHSFAWDDRIGSARNADRDLLKGMYPEDVVEAVRTSFRFNQN
Pche  ------------------------AHMVPCIPFLIFIVIRIPFPKALAISSVHCIFFICLHLL-----RRVNIHRGDGIEIIYSRSCGVDLNSFCQLMDYLPMVLGLTAISATIGYQTELDLRSHYLVSMNSSLEQARSKEVLNNMLPTFVVKSIIENNQDIVP
Tpyr  FIM--------------------NSFEEIVNNIILSQSSYINPIYFSISLVTTTVTYLITFFV------NYSDKEYFYFYDNHHDSYFASQTNNYIKSIVLVFQVSITILIIKNKYRFDQLQRLHFQKENHLSYETKKIQSILSILMPKFIRDKIDKDGEVQ--
Bsto  D-------------------ASMSTAIVPIAVFILFNVSNYKMFIINLLFLVMYLIRITIW----------------------YGGHSSQAEATIIITNYIALLSGIFLISAYVGYKIEKFNRISFILDKKLQFQFQKGQDILGNLLPGFVKDRV-KQGVRY--
Pram  -----------------------GKFAAALYPVGLFVVIRVKFLYALMLSLYNFAFYMLAD--------------------RLPSLDYADAVSVSDLMLFALYLVFVITFGSYACYSLQITMRRDFLQSRSLLIEQKRSTQILKNMLPEHVVQRMQK-GDTL--
Ainv  -----------------------GLLLVTIFTILLFSVVRVKVVYAICLAVFNLLYFDLSP------------------RLGFRQDTTSSETTATEVVLFLVFMMFLIGCSAYGSYTLQVSMRTDFVQNRILVYEERRSREILSNMLPEHIVRRMQN-GEKL--
Croe  -----------------------GTFGQTLYTICVLLILRLSFASAAVLAILDLAIFTA------------------------AVGANLVGDGVASLIDYIPYAAFVLAFGLSGALAIDEAMRQDFLQQARLAAEKKSFNDILKSSMPPHVTKKLKRMELGGFR
70%                                          R                                                                            Yζ E   RζζFL          +ζζ-ILζζΨLP  ψVζζψ
cons                                                                                                                                                         L   LP
```

```
PvivA IPIS------------------------------------------LKAEDIETVTIIFCDIYDFQNIVASIEPTRLVEVLDRLFLCFDKCTEQFNCTKIETVFETYLAACGLV-KKEG---------------------------------------SESKEN
PfalB IMVGKN----------------------------------------ISGEDRGIISVIFCDIDDFQNMVSTLQPHVLVETLDNLYLYFDKCIKYFNCIKIETVFESYLAASGLSEKKNNALD------------------------------------------
Bdiv  IPIG------------------------------------------FEAEEHESVSVIFCDVYHFQDLVATVEPTILVEVLDSLFLAFDRCAEQFGTTKIETVFETYLAASGLS---------------------------------------------RDATS
Tpar  IPIG------------------------------------------FEAERHENVTVIFADVYNFQELIATIEPHRLVEILDTLFLSFDRCSKEFNAIKIETVSETYLTSMGLNTSSTSQTKSLGMTDGHDHDQSRNKKSGSSLGRRSRRSKDIAIDTAVNKLD
Esti  IPTS------------------------------------------LEAEDRGTVSVIFCDVYEFQQVVASIEPTRLVEVLDSLFLCFDKSAEQFGCTKIETVFETYLAAAGLQP------------------------------------------GKGGESD
Crya  IHSPYGRSSNSLKNSLNDKLEQQCDNNYSRKTRISYQISKSVNYYKNGVEDRGIVSVLFCDIYDFQSIVEVLQPTKLVYLLDKFFLTLDCLIDKYNCTKIETVFETYLIASCLDPYNSEDEYEDKEK-----------------------GEKSRKSTKDNPEN
Pgig  -------------------------------------------MGFFPAEDRGKVSVIFVDVYEFQALVAGLDAASLVRVLDQLFLVFDGLSRLHKCAKVETVFETYMAAAGLHE------------------------------------------------P
Vbra  -----------------------------------------KNERKNVAQEWQAVSVVFCDIYDFISIVAAVEPITLVTILDRLFTHFDRLSDRFRVAKIETVSETYLAAAGLNT---------------------------------------------EEGG
Evor  QS-------------------------------KDTQIASVSS-TETIFQERGVVTVVFIDIYDFERVVAHLGPGDLVAMLDRVFTQMDLVCQEHDVVKIETVGKTFMAA-GMTGDKKKHGKLGRS---------------------------------GNGKD
Pche  LPEPKSRPQL---------------TRSRRTALGDNSKSGSVLEIGTFARHCGDATVLFCDIAGFPQLVAVLDPKQLITMMHTLFSSIDRLVFIHALTKLETVSESYVVCSGLNPDTNASEKEV-----------------------------------STDAF
Tpyr  -----------------------------------------------LQEDQGDVSILFCDICDFEQILST-EQENIVRTVDKLFRHFDGLCTHTGLQKIETVGKTYMAAGGLKDAEGKKSQ-------------------------------------FRQIN
Bsto  -----------------------------------------------IAEEQAQITCLFCDIYDFDTICAIHTPNELLELLDKFFAMLDALCDKHGVTKIETVNKTYMICGGLKDSEAHLPKK------------------------------------QLRRN
Pram  -----------------------------------------------ISEDEQDVTILFCDIADFASFVNRFSPTEVVSLLDRVYSLFDQICQKHGVRKMETVGKTYMACAGL-------------------------------------------QGKDQGRE
Ainv  -----------------------------------------------ISEEEKDVTILFCDIGDFSSLIKRYSPTEMVKLLDRIYSLFDVMCAKHGMRKMETVGKTYLACAGL-------------------------------------------QGSVKGKE
Croe  ---------------------------------------------GKYYDKEEEISVIFIDVIDFEKVTSSHTPQMMVTLLDRLWRLFDALVEKHLVTKMETVGKTYMACAGL-------------------------------------------Q-KSRSDH
70%                                                    E-ζ  VTVψFCDψ DFζζψψ    PζζLVζψLDζψΩ  FD        ζKψETV ζTYψA  GLπ
cons                                                            F D   F  L     P  VV LLN L               VETIG  YM   GL
2o                                                    bbbbbbbbbbbbb  hhhhhhhhh  hhhhhhhhhhhhhhhhhhhhh  bbbbbb  bbbbbbb
                                                             b1         h1a              h1b             b2       b3

PvivA KGQQDAHDSIDFALSILQVSSHIKYERTKHMFKRGDSCGSGAEGSQEAAKKDVITSGGANAATNANAANIANTANAANSANGGEGRPARIRVKIGINSGRIIAGVVGSKKPQYALFGDTVNTASRMKTTGK-PDYIHISEATYDLVKDDKTLVYEKKETEIKGK
PfalB KIMYDTKCAIKLAIAQLSAKYYISYKVLDTREH----------------------------------------FSDNSTSYD-KYINKNISLKIGIHTGKAISGVIGSVKPQYALFGDTVNTASRMKSTSL-PDHIHVSYDTYKYLKEDNTFIWKERKVFIKGK
Bdiv  SPSESAANAIDMALAMIEAARQVRYRSRTEKEDG-----------------------------------------------SYIEEHPSLPVKIGINSGRVISGLVGAKKPQYALFGDTVNTASRMKSTGE-VGYIHITDSTYNLVKADTTLHFSHRETDVKGK
Tpar  AYVQAASSSLDMSIAMLQVATTIK-----------------------------------------------------------LNTNESIGVKIGINSGYVISGLVGSKKPQYALFGDTVNTASRMKMTGE-VGRIHISESTYELVKGDKTLEFINREIAVKGK
Esti  TSRTDAGDAMDMALAMLEVAAQIRYEVTRDLTTTGGEDEQVSPAARTSSS-------------------GMVRQKTATLRTVLSSRPQRIRVKIGINSGRVISGVVGAKKPQYALFGDTVNTASRMKTTGQ-PDYIHISESTYELVKDDTTLFYEPRKTQVKGK
Crya  NEKIKKKSLLDLINLLRFGLRMHKISSTIFFEIPINSNNGTDNDSNIDANTNSKNYFN-----------------GDITIMENELCLKRLKLKIGIHSGKVISGVVGKNKPQYALFGDTVNTASRMKSLCD-SGKIQISSSTYEIIKSIPILKWRERRSFVKGK
Pgig  HEAHMHSVKDDALDALRFSVAAVFGANTVEYEARSEDDVAPSGLLSSHESKRNQMSN----------------------SLAGGRRKYTMRVKVGIHTGRVISGVVGAKKPQYVLVGDTVNTASRMKTLGE-VSTVHISADTHQLVAPEKTFIWEERQCFVKGK
Vbra  ESVTSEEAARDAVSALKLAIAMLDSTSKMLVDLKKQTQGG----------------------------------------DGATTERRPLQVKIGIHTGRVISGVVGSRKPQYALFGDTVNTASRMKSTGE-PQHVHISDATYALVQDNSHFVWEPRKTQVKGK
Evor  SVQKKAVKCLEVAVAILRQFSKCATGMNG---------------------------------------------------------MERLSLKIGINTGQVISGVVGSQKPQFALFGDTVNTASRMMSTGK-VNHIHISKQTYDYVEDDDRFKWTQQQIEAKGK
Pche  RAVLLGLDILENTAYTNVLGGPAVDGVSEET-------------------------------------------------------SIALVMKIGIHTGPVISGVVGSKRPQFCIFGDTINTASRMKSTAN-PGRVHISANTRELLELSSSLHFNQRATSVKGK
Tpyr  PVARAVIMSFEMMEAMKSFTYGD---------------------------------------------------------------NEKIKIKIGIHYGRVIAGVIGCHKPQFSLIGDTVNMASRVCSTGD-VGQVTISDEAYEEIKQVPHINFLKKKVYAKGK
Bsto  HAERCLDIALHIIKKLEPVYIKN---------------------------------------------------------------GHKLQVKIGINSGPVIAGVVGEHKPQFSLVGDTINTAARMCGTIKVPDRIQISEDTYNLLNNHNEYIFLPNKVEAKGK
Pram  AALRAVSMALDMLTCLD-KCQASNG--------------------------------------------------------------NGIKLRIGVHSGRVVSGLVGMKKQQFSLFGDTVNTASRMQSTG-ITGRCQISQVTYKKLAGYFAFEE--RQIDVKGK
Ainv  AALRAAGVAHDMMAAIS-KVKASNG--------------------------------------------------------------NGLKIRIGIHSGRVISGLVGMKKQQFSLFGDTINTASRMQSTG-VTGRIQVSQVTYDYLCQDFRFEP--RTVEAKGK
Croe  AAALVMLGLDILHNLRSFKDK--DG-------------------------------------------------------------RRIISVKIGIHSGPVVSGVAGLKKQQFSLFGDTVNTSSRMQSSGE-PNRIHISPATYALVEGLFETEA--RRTPVKSK
70%            ψ-ψ                                                                            ζψζψKIGIζSGζVISGVVG ζKPQYπLFGDTVNTASRMζSTG     IHIS  TYζϕψζζ     Ω  Rζ  ΦKGK
cons                                                                                              RIGI SG VVAGVVG K  RY LFGDTVNTASRMES G     IHVS  T   L       F  R    KG
2o     hhhhhhhhhhhhhhhhhhhh                                                                      bbbbbbbbbbbbbb        bbb hhhhhhhhhhhh     bbb  hhhhhhh        bbbbbbb
               h2                                                                                     b4                b5     h3            b6     h4             b7*
```

```
        C1    <-||->        C1b
PvivA GTMTTYLLTSVIGLNYPF------------------------------------------------------------------------------------------------LEEPTEGKDHFLPDFYLDSNDPSNNINVDQTHNILGFYSNK---------
PfalB GKMKTYLLVDILDDVKRKGESLNYYSSSNLLLSQLGSEAVSIYEEREDIKEGSMDIIKESSRDIIKEDSRDIIKEISTNISKSSSRNISKSSSRSISDIKEGQIIDKEDLIFKINRMKNKIDSRYSKRIDKESRDKISDKTNHVLDEVVKHSDIHLLNYEINNK
Bdiv  GLMTTHLLIAAGGSAYPTFEVGDTTNALCTFRTLNSSDELLQSSESIEPFADSVINIHSKDNNLNANGSMTLFECLEKEHPLNSTECGGFQAES----------------------------------------------------------------------
Tpar  GVMNTYLLEKALGTSYPNFSNSNNNLHEVDIYYNMLNKNYSASAYGDGTSSSGDTLENIPLSSRELNVPEVSLTD-----------------------------------------------------------------------------------------
Esti  GDMNTYLLVRVLSSSYPNFSEEERSLSGPLGSKDGAGMPPFAQNSATCAPDGEAPAAESLLRQAVFTSEGEHALESPGSLLPSTPSTPRLTPGATSRSSLSEALSPPGGSSPRTPSIYSLDRAPSRSQRSYSPMSSGESAA-----------------------
Crya  GIMDTYILERVTGSAYPNFTLIAKGRIRGKGVNSVQVGNCIVKLYPDFDLLNEYNNSDYTVSQLSLLPSLLKNNVTNDKNKYNIIKYTK---------------------------------------------------------------------------
Pgig  GYMNTYLLLRVLEQPPDSLNSEEDIESYSLSSAIKETDED----------------------------------------------------------------------------------------------------------------------------
Vbra  GIMDTYLLVDYDQEHLLGAVMRGLPGQTRMAKGSLSQAQQGGVALDSAIPEKANALQKNLLKMMKSADNVEETRDTSMTPNTSTAPAPPAITYTNPPGGQQPGAS-----------------------------------------------------------
Evor  GIMTTYLLWNE--KRQNENGHVVRQKSHREGSILELASSDVVPSTEETTWH-----------------------------------------------------------------------------------------------------------------
Pche  GMMITYDVTRVIGSPHSMDAGVMSPRASARARASGDTAEKTGRVEPFRRPSQASTGASSIRSFSAPVKNLVTVLKRFSVERDRTEGLSGSANLAPAPPSQHKLRGGVPMGLDKSRDLMQAVETLRRHSGNQGII------------------------------
Tpyr  GDITTYQVTKKQKQKNQNSSDIPLRDQVIQRQSQKKHSENGFKNQLAEINTKNLRKNKTTILKHKKFLNDSHSEENFEYFNSFNFQLVNPQKQDLTKSENKKIMSKTLPVSESQSVIKVLSDEGTTQVDDQSTIKFTLQNNQQHNLNVVHSSKGSYTTIFSKVM
Bsto  GIMDTFLVESAKGRKQPRMLGEGGIRI------------------------------------------------------PINDLSGEESFYGISDSVLEKSSEPLMQDIIRGTTILNFEEERVQ--------------------------------------
Pram  GSMTTYLVGEPLTEVAQMAVLGQLEFF--------------------------STKRESIMMSSSVINVRRPFEQDFLAITRNKILHSARQRWKKYDPIRMLLGTPPDLASYGG--------------------------------------------------
Ainv  GTLTAYLMGKSATVLGDRALRGHWEPY--------------------------VDTKAMASPRSASRGALELMIMHNMREWVRRLFAPTNRIQVVTSNLDTTEMSMPS-RMRIQ--------------------------------------------------
Croe  GIMITHLVQRVANSDEAMKRRRASALGLAPLLAGPSSQATGAAARAAATRAAALAVAQASPGAAASGRQIVAVAEAEPARPSARLSRHGSKTSRSESPGQLAKRTPSRTSLPMGVQRMAITEGKLDSAGDLAGPSEPHLEHVDSPSAADAETPRA---------
70%   G M TYLψζζ
cons    M  Y L
2o    bbbbbbbb
         b8*


PvivA ----------------------------VNQNNEYYINDTIYNDEQ-SFNESDNDMSDAYVHLKELSLQD-FPNEYI---------------------------------------------------------------------------------------
PfalB RCKKMKGDTNNENKLIGDIFNMYDKKIKYIYKKNY-KSKSMENISFIKHYRNTKYKKSDYLLLDNKGESKKFKRNTSYVLESPLHLIGDIVDNNIKRKKKKKEIKTIVSDDMFTSPVNIKEYNYNEQERKKEIVGNLSYDKTKKIFPFIKFTKEGRIKKKKIEK
Bdiv  --------------------------------------------------------------------------------------------------------------------------------------------------------------------
Tpar  --------------------------------------------------------------------------------------------------------------------------------------------------------------------
Esti  --------------------------------------------------------------------------------------------------------------------------------------------------------------------
Crya  --------------------------------------------------------------------------------------------------------------------------------------------------------------------
Pgig  --------------------------------------------------------------------------------------------------------------------------------------------------------------------
Vbra  --------------------------------------------------------------------------------------------------------------------------------------------------------------------
Evor  --------------------------------------------------------------------------------------------------------------------------------------------------------------------
Pche  --------------------------------------------------------------------------------------------------------------------------------------------------------------------
Tpyr  FSNGIIQKPFSSKVLGQQGEAIQEADEEEDKKIDSVNKNQIRISDRKRTDSNSQAYERSQYRHKTTSLNNKITTRTVNSPSIEQQNNIESSVRDSTEFLKRITNDSRIRENSNHTDVYAGESIPKISKFRNESEQIMEMDPKNKPNYYYTLNSLSPDRKRNKLT
Bsto  --------------------------------------------------------------------------------------------------------------------------------------------------------------------
Pram  --------------------------------------------------------------------------------------------------------------------------------------------------------------------
Ainv  --------------------------------------------------------------------------------------------------------------------------------------------------------------------
Croe  --------------------------------------------------------------------------------------------------------------------------------------------------------------------


PvivA ----------------RDVNSHFYEII-KLRNLKIGTAVGHQLK-QKDVLNMTFLNNGLNNDTTTNFNPYIESNKNFEEVKEFDGGMNKLSLSDSLYCLNENFIKKD---------------------------------------------------------
PfalB KEKKEKKENNNNFLYNDDYSSYSSPKYGDNENNFVIKYIRERKDFQKKFDHPNFNFSKFLHNYNPMKNKNKNKKNNKNVRRNEYPNYTSSSKDGVSYNFLSDSLFSSDNEYSSDNEYSSDSEKYYKKRFKKNKKIIKFDDLFTKIYIKKKRLLQMNNYDVKGKG
Bdiv  --------------------------------------------------------------------------------------------------------------------------------------------------------------------
Tpar  --------------------------------------------------------------------------------------------------------------------------------------------------------------------
Esti  --------------------------------------------------------------------------------------------------------------------------------------------------------------------
Crya  --------------------------------------------------------------------------------------------------------------------------------------------------------------------
Pgig  --------------------------------------------------------------------------------------------------------------------------------------------------------------------
Vbra  --------------------------------------------------------------------------------------------------------------------------------------------------------------------
Evor  --------------------------------------------------------------------------------------------------------------------------------------------------------------------
Pche  --------------------------------------------------------------------------------------------------------------------------------------------------------------------
Tpyr  FEDSQTEVALGTECQLNDTQFIRKNKRYLSKELSKMWSQKVENPESSTIIIPPILALEQELDIPLKIDIVQEV-------------------------------------------------------------------------------------------
Bsto  --------------------------------------------------------------------------------------------------------------------------------------------------------------------
Pram  --------------------------------------------------------------------------------------------------------------------------------------------------------------------
Ainv  --------------------------------------------------------------------------------------------------------------------------------------------------------------------
Croe  --------------------------------------------------------------------------------------------------------------------------------------------------------------------
```

```
PvivA --------------------------------------------------------------------------------------------------------------------------------------------------------------------
PfalB KKLKNKGMERNKTKYKNVNEITKMKYFVNNENRDHEVNKEDISKSMQKYFLHISKHKKEQIEDKKKTHKYFHKNVECVYPYAGNNINHNFSRNEKRKYSINLYDHLDEQEKIKGKKKYFNKDKELIGSINKQTERKPKKKNKKNIENKKDKKKIRMITNKTKEK
Bdiv  --------------------------------------------------------------------------------------------------------------------------------------------------------------------
Tpar  --------------------------------------------------------------------------------------------------------------------------------------------------------------------
Esti  --------------------------------------------------------------------------------------------------------------------------------------------------------------------
Crya  --------------------------------------------------------------------------------------------------------------------------------------------------------------------
Pgig  --------------------------------------------------------------------------------------------------------------------------------------------------------------------
Vbra  --------------------------------------------------------------------------------------------------------------------------------------------------------------------
Evor  --------------------------------------------------------------------------------------------------------------------------------------------------------------------
Pche  --------------------------------------------------------------------------------------------------------------------------------------------------------------------
Tpyr  --------------------------------------------------------------------------------------------------------------------------------------------------------------------
Bsto  --------------------------------------------------------------------------------------------------------------------------------------------------------------------
Pram  --------------------------------------------------------------------------------------------------------------------------------------------------------------------
Ainv  --------------------------------------------------------------------------------------------------------------------------------------------------------------------
Croe  --------------------------------------------------------------------------------------------------------------------------------------------------------------------

PvivA -----------------------------------------------------------------------------------------------------------------------PENDHLHHRSKLGTQEIIDQHTKQSLRNFSPSNRDLLNNDEDDYR
PfalB HSNSIISVEEQNMNHNNSLKKKEVNFTGKNEEYLNRANTNCSLGIKEMEEDVYEFHSNNIYYNNQTSYSDDINNTTKLKGMGNNTNDISKNKGKNKLGKKISFFSMNNKYHESEIMNEEDNKNMLNLTQSQIINKDKYNYFTHCPSLKKKKSVFTKINNLFKNY
Bdiv  --------------------------------------------------------------------------------------------------------------------------------------------------------------------
Tpar  --------------------------------------------------------------------------------------------------------------------------------------------------------------------
Esti  ----------------------------------------------------------------------------------------------------YSMTPQQLTRAYARQESVSRVLAFARDGLQMAVRESSDLKRQEIARQVHARILREQLHQGPSSV
Crya  --------------------------------------------------------------------------------------------------------------------------------------------------------------------
Pgig  --------------------------------------------------------------------------------------------------------------------------------------------------------------------
Vbra  --------------------------------------------------------------------------------------------------------------------------------------------------------------------
Evor  --------------------------------------------------------------------------------------------------------------------------------------------------------------------
Pche  --------------------------------------------------------------------------------------------------------------------------------------------------------------------
Tpyr  --------------------------------------------------------------------------------------------------------------------------------------------------------------------
Bsto  --------------------------------------------------------------------------------------------------------------------------------------------------------------------
Pram  --------------------------------------------------------------------------------------------------------------------------------------------------------------------
Ainv  --------------------------------------------------------------------------------------------------------------------------------------------------------------------
Croe  --------------------------------------------------------------------------------------------------------------------------------------------------------------------

PvivA DIQLCSRKCNICGENYCTPNECVNYDHVNHVAYHKMLRNIRRNYLKNIRKDAKKEADRKEELDGRGSKGGLLSPSHIASYLSKVFSIWGWRRWRKRKEGRSTQGEVNHLESPPKMEYAQGKPHLGNPNSRAERTPPAIEKEDKFKRVISLNSEWIFLKFSDKNL
PfalB FKSIDVHEKFGFSKKFKFHSKDSDDIKGNNNKISKNRYNNNNNNNNSNYSNIDSGKYSHNNKKNHHHNNNKYHHHNNNKYHHHNNNKYHHQNNNYEKHHHSNNSRVMLSKGEKTEKNENVDYAYQFDNYDKKLLKKLTSNLQLNKKNVK-NFNMFYYKFNDEEL
Bdiv  ----------------------------PCHISSSMGMLFSNNYDRARARKLRISAAVGALTEYDEGDEVRDQLPLLKNTIYSMGTSSSHSTPIRGHSRCISFFRRSNTITHTNSLIASSTLQSASFTKQITQMEDDTNDKLDGRDIRMQTREWLQLKFKDKLQ
Tpar  ------------------DELNRIKNRRTNINRAVGNEDDLYDNLYDYNTLNTSYSSNVTVGSHKRKCFKFKRHSSFMSKSTSMRLTRKFTSRLGTKHSSFSKNRFSVHSAKNTSIGSSSIVSSLSTPKEKK-RKSDPSHTLKEDEIHMRKNESILLKFNDKLQ
Esti  WSSQEDMSVGGQSLPAAVTSIPQFRYAASHNADLRNTSRIGRSISTSTRHSPRVPTKRTLSEAQGVDLRVSTPDMADSRFTSQASNAGCLGGKWRAANEVFNQIGTEAHAKFSVLLKALANSSKEDEHIIDLGGDDEDNFEAREARIGVSRESLLLMKFKDKNL
Crya  ----------------------------------------------------------------------------------------------------------------------------------TKVERRDRENDQVITKKRIANLMDVTLCFKDNNI
Pgig  --------------------------------------------------------------------------------------------------------------SISMERAMDQKQRRYCLASFIAILQRLFRSTADGTDKALQAKTQRLLNFQDRRQ
Vbra  ---------------------------------------------------------------------------------------------------------------GGGGEDRRRRSSKTVKGLPADTSGALDAGEEGRFMLDRKELSWFLVFHQKDRE
Evor  --------------------------------------------------------------------------------------------------------------------------------------------------ACPQLRLSNLPKAISDGW
Pche  ----------------------------------------------------------------------------------------------------------------------------------------KESSVGQTKLDWDKVNVVTLEFRHAADE
Tpyr  ---------------------------------------------------------------------------------------------------------------------------KEAEVEIEKLEELQGDEKFLLKNLKNKNEDSKKSIFWKYIN
Bsto  --------------------------------------------------------------------------------------------------------------------------EGEKFDWSKLRKVHGDEDNDLELAG----PMHWLMCSFFETP
Pram  ------------------------------------------------------------------------------------------------------------------------------------------TDASESVEQHMLTEIRLLNMCFKVEE
Ainv  -----------------------------------------------------------------------------------------------------------------------------------------------AIKTEMNMEMDLVWLFFLKPD
Croe  --------------------------------------------------------------------------------------------------------------------------------GEDGSVTAGTQPEAESRRRESRFSIVNWLYVFRDKE
```

```
                          |->      M7     <-|                  |->      M8       <-|                                                                         |->     M9
PvivA EAKYRAHFYSNKSNINSIEQALIIFLVTFVMQTLISSTVSIVFVDHKIATQTLEVNYFAYWSVRSLYTYLGFILWLLFHYRTRPE--------------------------------------------------------VSSLLNIKWMIFFLNLLFISAAC
PfalB EEEYTRNYYREIINIDLTKKLIIIFIFTEIFLSLCNIIELSFYEKKLRYND----SIVIIWLIRSIYLFIITYIWIILKTKLKEY--------------------------------------------------------KNNSSKMMWTIFILNIFLCSWGI
Bdiv  EDRYRSHFYHNRAHVNTIEQSLVIFLITFVTQSILEIAIPRSFVDNEMVERFLYLRYVPYWTVRSVYITLLFLMWLMFHFKSFSQ--------------------------------------------------------RSESNTKMWLTSFLNLIFVSAAC
Tpar  EDRYRNNFYNNVNNISINEYALFIFLITFIVQSIINVNVPRIYVQ-DNNKLIVYLNYILYWTVRCFFILMSFILWMLFHYNFFIQ-------------------------------------------------------NKEDNKKIKVCTFFVNLLFVSATC
Esti  EARYRTHFYNNKSNINTIEQAIIIFLVTFAVQTLVRLTLPWAYSEEVDGVKYTVRVSNELYWGVRATYTCVAFFLWILF-------------------------------------------------------HYRNRDEVASRLEIRWMVFLLNVLFISAAC
Crya  EQKYLLHYYSNYTTLKTIEESLVIVVVTLVLQTLIYSVLPCQSPKSDDR------SNYRILWTVRIVYISSILLSWLVLFSNYTSHQFRNTNYSTTERNVYDES-----------SSIMRNINFDIGNKNNKSNDSQKQMNYESERHIGGPILFLNAVLAGGAS
Pgig  EALFVQNFFLSDSNIRHVEYTLNIYVISVVVSSFAYLTVRRDGETE----------LFKVSQDKVLQIFSIILAVTFLELVLLHYRGTFFKSL-----------------------------------------------------HHLTWLFFFMNVTFLAVA
Vbra  MRYLDSILFNKYVFIN-MERALRIWIVAYLLQTLELRFIPQPHMALADTDKQECEPTYDRQIDYSMLLLGRSTLIVIFLSLWLTLRNRESPIWQGA-----------------------------------------------LTGRNSVRLIYAATFGYFTGA
Evor  HFCVKVYELDSGRTQQGGSMLGSLLLFWLCFGMESWFIIHGDTGQNASDMDLFLHIRGGFALVCLGVLVLFGAALSLLRFANPSEEKKPVLTAKSSADSLSEIASDSRPAAHAATINKSAETINKSAEQEEGITEEEEEIQHKRQTNVCLRIRQWLVYVVPIVV
Pche  STFRAQRLRGFIASTRSKTLLLILLITYLVHTIELAAVSQQPP---------------VLLPRVLGTRLVLSALLLVLYALYTAR----------------------------------------------------------GIVPASILRLATVGVYVLGTG
Tpyr  VDNELLKVFRVHQTDININIFRRSLFFCISSCLYKMCSVLLVVH----------------DFSNMLYSVIVLQGLAVALCIAPLIKRNYMCD------------------------------------------------------LSGTRLSIYMSAIYYIVL
Bsto  SQHKFRIRQFNNEIKSVNIGLYLSIGTFTAIIIVFIAAYIAD----------------EITGSVIQVILRLSYVVILIFYLILLLKMYQNP--------------------------------------------------------MFPWGVMMVYIYITFVN
Pram  MENKYLESTLSDRVEGARTTLLALAVYALFTCVRDLLVEIFSVLS------------TDAPQKEISPTMYAILLFIRSPFALVAILVARRVRDE-----------------------------------------------FVFYSEIKLRNVLLGFYLVGLLV
Ainv  LELAYRKTRCFIRLNGARRTMIMLAIYLAYTILRDLVHDASLGDYNFRVIHSNATMCDNKCKSAIYASTAIRGLLIMFAGWFEFSNFHHSRSTRPWP-------------------------------------------TEDVKAFIQPVHGPLFMYYIVTAA
Croe  VERAYQVKRRRSQLSNMRTAGLVLGLFAIFRVLWD------------------------DRASERSRILRVACAASMFVAASLTASKIVRR----------------------------------------------------IPPALRHSMVALLFAVAGCLL

          <-|                                                                                                                                            |->      M10
PvivA VFSIAYLWAISET-------------------------------------------------------------------------------------------------------------------------------DQTTSYTIWMTNDTIEFFFYLVIL
PfalB ILIDLSCIHYSML------------------------------------------------------------------------------------------------------------------------------LGNKNERALFFMKDASELIICIQLI
Bdiv  IFALSNSWAVSKS------------------------------------------------------------------------------------------------------------------------------GYYSKYLNLWLPSDSIEFYFYIVVL
Tpar  IFALSNSWSIKRT--------------------------------------------------------------------------------------------------------------------------------EHVDYNLWLNSDSVEFYFYLILL
Esti  VFALSNSWSIQTTTRQPAGGGPVTSNLDAASLVSPGVALQTSTLGNEMLTYMRGGRTVRMPENIDLQSPVAFLSHKSNLPQSPYIFYEFCPYEGCGGSTPPSTPASAHGEPPPARRLAAQTGTFAEKTSVSTVNPGSLEIPGQRGASFWLVADSVELFFYLVIL
Crya  ILMLTILWQFTM---------------------------------------------------------------------------------------------------------------------------------VNNSISLWCDEEIIELLL-LATV
Pgig  CLFVLLRSWAYIDVATH-----------------------------------------------------------------------------------------------------------------------------MTVAIPWLYMDSTQLSFFVTLF
Vbra  LISTFQPGWIYAGPIPP-----------------------------------------------------------------------------------------------------------------------------SLCHKSGLFVNTIDLFFWQAIL
Evor  VSSGYTCAVVSARYGLAQN-------------------------------------------------------------------------------------------------------------------------GVEEVAHWVYYESVFYISAMMHLN
Pche  VTMAWVFLFLDENEPTNPHS------------------------------------------------------------------------------------------------------------------------SASANSQLPPDASYHLVTSCLELS
Tpyr  VSILLEIYYMD-------------------------------------------------------------------------------------------------------------------------------------FNDQFVQGRPALLQVLSLPI
Bsto  LLNFHDIDT-----------------------------------------------------------------------------------------------------------------------------------GLIYIKVIQVMYTNIVVSHVSGLP
Pram  FTLAEALLWGVNFEADID---------------------------------------------------------------------------------------------------------------------------TSPRYVNLCLDTVLAMFVVSNGR
Ainv  VLVTPNIFRWTNEQ-------------------------------------------------------------------------------------------------------------------------------QSILYSYISMDVIMPMFLVSSGG
Croe  AAAQAN---------------------------------------------------------------------------------------------------------------------------------------SAQVTLDIVFFTSLCSASGAVHH

     <-|   |->     M11    <-|                                                |->    M12     <-|           HD2      <-||->     CTE2    <-||->                C2
PvivA HHNTGMLFQTCILVDLLFITMSLTFISTSVV----------------------------------KTITTDSTVMLIPWYVTFNLISTYCKESIDRRTFYANESAKKTENRATELLNDMLPKHVLEEFQQDRLKLA---YTHDRLTFLFADICGFTSWANGVDA
PfalB FIKN-MLFKHKFFFFVFFYIFLIYSFSKLFS----------------------------------IHTCQTHICCSIILFISINILYFWYSEYLDRIQFLVKRKRNRMEKISQDFLTKILPRQVLEEYQNDNLQLT---YKHEKIAFLFADIVGFTKWSKTVSP
Bdiv  HHSSGMMFQTCLLVDALFILISLTFISSSVV----------------------------------QTATTSLSLLSIPSYIAFNLISTHCKESIDRRTFYSNEKARMIEARVGQMLNDMLPKSVLEEFKNDKLKMS---YCHEKMSFLFSDIVGFTSWANSVDA
Tpar  HHSTGMLFQNCLLIDSLFLVLSMTFISSSVQ----------------------------------NNENTVTALFTIPICVLFNLLSAHCKESIDRKKYYTNEKAYMIETRINQILSEMLPKSILEEFKLERLKMC---YIHENMSFLFSDIVGFTKWAESVDP
Esti  HHNTGLLFQNCILVDILLLVTCLTFILTAAV----------------------------------STANTIGMVVNFPCYVFFNLVSAYWKEYIDRLTFYVNEHAKTTEGRATQLLNDMLPKQVLEEFQQDRLRLA---YTHDRMTFLFADICGFTSWAKNVDA
Crya  HHNAGLLFRYIILFDVLILFLLATILLTG-------------------------------------ININIEGMTVYVTSLLINIIAAYLRERTGRTIYYSSLIAGNYEKKAHELLITMLPKKVLIDFQEDKLKSA---YIHKNVTFIFSDICGFTQWAMSVEA
Pgig  HHNPSVRYQQVIFFDIALLAILFVSQISHNNKLN---------------------------------YASYVSFIDLPILVGCNLFSTWYREYTQRKTFFIKETMAVNEKRIQQVLLDQLPARLVQDYRNGQLKLA---YVHTETTFLFSDIVGFTGWAKSVDA
Vbra  NFNSQMLFHRLIPFNLAIVVVYSAYALHINLRNLMPLVSEDAPQCACDLGKEVACQCILSKAIACEAVKAIEPILMVVAFMFLIGASVYFKEYFQRVTFVLNEEANEVEQRANLLLQDMLPPQVLAEMKVDSLQLAHG-Y--DQMTFLFADICGFTSYAKTVPP
Evor  VIRASIGAFLCGFLMVLPATALGVFA------------------------------------------DWPAGAIYPVTVYLLQVRIVQSLKRYQLKSLDDLNLVEKEHKECHSLLNSLLPKEVLHSMQSDTLQLA---YTYRDMTLLFADIVGFTKYCSSHSP
Pche  V-WITIINFNAGMLFKHLFFTNILVLTTVMTLWNY--------------------------GDRIDPDVTLRVSFFLLPFGLVNLLMARMMESWERRIYLADIALHRWQKRAQRLLSEFMPPSALEDYLADKHTAS----LFKNMTIMFADICNYTAFATHEPA
Tpyr  VFIYIVFMRMTIFTPKTMILKAILTISAWF------------------------------YSTQKIYETTFQLNYLIVLILILQLLMSATHLKLEMSQFNHSQILEARAKKQNDILMYLLPEHILSKFLNNPNKKIELIDHLDNVTFLFADIAGFTKYSGSVQP
Bsto  IGYILTATVVNFVDWYCVAFSSEHSL-----------------------------------------GRMIEGTFFIVAFFFLNCIASCVREYQARKSYDLNKFAEREIKNTEKLLNQMIPPQVLRNLQNDITTT----DRYTDVTIIYADICGFTSWSSDKKP
Pram  SILYRHAVVFNVLALIMVSLTTLVSLQRYNQDAAK----------------------------FNSKFKTSYPIVLTYFSVVANIIASQSVEFFTRRQIWLKTRTQLETMNADRLLYQMLPAAVVMRLKEGE----MVSDQHQQVGILFSDIKGFTSIASQAAT
Ainv  SLLSKFTSYYNMLLMALSTTIYIYAYQQSLPDYSK------------------------------VEGLKVYPTALTLFVGLFSMMARRDIEFFCRRKYWFQTRAQMETKKADRLLYKMLPQSVVTQLKEGE----LVCDQHHQVGILFSDIKGFTSIAARADT
Croe  LGALFANLVVVSFALGISATNSLDFAGGDS--------------------------------------LTGTAAFFAGIGVVAGTLASATRAYYKRRRFGLEVLTKEETKRAHGILYNMLPAALVNQIKEGKQ---GASDAFRNVTLLFCDIRGFTKMASSVSP
70%                                                                                                                          L  ΨLP  ψψζζΦ                ψTΦLFπDI GFTζΩAζζV
cons                                                                                                                         L   LP                              F D   F  L     P
2o                                                                                                                                                       bbbbbbbbbbbbb hhhhhh
                                                                                                                                                              b1          h1a
```

```
PvivA SEVLTLLQKLFAKFDNDSTKYGLYKLCTIGDAYVAISEP----------------------------------------------------------------------------------------------------------------------------
PfalB KEVLKLLQKLISKIDKDTIKLGLYKLFTIGDAYVATSQP----------------------------------------------------------------------------------------------------------------------------
Bdiv  CDVVALLQKLFARFDRNSTKFGLFKLCSIGDAYVAVSEP----------------------------------------------------------------------------------------------------------------------------
Tpar  SEVIALLQKLFANFDRNTTNFNLYKLCTIGDAYVAISEP----------------------------------------------------------------------------------------------------------------------------
Esti  CEVVTMLQKLFAKFDRDSTKYKLYKLCTIGDAYVAVSEP----------------------------------------------------------------------------------------------------------------------------
Crya  ENVVYMLSTLYAQFDESLSKFGLFKLFTIGDAYVAMSEPEIEINDEIMDRIKNSSVNASANSKSSFQLTNGSGDKKN--------------------------------------------------------------------------------------
Pgig  EKVVDMLQVLYTRFDNDTTKLGIYKVCTIGDAYVAVTEMPVPGE-----------------------------------------------------------------------------------------------------------------------
Vbra  ERVVELLLQLFDRFDDITRETKLYKVCTIGDAYVAVSEP----------------------------------------------------------------------------------------------------------------------------
Evor  NEAVRLVTHLFARFDDCCKLLGAYKVCTIGDAYLAVNEPKTEHDD----------------------------------------------------------------------------------------------------------------------
Pche  ERVVSLLTDLFSRFDHLSDSYGIYKVQS-------------------------------------------------------------------------------------------------------------------------------------
Tpyr  ENVVNMLRILFNEFDQQCQKKNLYKIYTIGDCYVVIGTKNAN-------------------------------------------------------------------------------------------------------------------------
Bsto  IEVVGMLSKLFTNFDHLCVRHNVYKVHTIGDCYVILSFTDS--------------------------------------------------------------------------------------------------------------------------
Pram  AQVVNILASLFCAFDKLTEKHGVFKMQTIGDAYVIVSGLPYVDMSLGPGLVTSSASTADMSNADASMSVASVSAG----------------------------------------------------------------------------------------
Ainv  DKVVHLLDSLFSAFDMLTEKHGVFKMQTIGDAYVIVSGLPYVDMSSKELNNLTEP------------------------------------------------------------------------------------------------------------
Croe  EQVVDVLDKLFCSLDAITDQYEVFKVQTIGDAYVMVAGMPFKDQRVLPDSSAAASEAEALPSATSPKASLSKTTSKLSMLSSGPGGASMGSMVVSSYGQQRFGGSSGSISSSMSSDGDAFVEHFLKQKADAAESNAASTFEHASARTSATNAAQLSTAGSHFL
70%   ζζVVζψLζζLF ζFDζ ζζζ  ψYKψ TIGDAYVΦΦS
cons    VV LLN L               VETIG  YM
2o      hhhhhhhhhhhhhhhhhhhh  bbbbb  bbbbbb
                   h1b            b2     b3

PvivA -------------------------------------------------------------------------------VTEDNQDYDPVDGTERVLEMAYSMIRIIKEIREKLCIPNLNMRIGLHYGSCVGGVIGSGRLRYDLWGIDVLTGNLMESNGVPGK
PfalB -------------------------------------------------------------------------------NSSITDESEALEGILNILKLAKLILHNINTIKIQFNKHDFNMRIGLHYGSCVGGIIGSVRIRYDMWGLDVLIANKIESNGIPGE
Bdiv  -------------------------------------------------------------------------------ATEVPSDQEAMISIESILQMAYSMVRTIQEVRETFSIPGLNMRIGLHYGNSVGGVIGSGRLRYDLWGMDIQTANAMERYGIPGK
Tpar  -------------------------------------------------------------------------------KV-IDNKDMRIQDLENILLMAYSMIHIIDETSKIFSIPDLKMRIGLHYGSCIGGVIGSGRLRYDVWGTDVYTANMIESNGVPGE
Esti  ------------------------------------------------------------------------------VTEDN-DDYDPVEGTSLVLTMAQSMITNILEVRERLNIPSLNMRIGLHYGTCVGGVIGSGRLRYDLWGMDVLTGNMMESNGQPGK
Crya  ---------NDSARNCLYELDDSNSKRTNFSINVFSGNTNSNGSNLRNNFEKCTNTSSSQENSVDFSNTNHRNSELIKKFKICNIQGYSPEEGARRSVAMAHDMLNKISVIRERLNIPDLNMRIGLHYGECIGGIVGSSRIRYEVWGHDIIIGNKMESCGSPGN
Pgig  -------------------------------------------------------------------------------------NFNPYDGANAILELCWHMKKSIRQVAQANKIPNLSMRIGLHFGSCFGGIIGSGRFRYDVWGVDVLTGNLMESNGTPGE
Vbra  ---------------------------------------------------------------------------------VKAQDHDVLGGALKVLRMAHKMIRHIQDVREKLKIPDLNMRIGSHLGYFVGGIIGTKKLRYDIWGLDVLTGNMMESNGEPGK
Evor  ----------------------------------------------------------------------------------------KVLGAVVLIHLAMAMLRIIVTVRNEVQHEGLNMRIGLHHGSFVAGVIGSNQLRFDIWGEDVLIGNQMESNGIPGR
Pche  --------------------------------------------------------------------------------------------ADHMVAFARAMIEALLTVRTEMAAPNLEMRIGLHIGKFVGGVIATTKINYDIFGVDVMIANQVEAAGLPGK
Tpyr  ------------------------------------------------------------------------------------NRLRPHEEARNVVELGFEMIEIIKKVRSIVQFEELEMRIGVHTGSYIGGVIGTDIVRYDIYGPDVMVANKMESNGEQGK
Bsto  ------------------------------------------------------------------------------------EKRNFKEEAVNMVEMALDMVKAIKRVN-KSKGIGLNMRIGMHTGVVVAGITGTNIVRYDIYGPDNDIANKMESKGCPGK
Pram  --------------------------------GLGTTVGRLSSNRVLPLERKSEVVEGPG-GTPRGSGTKLRSHLLRRSTLKHKHQMHLRSHIRDLLAMARDMHKEVRKVQDPNTGERLQMRIGIHIGNIIGGVIGTTTLRYDMWGPDALTANELESNGVPEK
Ainv  --------------------------------------------NSADLVSFQDTALHTIHHKSVASGNHVKPHTES---FRPLESLPPRLHIRNLIRMATDMQREVSKVMDPNSGEPLQMRIGIHIGSIIAGVIGTSTLRYDMWGPDVLTANEMETNGVPGR
Croe  APSTAEPAHWGRTRLHADSKRASKDVGDMDSSHREGNNSSNPVSSVELLRQQSDATMVVSDWSDEDRKVLDEAEAAGIEDPEYRAQHPRRRETAEAMVNVAMDMLQAIKKIKHPGTGEPLVMRIGLHTGDIVGGVIGSKTLRYDIWGTDVLVANKMESEGIPGG
70%                                                                                                  ψψζψA  Mψζ IζζV+   ζ   LζMRIGIH Gζ ΨGGVIGS  ψRYDψWG DVψTAN MESNG PG
cons                                                                                                                           RIGI SG VVAGVVG K  RY LFGDTVNTASRMES G
2o                                                                                           hhhhhhhhhhhhhhhhhhhhhh        bbbbbbbbbbbb         bbb hhhhhhhhhhh     b
                                                                                                          h2                    b4               b5     h3

PvivA INVSETLKNFLLQQFKNRFIFKPHTTVRVIY-KDVKSFIITDKKEIDAMNNSKNLHNTGYLLNRKMRRQNQLDADLLFDGKRPDTFTRSMCNSNSFDNGQPPDRDLYSINVNDGASNS--
PfalB IICSEQFRHFFIQNEPQAKLNFWYYKSISINDQDIKLYVIEDKNYEEDYDPKVIDYTT--LLKLRQKKGLRS------------------------------------------------
Bdiv  ICVSETLKLLLITNFQNRFTFAFNNELRVI-DRCVRSYILTSDVNDNSVF----------------------------------------------------------------------
Tpar  VCVSEKFRDIITRHFPNRFKFEHHKDINII-DKTVKSYIIRNKSKEAMQKSPFSQMNFADAGNV--------------------------------------------------------
Esti  VNVSSVLRDFLLKHFPGKFHFRFNKTVCVINRTVDSYIIHPAGEMSEDGSGVAQPASDVPVLSPPVVLTGAPRREDAVKPEIRGMFSRRGSLISVQRSVARRHSVRNVGEASAPVES---
Crya  ITISGQLYDVLNKNFSQLFKFNYIGSVLIKKQNVDMYRTRMRKNIQLKVNTFSNSKDVSNIIKRNILRYNLRI-----------------------------------------------
Pgig  KTCSEAFKDFLSEHPDQIGRNIADQRFYFKVSLQQPTNQPHAAVNVVTHDVQSFILKDWSFDSMPAEHRALLNQKD--------------------------------------------
Vbra  ICVSHTLKAYLSEH--CKDRFTFKQHKLVTVVEKVQVQSYLLTDSEDTTTHFAPSSDSKTHTKLMSAIDKVRSQHRREAREKAARRRGSQVSTWPRRRGSQASTSMSHLTGIPATRQGSG
Evor  ICCSATFVDVMAQFSKEFSFTEH---KVIDCSGSARQIPSYLLNEPHGIGTGTMDVPKTPKSRRSNRHQSSSRKSHK-------------------------------------------
Pche  IVASSSLRGYLTHHFPGKHRFRF--HKAIEILVPDDSNKSLALSSQQGVVCKQLKLYEISDKKLPSGLFETVM-----------------------------------------------
Tpyr  VMVSKTTMNLLLENYGNRYNFNKGNDVFIKQFHRNIEGYFISQNLN--------------------------------------------------------------------------
Bsto  INVSQVSKAILQEIVPNRFEYQFNTVVTHDPTQRSLDSFFLVPLLEKDIIVDEE------------------------------------------------------------------
Pram  ILVSPVVQEVVKDMIDIRCTFHRKINFTNVPMMDTYLVEFIEPDGTGGKGPASSSGGRRNSGKLPEEIRPAPGYVGSDDGKPSTNMGAEAT-----------------------------
Ainv  ILVSRWVKNVAEDCPDLRFTFHKTIDFTGISKVDTYIAEFVEDKIEKHNLPHHLEPIPADSKTAHAERLSDGSTKLPSKQRRGEANIET-------------------------------
Croe  LVVSHDTRLALIGVENLEFRPHKDVVAKGRGTIKTFQVIKKVKTDGGMIRIGDVELDGDGHGGELDALRQQATEVMPVDAPPSDGLEGPSALPSKHGSPVVKSP----------------
70%   I VS   +  L ζ    +  F
cons  IHVS  T   L   F  R    KG   M  Y L
2o    bbbbbbb
        b6*
```
