# Supplementary material for: Origins and Molecular Features of a Chimeric Type-P4A ATPase—Guanylate Cyclase from Stramenopiles and Alveolates
Source: Int J Mol Sci. 2026 Jul 28;27(15):6734. doi: 10.3390/ijms27156734 (PMC13466520; doi:10.3390/ijms27156734)
Supplement: Supplementary file 1 [file ijms-27-06734-s001.zip › Supplemental Figure S2.pdf]

Supplemental Figure S2. Homology of flippase sequence motifs.

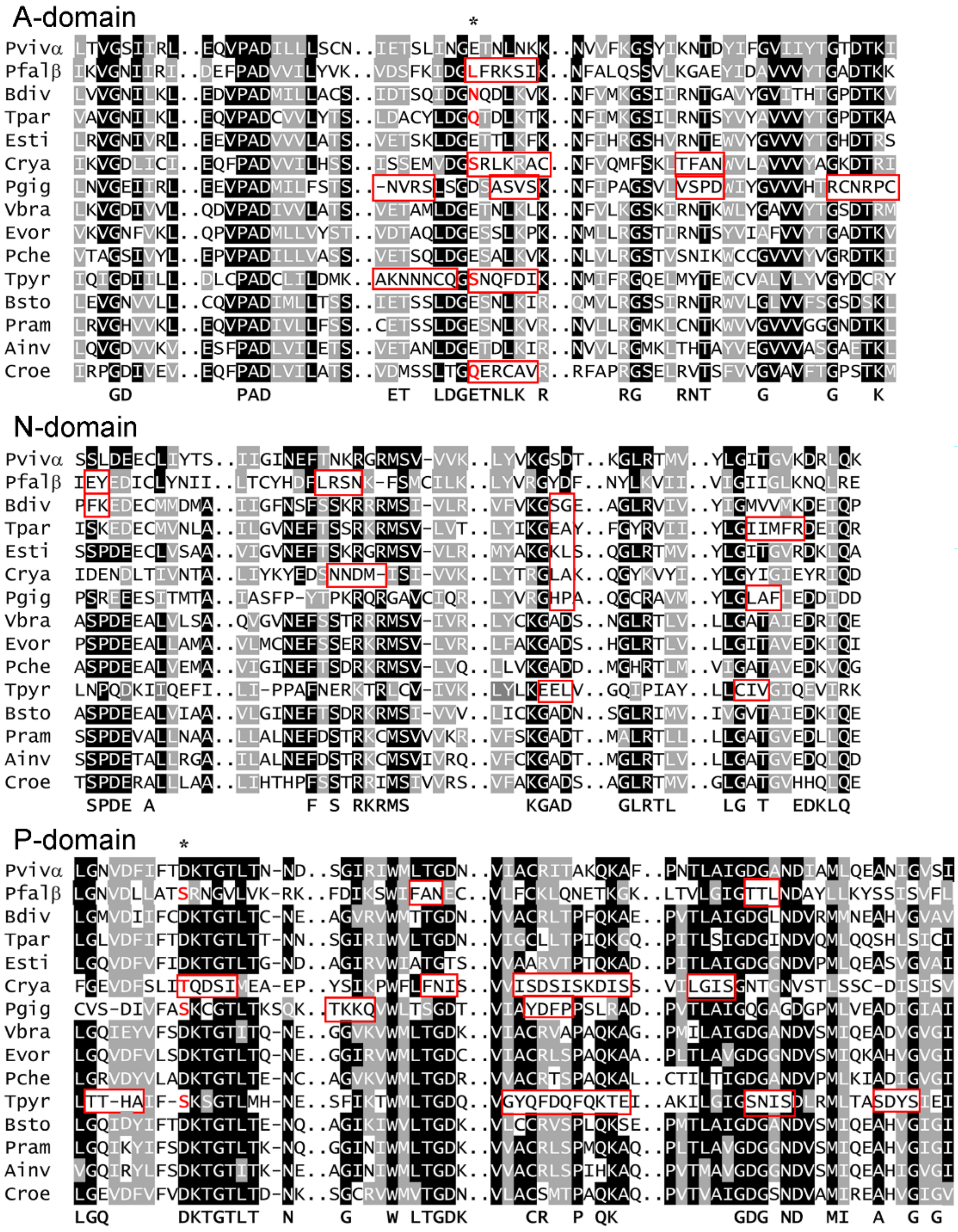

Shown are the major sequence motifs from the A-, N-, and P-domains of P4GC from sequences representing the various phylogenetic groups. Alignments are derived from the annotated alignment (Figure S1) and species are defined in Supplemental Table S1. Asterisks denote the conserved glutamate (E) of the LDGET motif in the A-domain and the phosphorylated aspartate (D) of the DKTGTLT motif in the P-domain. Deviations from the E and D are colored red. The more notable regions of low homology are boxed in red. Below the alignments are the highly conserved residues and motifs.
