# Supplementary material for: Origins and Molecular Features of a Chimeric Type-P4A ATPase—Guanylate Cyclase from Stramenopiles and Alveolates
Source: Int J Mol Sci. 2026 Jul 28;27(15):6734. doi: 10.3390/ijms27156734 (PMC13466520; doi:10.3390/ijms27156734)
Supplement: Supplementary file 1 [file ijms-27-06734-s001.zip › Supplemental Figure S3.pdf]

Supplemental Figure S3. Maximum likelihood tree of P4GC.

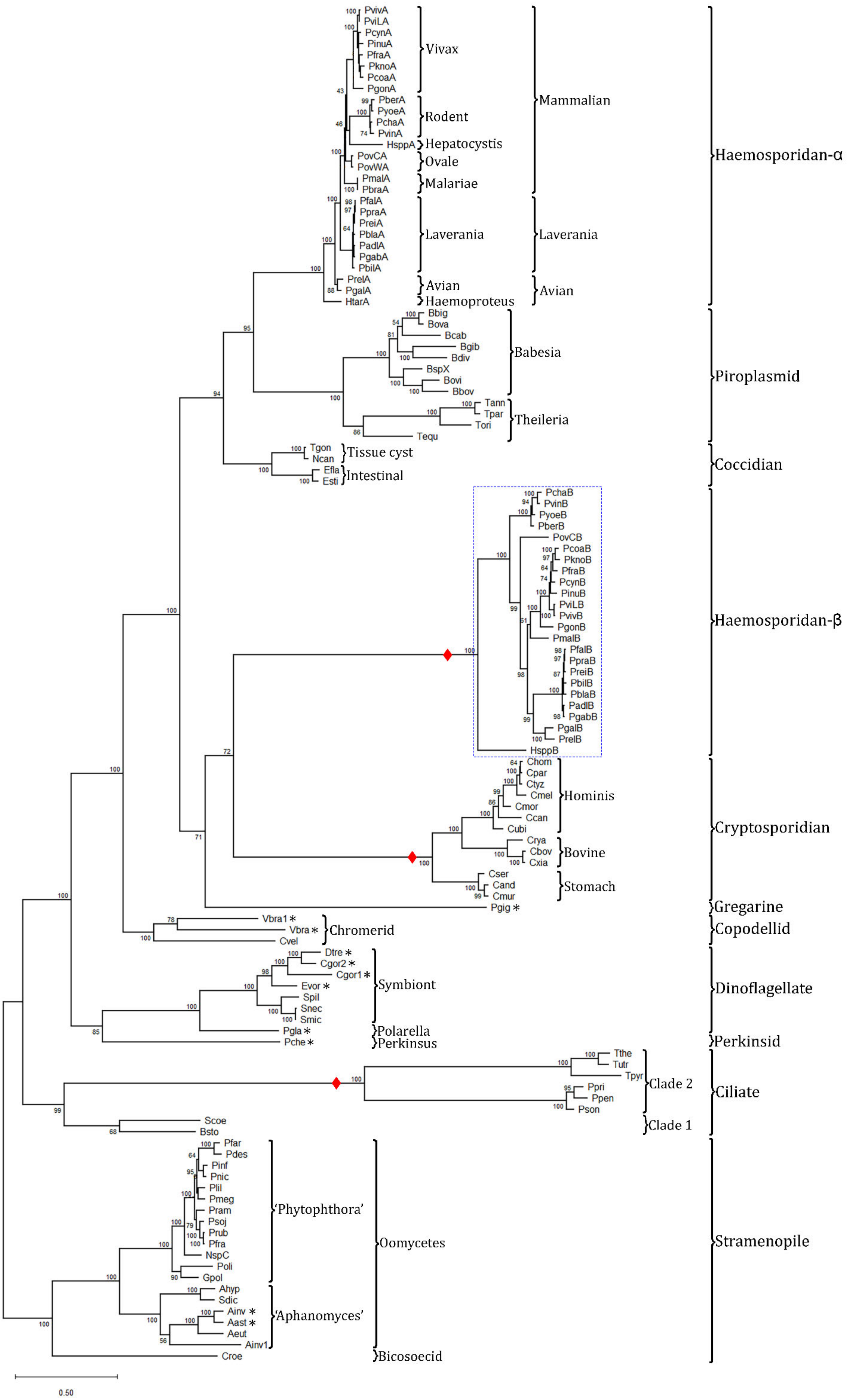

The phylogenetic tree was generated as described in the Materials and Methods. The major phylogenetic groups are shown on the far right, and the various subclades are shown within the tree. Asterisks denote species with possible paralogues. Only the haemosporidan-β (marked with blue dashed rectangle) did not branch at its expected position. This clade also did not exhibit the expected phylogeny of haemosporidans as exhibited by the haemosporidan-α clade. The red diamonds indicate that branches that had a low level of homology in the highly conserved flippase motifs. Bootstrap values are shown at the nodes and were calculated from 500 replicates.
