# Supplementary material for: Origins and Molecular Features of a Chimeric Type-P4A ATPase—Guanylate Cyclase from Stramenopiles and Alveolates
Source: Int J Mol Sci. 2026 Jul 28;27(15):6734. doi: 10.3390/ijms27156734 (PMC13466520; doi:10.3390/ijms27156734)
Supplement: Supplementary file 1 [file ijms-27-06734-s001.zip › Supplemental Figure S4.pdf]

Supplemental Figure S4. Homology models of the flippase module.

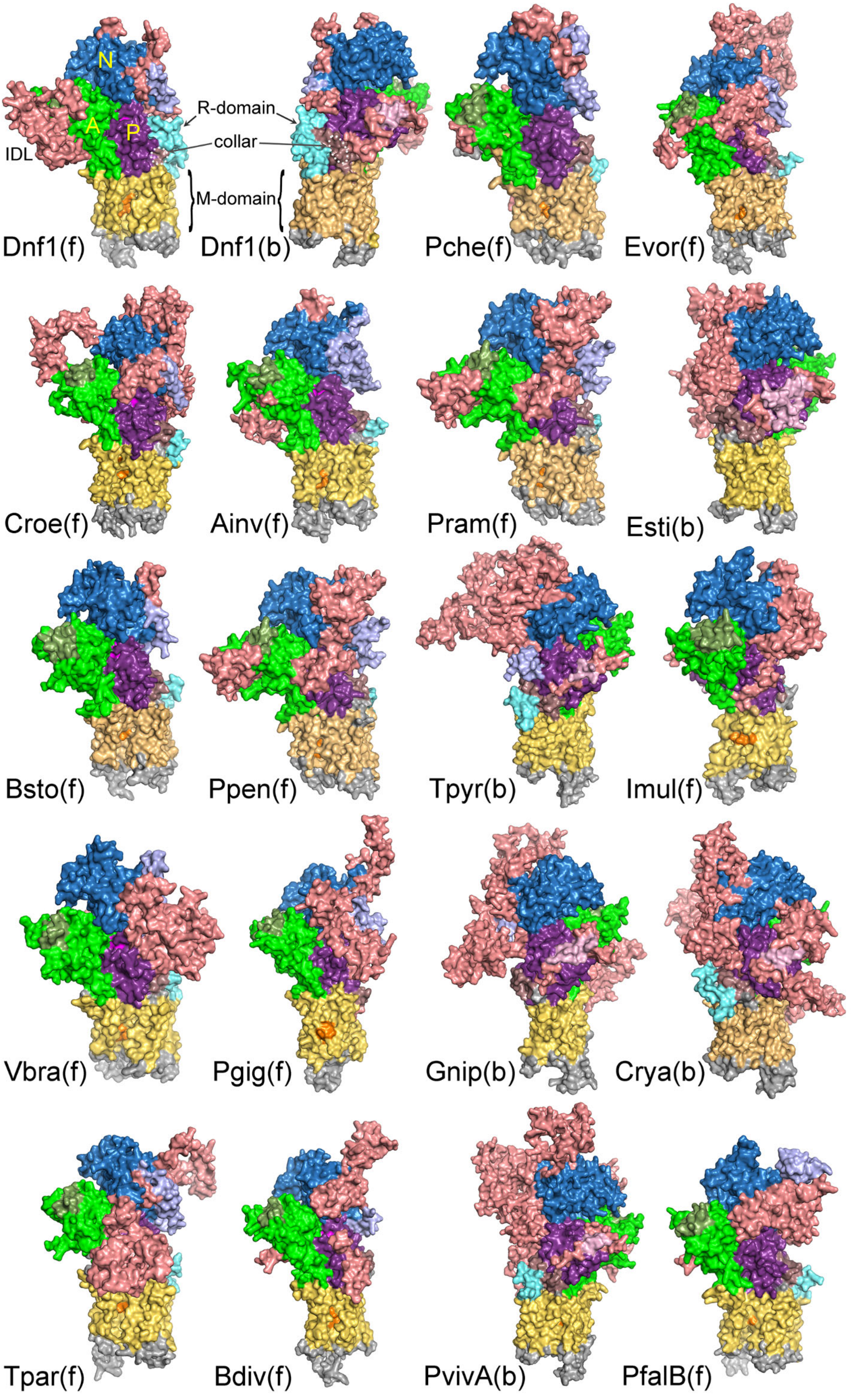

Homology models were produced using Swiss Model with Dnf1 (7drx) as a template and visualized with PyMOL. Representatives from the major phylogenic groups are shown and the abbreviations are defined in Supplemental Table S1. The front (f) view is facing the cargo-binding groove (orange), and the back (b) is rotated approximately 180 degrees. All P4GC models recapitulate the template quite well and the actuator (A)-domain (green), nucleotide binding (N)-domain (blue), phosphorylation (P)-domain (purple), and membrane (M)-domain (yellow) exhibit the expected structures and organization. The regulatory (R)-domain (cyan) is a C-terminal extension that folds back onto the P-domain in the template. The R-domain is truncated or missing in the P4GC models. Those missing the R-domain, are often also missing some of the supporting transmembrane (sTM) helices and thus have a smaller M-domain. A collar-like structure at the base of the P-domain is a brown color and circled with dotted ovals in the template. The salmon color represents intrinsically disordered loops (IDL) and the light-blue color represents helices from variable regions adjacent to the N-domain. Similarly, the light-pink color represents helices from variable regions adjacent to the P-domain and are visible from the back view. The dull green is a helical region between the two β-sheets of the A-domain.
