# Supplementary material for: Origins and Molecular Features of a Chimeric Type-P4A ATPase—Guanylate Cyclase from Stramenopiles and Alveolates
Source: Int J Mol Sci. 2026 Jul 28;27(15):6734. doi: 10.3390/ijms27156734 (PMC13466520; doi:10.3390/ijms27156734)
Supplement: Supplementary file 1 [file ijms-27-06734-s001.zip › Supplemental Figure S5.pdf]

Supplemental Figure S5a. Structure of the cargo-binding groove.

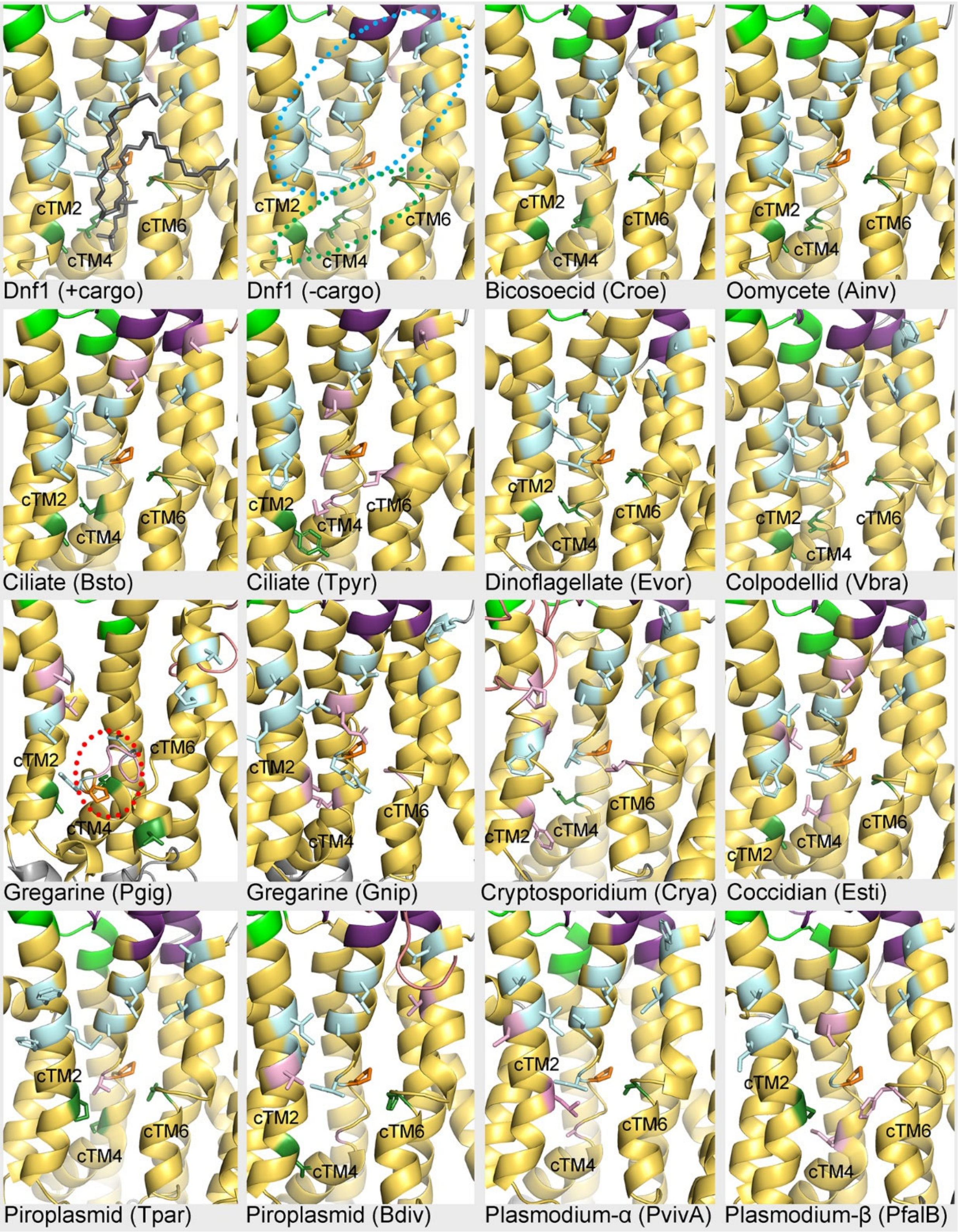

Homology modeling of P4GC from the various phylogenetic groups using Dnf1 as the template was carried out. Shown is the region making up the cargo groove by core transmembrane helices cTM2, cTM4, and cTM6. The invariant proline in the 'kink' of cTM4 and sidechain is shown in orange. Dnf1 is shown with and without its lipid cargo (dark gray sticks). The three polar residues and sidechains that stabilize the polar head group of the lipid cargo are circled with the green-dotted oval and colored green in the Dnf1 without cargo panel. The eight nonpolar residues that make up the hydrophobic gate are colored cyan and circled with the blue-dotted oval in the Dnf1 without cargo panel. The stabilizing and gate residues are colored green and cyan in the P4GC panels if they retain their respective polar or nonpolar properties. Substitutions of polar residues with nonpolar residues or vice versa are colored pink. The red-dotted oval in Pgig panel denotes the disruption of the cargo groove.

Supplemental Figure 5b. Alignment of cargo groove residues.

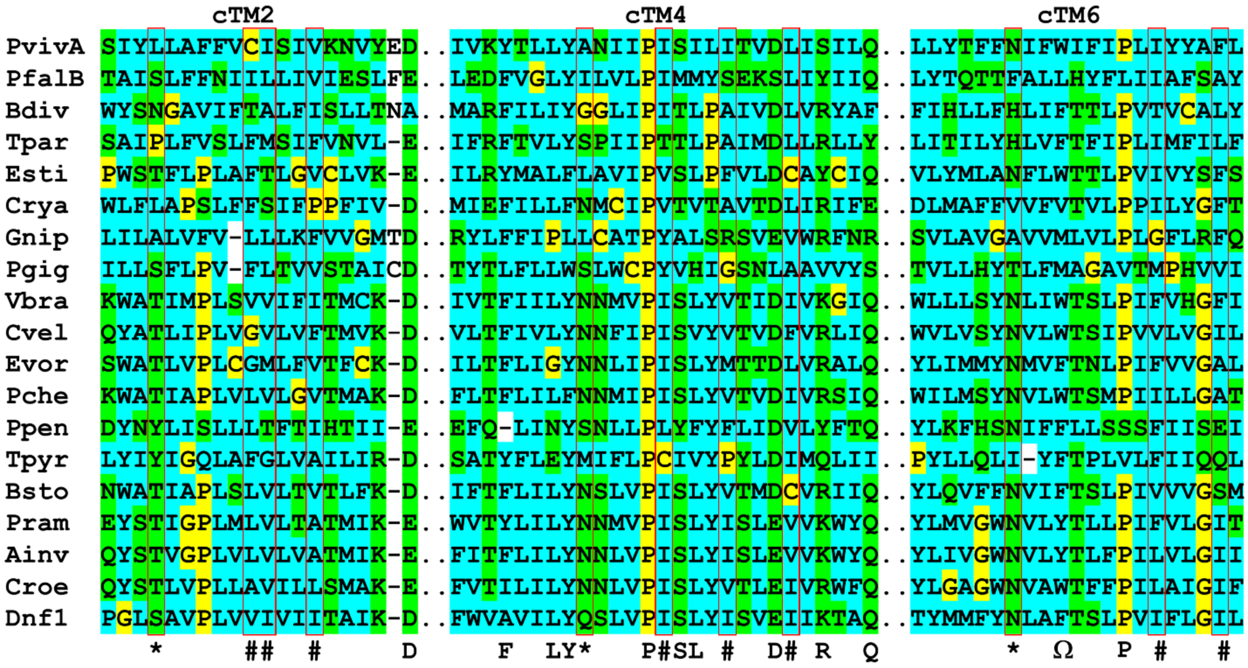

Shown are regions of core transmembrane (cTM) regions making up the cargo groove. Non-polar residues are shaded blue and polar residues are shaded green. G, C, and P are shaded yellow. Note that the polar residues (green) tend to form columns indicating a conservation in the position of polar and nonpolar residues. The residues stabilizing the polar headgroup of the lipid cargo are denoted with * and boxed. Residues forming the hydrophobic gate are denoted with # and boxed. Highly conserved residues are indicated below the alignment. Dnf1 is a flippase from yeast and the other rows are P4GC from various species as defined in Supplemental Table S1.
