# Supplementary material for: Origins and Molecular Features of a Chimeric Type-P4A ATPase—Guanylate Cyclase from Stramenopiles and Alveolates
Source: Int J Mol Sci. 2026 Jul 28;27(15):6734. doi: 10.3390/ijms27156734 (PMC13466520; doi:10.3390/ijms27156734)
Supplement: Supplementary file 1 [file ijms-27-06734-s001.zip › Supplemental Figure S6.pdf]

Supplemental Figure S6. Substrate-binding groove of the cyclase module.

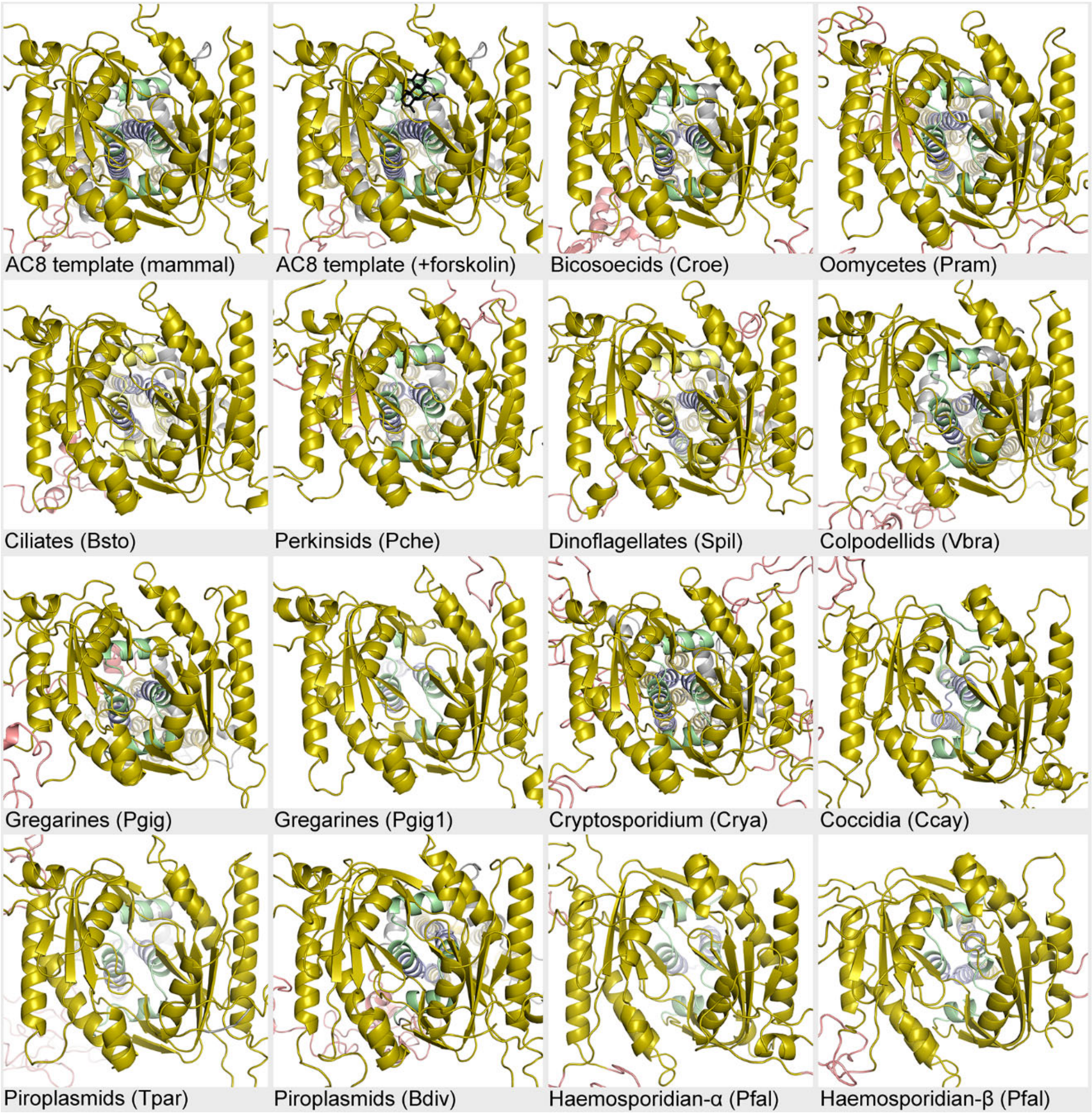

Homology models of P4GC from the various phylogenetic groups were produced with the AC8 template. Shown is the view from the top of the cyclase module with catalytic domain-1 on the left and catalytic domain-2 on the right. All the P4GC proteins are very similar to each other and very similar to the AC8 template. Bound forskolin (black sticks) is shown on one of the templates. Species abbreviations are found in Supplemental Table S1.
