# Supplementary material for: Origins and Molecular Features of a Chimeric Type-P4A ATPase—Guanylate Cyclase from Stramenopiles and Alveolates
Source: Int J Mol Sci. 2026 Jul 28;27(15):6734. doi: 10.3390/ijms27156734 (PMC13466520; doi:10.3390/ijms27156734)
Supplement: Supplementary file 1 [file ijms-27-06734-s001.zip › Supplemental Table S1.pdf]

Supplemental Table S1. Summary of P4GC sequences.

Abbr	Species	Strain	Taxon	Protein ID	Gene ID (or contig)	Flippase	Cyclase 1	Cyclase 2	Comment
	Haemoproteus tartakovskyi	SISKIN1	Haemosporida	n.a.	(Htart_000226000)	n.a.	n.a.	n.a.	Partial sequence corresponding to N-terminus.
HtarA	Haemoproteus tartakovskyi	SISKIN1	Haemosporida	n.a.	(Htart_000410000)	6.60e-89 (cd02073)	1.71e-43 (cd07302)	4.71e-42 (cd07302)	Partial sequence corresponding to C-terminus combined with Htart_000226000 to form the entire sequence.
PgalA	Plasmodium gallinaceum	8A	Haemosporida	CRG96436.1	PGAL8A_00366100	3.18e-95 (cI21460)	5.85e-45 (cd07302)	5.46e-41 (cd07302)	
PrelA	Plasmodium relictum	SGS1-like	Haemosporida	CRH00257.1	PRELSG_0936100	2.25e-94 (cI21460)	2.92e-44 (cd07302)	1.44e-41 (cd07302)	Missing a block of sequence found in Pgal and Htar.
PfalA	Plasmodium falciparum	3D7	Haemosporida	XP_001348065.1	PF3D7_1138400	6.23e-84 (cI21460)	3.17e-33 (cd07302)	4.72e-41 (cd07302)	
PadlA	Plasmodium adleri	G01	Haemosporida	SOV15977.1	PADL01_1136600	2.06e-83 (cI21460)	1.91e-35 (cd07302)	5.72e-41 (cd07302)	
PbilA	Plasmodium billcollinsi	G01	Haemosporida	SPJ11103.1	PBILCG01_1132800	1.97e-83 (cI21460)	4.79e-30 (cd07302)	4.74e-41 (cd07302)	
PblaA	Plasmodium blacklocki	G01	Haemosporida	SOV78234.1	PBLACG01_1136700	3.43e-85 (cI21460)	4.01e-34 (cd07302)	4.77e-41 (cd07302)	
PgabA	Plasmodium gaboni	SY75	Haemosporida	XP_018641298.1	PGSY75_1138400	7.12e-84 (cI21460)	1.05e-35 (cd07302)	4.62e-41 (cd07302)	
PpraA	Plasmodium prafalciparum	G01	Haemosporida	SOS79423.1	PPRFG01_1139100	6.36e-84 (cI21460)	4.97e-34 (cd07302)	4.73e-41 (cd07302)	
PreiA	Plasmodium reichenowi	CDC	Haemosporida	CDO65186.1	PRCDC_1136800	6.33e-85 (cI21460)	8.01e-33 (cd07302)	4.76e-41 (cd07302)	
PgonA	Plasmodium gonderi	ATCC 30045	Haemosporida	XP_028543739.1	PGO_093500	5.17e-90 (cI21460)	6.25e-41 (cd07302)	6.89e-41 (cd07302)	
PviLA	Plasmodium vivax-like	Pvl01	Haemosporida		PVL_090023500	1.05e-94 (cd02073)	1.23e-39 (cd07302)	5.29e-41 (cd07302)	Not in NCBI database.
PvivA	Plasmodium vivix	P01	Haemosporida	VUZ96065.1	PVP01_0939400	8.18e-92 (cI21460)	1.36e-42 (cd07302)	5.33e-41 (cd07302)	
PcoaA	Plasmodium coatneyi	Hackeri	Haemosporida	XP_019914859.1	PCOAH_00027230	1.21e-96 (cI21460)	3.62e-43 (cd07302)	5.18e-41 (cd07302)	

Abbr	Species	Strain	Taxon	Protein ID	Gene ID (or contig)	Flippase	Cyclase 1	Cyclase 2	Comment
PcynA	Plasmodium cynomolgi	M	Haemosporida		PcyM_0943500	1.66e-97 (cd02073)	2.06e-41 (cd07302)	5.31e-41 (cd07302)	Not found in NCBI database and strain B possibly missing some sequence.
PfraA	Plasmodium fragilie	nilgiri	Haemosporida	XP_012333198.1	AK88_00124	8.17e-105 (cl21460)	1.94e-43 (cd07302)	5.16e-41 (cd07302)	
PinuA	Plasmodium inui	San Antonio 1	Haemosporida	XP_008816205.1	C922_02384	4.74e-93 (cl21460)	6.51e-43 (cd07302)	1.55e-40 (cd07302)	
PknoA	Plasmoidum knowesi	H	Haemosporida	XP_002259390.1	PKNH_0936600	8.17e-95 (cl21460)	5.89e-43 (cd07302)	8.58e-41 (cd07302)	
PbraA	Plasmodium brasilianum	Bolivian 1	Haemosporida	XP_067073557.1	MKS88_002983	1.46e-92 (cl21460)	1.02e-39 (cd07302)	1.01e-40 (cd07302)	
PmalA	Plasmodium malariae	UG01	Haemosporida	XP_028861798.1	PmUG01_09047800	1.52e-92 (cl21460)	1.01e-39 (cd07302)	7.57e-41 (cd07302)	Sequence corrected to remove intron and correction submitted to Plasmodb.
PovCA	Plasmodium ovale	curtisi GH01	Haemosporida	SCP04712.1	PocGH01_09044900	6.06e-96 (cl21460)	4.04e-42 (cd07302)	8.27e-42 (cd07302)	Shorter N-terminus than other sequences. Deleted an A upstream of start site and used start codon 147 nucleotides upstream
PovWA	Plasmodium ovale	walliker PowCR01	Haemosporida	SBT77265.1	POWCR01_090040200	4.38e-96 (cl21460)	1.48e-41 (cd07302)	3.50e-41 (cd07302)	Shorter N-terminus than other sequences. Used a start codon 147 nucleotides upstream.
HsppA	Hepatocystis sp.	ex Piliocolobus tephrosceles	Haemosporida	VWU48589.1	HEP_00051800	2.27e-84 (cl21460)	1.04e-39 (cd07302)	1.35e-39 (cd07302)	
PberA	Plasmodium berghei	ANKA	Haemosporida	VUC55644.1	PBANKA_0910300	6.36e-89 (cl21460)	1.48e-46 (cd07302)	9.14e-43 (cd07302)	
PchaA	Plasmodium chabaudi	chabaudi	Haemosporida	VTZ67985.1	PCHAS_0711600	7.01e-90 (cl21460)	2.51e-47 (cd07302)	1.38e-42 (cd07302)	
PvinA	Plasmodium vinckei	vinckei CY	Haemosporida	VEV56560.1	PVVCY_0903710	7.57e-90 (cl21460)	8.43e-48 (cd07302)	9.93e-43 (cd07302)	
PyoeA	Plasmodium yoelii	yoelii 17X	Haemosporida	VTZ78309.1	PY17X_0911700	9.58e-89 (cl21460)	4.21e-47 (cd07302)	1.38e-42 (cd07302)	
	Haemoproteus tartakovskyi	SISKIN1	haemosporidia		Htart_000086000				Partial sequence and not investigated further. Corresponds to P4GC-beta.
PrelB	Plasmodium relictum	SGS1-like	haemosporidia	CRH00754.1	PRELSG_1110100	2.89e-44 (cl21460)	4.14e-41 (cd07302)	5.20e-33 (cl11967)	
PgalB	Plasmodium gallinaceum	8A	haemosporidia	CRG93962.1	PGAL8A_00167000	2.48e-43 (cl21460)	2.37e-40 (cd07302)	1.55e-33 (cd07302)	

Abbr	Species	Strain	Taxon	Protein ID	Gene ID (or contig)	Flippase	Cyclase 1	Cyclase 2	Comment
PfalB	Plasmodium falciparum	3D7	haemosporidia	CAD52725.2	PF3D7_1360500	2.42e-48 (cl21460)	1.01e-38 (cd07301)	9.88e-34 (cd07302)	
PadlB	Plasmodium adleri	G01	haemosporidia	SOV18425.1	PADL01_1359500	1.37e-47 (cl21460)	1.55e-39 (cl11967)	9.48e-34 (cd07302)	
PbilB	Plasmodium billcollinsi	G01	haemosporidia	CAC9699208.1	PBILCG01_1359600	7.07e-48 (cl21460)	2.84e-39 (cl11967)	9.66e-34 (cd07302)	
PblaB	Plasmodium blacklocki	G01	haemosporidia	SOV81526.1	PBLACG01_1357900	1.83e-45 (cl21460)	2.93e-39 (cl11967)	9.68e-34 (cd07302)	
PgabB	Plasmodium gaboni	SY75	haemosporidia	XP_018640373.1	PGSY75_1360500	2.28e-47 (cl21460)	1.41e-39 (cl11967)	5.60e-34 (cd07302)	
PpraB	Plasmodium praefalciparum	G01	haemosporidia	SOS80708.1	PPRFG01_1362300	1.56e-48 (cl21460)	3.51e-39 (cl11967)	9.56e-34 (cd07302)	
PreiB	Plasmodium reichenowi	CDC	haemosporidia	CDO66386.1	PRCDC_1359400	2.74e-49 (cl21460)	3.84e-39 (cl11967)	9.10e-33 (cd07302)	
PgonB	Plasmodium gonderi	ATCC 30045	haemosporidia	XP_028544240.1	PGO_111000	8.69e-42 (cl21460)	7.94e-38 (cl11967)	4.45e-32 (cd07302)	
PviLB	Plasmodium vivax-like	Pvl01	haemosporidia		PVL_110013400	4.39e-50 (tigr01652)	2.74e-36 (cd07302)	7.45e-34 (cd07302)	Not in NCBI database.
PvivB	Plasmodium vivax	P01	haemosporidia	VUZ96857.1	PVP01_1111400	6.33e-48 (cl21460)	1.46e-36 (cd07302)	7.39e-34 (cd07302)	
PfraB	Plasmodium fragile	nilgiri	haemosporidia	XP_012336134.1	AK88_03088	6.72e-46 (cl21460)	5.15e-38 (cd07302)	1.67e-33 (cd07302)	Extra intron in beginning and wrong start corrected and submitted to Plasmodb.
PcoaB	Plasmodium coatneyi	Hackeri	haemosporidia	XP_019915795.1	PCOAH_00032880	9.41e-42 (cl21460)	2.82e-38 (cl11967)	7.04e-34 (cd07302)	Corrected an intron.
PcynB	Plasmodium cynomolgi	M	haemosporidia		PcyM_1111100	8.36e-44 (tigr01652)	2.62e-35 (cd07302)	4.80e-33 (cd07302)	Not in NCBI database.
PinuB	Plasmodium inui	San Antonio 1	haemosporidia		C922_01093	7.04e-43 (tigr01652)	6.29e-39 (cd07302)	6.00e-34 (cd07302)	Extra intron removed and corrected sequence submitted to Plasmodb.
PknoB	Plasmodium knowlesi	H	haemosporidia	SBO24782.1	PKNH_1110800	7.02e-46 (cl21460)	3.09e-38 (cd07302)	1.02e-33 (cd07302)	
PmalB	Plasmodium malariae	UG01	haemosporidia	SCO93004.1	PmUG01_11025500	5.74e-48 (cl21460)	5.58e-34 (cd07302)	1.23e-33 (cd07302)	

Abbr	Species	Strain	Taxon	Protein ID	Gene ID (or contig)	Flippase	Cyclase 1	Cyclase 2	Comment
PovCB	Plasmodium ovale	curtisi GH01	haemosporidia	SCP04899.1	PocGH01_11020000	5.60e-40 (cI21460)	1.41e-38 (cI11967)	3.79e-31 (cd07302)	Extra intron is 2 A's in a poly-A tract.
	Plasmodium brasilianum	Bolivian 1	haemosporidia	XP_067072402.1	MKS88_003590	2.15e-34 (tigr01652)	missing	missing	Missing cyclase domains.
HsppB	Hepatocystis sp.	Piliocolobus tephrosceles	haemosporidia	VVF14437.1	HEP_00088700	6.05e-49 (cI21460)	5.25e-35 (cI11967)	1.38e-31 (cI11967)	Extra intron is 14 A's in poly-A tract. Phyre search suggests structure is different than others. A lot of sequence variation.
PberB	Plasmodium bergei	ANKA	haemosporidia	VUC56788.1	PBANKA_1136700	2.61e-49 (cI21460)	5.47e-40 (cI11967)	6.95e-32 (cd07302)	
PyoeB	Plasmodium yoelii	yoelii 17X	haemosporidia	VTZ79654.1	PY17X_1138200	4.18e-50 (cI21460)	8.40e-40 (cI11967)	8.49e-32 (cI11967)	
PvinB	Plasmodium vinckei	petteri CR2020	haemosporidia	CAD2108931.1	PVPCR_1103700	1.12e-52 (cI21460)	1.83e-39 (cI11967)	6.51e-32 (cd07302)	
PchaB	Plasmodium chabaudi	chabaudi	haemosporidia	VTZ69357.1	PCHAS_1136200	4.27e-54 (cI21460)	2.16e-39 (cI11967)	2.05e-31 (cd07302)	
Bovi	Babesia ovis		piroplasmids	GFE52627.1	BaOVIS_000310	9.92e-132 (cI21460)	1.64e-43 (cI11967)	8.14e-42 (cd07302)	
Bbov	Babesia bovis	T2Bo	piroplasmids	XP_001608739.1	BBOV_I000770	1.15e-122 (cI21460)	2.49e-40 (cI11967)	1.35e-41 (cd07302)	
Bdiv	Babesia divergens	1802A	piroplasmids	KAK1938693.1	X943_003464	1.60e-117 (cI21460)	6.51e-46 (cI11967)	3.04e-37 (cd07302)	
Bbig	Babesia bigemina		piroplasmids	XP_012768395.1	BBBOND_0301140	3.79e-126 (cI21460)	2.85e-44 (cI11967)	1.41e-37 (cI11967)	
Bgib	Babesia gibsoni		piroplasmids	KAK1444216.1	BgAZ_101220	3.31e-121 (cI21460)	4.52e-46 (cI11967)	4.66e-38 (cd07302)	
BspX	Babesia sp.	Xinjiang	piroplasmids	XP_028872523.1	BXIN_0928	4.99e-130 (cI21460)	1.95e-44 (cI11967)	3.75e-40 (cd07302)	
Bcab	Babesia caballi	USDA-D6B2	piroplasmids	XP_067714000.1	BcabD6B2_13640	1.35e-127 (cd02073)	2.47e-45 (pfam00211)	4.24e-38 (pfam00211)	Intron at C-terminus may be in error (= conserved region), and was corrected.
Bova	Babesia ovata	Miyake	piroplasmids	XP_028868938.1	BOVATA_041880	2.27e-129 (cd02073)	3.17e-44 (cd07302)	3.12e-32 (pfam00211)	Looks like missing a membrane domain. Corrected erroneous intron.
Bovi	Babesia ovis	Selcuk	piroplasmids	GFE52627.1		9.92e-132 (cd02073)	1.64e-43 (pfam00211)	8.14e-42 (cd07302)	

Abbr	Species	Strain	Taxon	Protein ID	Gene ID (or contig)	Flippase	Cyclase 1	Cyclase 2	Comment
Tequ	Theileria equi	WA	piroplasmids	XP_004833019.1	BEWA_036030	1.40e-144 (cI21460)	9.67e-47 (cI11967)	6.41e-40 (cd07302)	
Tpar	Theileria parva	Muguga	piroplasmids	XP_061161450.1	TpMuguga_02g00848	4.91e-106 (cI21460)	8.02e-40 (cI11967)	3.61e-42 (cd07302)	
Tann	Theileria annulata	Ankara C9	piroplasmids	XP_951863.1	TA15475	1.08e-109 (cl21460)	1.69e-40 (pfam00211)	7.90e-42 (cd07302)	Updated gene ID 3861617
Tori	Theileria orientalis	Shintoku	piroplasmids	XP_009690953.1	TOT_020001098	3.08e-112 (tigr01652)	1.58e-43 (pfam00211)	6.05e-41 (cd07302)	
	Cytauxzoon felis	winnie	piroplasmids	n.a.	(JAIQCW010000339.1, JAIQCW010000004.1, JAIQCW010000012.1)	1.76e-90 (cd02073)	1.50e-16 (smart00044)	6.57e-41 (cd07302)	Assembled from three contigs. Missing some conserved sequence in cyclase-1.
	Cardiosporidium cionae		nephromycids	KAF8821741.1		3.78e-96 (cd02073)	2.32e-15 (cd07302)	n.a.	Missing cyclase-2 module
Tgon	Toxoplasma gondii	ME49	coccidia	XP_018638137.1	TGME49_254370	1.56e-100 (cI21460)	1.83e-39 (cd07302)	6.69e-39 (cI11967)	
Ncan	Neospora canium	Liverpool	coccidia	XP_003880431.1	NCLIV_008660	2.46e-100 (cI21460)	1.21e-40 (cd07302)	6.75e-39 (cI11967)	
Bbes	Besnoitia besnoiti	Bb-Ger1	coccidia	XP_029216654.1	BESB_012570	1.98e-100 (cI21460)	3.48e-39 (cd07302)	3.70e-40 (cI11967)	Missing some of P-domain and has an insert between b10 and b11 of A-domain.
	Sarcocystis calchasi		coccidia	CAL7857989.1		5.56e-98 (cI21460)	1.73e-39 (cd07302)	1.91e-40 (smart00044)	Missing small part of N-terminus and low homology in N-domain and cyclase module.
	Eimeria praecox		coccidia	CDI75902.1	EPH_0025580	2.19e-16 (cd02703)	4.49e-34 (pfam00211)	6.54e-31 (smart00044)	Missing some of N-terminus and may have error in C-terminus.
	Eimeria acervulina		coccidia	XP_013248102.1	EAH_00028760	4.92e-17 (cd02073)	1.46e-19 (pfam00211)	1.40e-35 (pfam00211)	Low homology in P-domain and cyclase module.
Enec	Eimeria necatrix		coccidia	XP_013436202.1	ENH_00041780"	2.63e-17 (cd02073)	1.82e-34 (pfam00211)	5.56e-35 (smart00044)	Low homology in P-domain
Eten	Eimeria tenella		coccidia	XP_013235760.1	ETH_00028160	3.31e-16 (cd02073)	1.15e-41 (cd07302)	4.75e-38 (smart00044)	Low homology in P-domain. Corresponds to previously identified EtATPase-GC-alpha.[1]
Eten1	Eimeria tenella		coccidia	XP_013229212.1	ETH_00026685	8.29e-106 (cd02073)	1.85e-43 (cd07302)	5.68e-38 (smart00044)	Missing some of N-terminus. Corresponds to previously identified EtATPase-GC-beta.[1]
	Eimeria magna	A19	coccidia	KAL8441686.1	Emag_006978	2.65e-94 (TIGR01652)	1.28e-41 (pfam00211)	4.60e-19 (pfam00211)	Insert in cTM2 and missing cTM5 stalk. Low homology in cyclase module

Abbr	Species	Strain	Taxon	Protein ID	Gene ID (or contig)	Flippase	Cyclase 1	Cyclase 2	Comment
	Eimeria media	PL19_A22	coccidia	KAL8451743.1	Emed_001905	3.65e-103 (TIGR01652)	1.97e-41 (pfam00211)	7.05e-38 (smart00044)	Missing N-terminus.
Esti	Eimeria stiedai	Esti_A26	coccidia	KAL8271382.1	Esti_004716	8.37e-112 (cd02073)	8.56e-44 (cd07302)	2.48e-38 (smart00044)	
Efla	Eimeria flavescens	Efla_A23	coccidia	KAL8440448.1	Efla_005590	2.68e-119 (TIGR01652)	3.84e-39 (cd07302)	9.13e-39 (smart00044)	
	Cyclospora cayetanensis	NF1_C8	coccidia	XP_026190015.1	LOC34622156	2.79e-102 (cd02073)	1.72e-36 (pfam00211)	5.88e-27 (07302)	Missing N-terminus including A-domain. Low homology in cyclase-2 module.
Csui	Cystoisospora suis	Wein 1	coccidia	XP_067924274.1	CSUI_003554	5.64e-103 (cd02073)	1.21e-42 (cd07302	4.53e-39 (cd07302	Missing some of flippases module
Crya	Cryptosporidium ryanae	45019	cryptosporidia	KAH8741966.1	FG386_002341	4.17e-25 (cI21460)	4.96e-34 (cd07302)	9.96e-26 (cd07302)	
Cxia	Cryptosporidium xiaoi	52996	cryptosporidia	KAK6589758.1		1.65e-31 (cI21460)	2.07e-35 (cd07302)	5.18e-28 (cd07302)	
Cmur	Cryptosporidium muris	RN66	cryptosporidia	XP_002139554.1	CMU_042790	1.66e-28 (cI21460)	1.46e-30 (cd07302)	8.21e-36 (cd07302)	
Cand	Cryptosporidium andersoni		cryptosporidia	XP_067069740.1	cand_038500	1.70e-28 (cI21460)	1.34e-30 (cd07302)	1.31e-35 (cd07302)	
Cser	Cryptosporidium serpentis	8845_47	cryptosporidia	KAL7066685.1	ACR3K2_28390	2.04e-35 (TIGR01652)	2.49e-30 (cd07302)	9.71e-37 (cd07302)	
Ccan	Cryptosporidium canis		cryptosporidia	KAJ1609099.1		1.16e-28 (cI21460)	9.69e-31 (cI11967)	1.69e-28 (cd07302)	
Cubi	Cryptosporidium ubiquitum	39726	cryptosporidia	XP_028872886.1	cubi_00923	1.95e-25 (cI21460)	2.35e-31 (cI11967)	2.85e-21 (cd07302)	
Cmor	Cryptosporidium mortiferum	chipmunk genotype I	cryptosporidia	XP_067202702.1	ELE39_000944	7.11e-34 (cI21460)	3.40e-32 (cd07302)	5.82e-28 (cd07302)	
Cbov	Cryptosporidium bovis	45015	cryptosporidia	KAH7649283.1	FG379_001640	4.25e-33 (cd02073)	9.68e-36 (cd07302)	5.70e-27 (cd07302)	
Cmel	Cryptosporidium meleagridis		cryptosporidia	KAK9173417.1		4.07e-27 (cI21460)	1.95e-31 (cd07302)	7.22e-30 (cd07302)	
Ctyz	Cryptosporidium tyzzeri		cryptosporidia	TRY52288.1		2.67e-34 (cI21460)	1.75e-32 (cd07302)	7.16e-32 (cd07302)	

Abbr	Species	Strain	Taxon	Protein ID	Gene ID (or contig)	Flippase	Cyclase 1	Cyclase 2	Comment
	Cryptosporidium sp.	43IA8	cryptosporidia	WKS76853.1		8.39e-31 (cI21460)	7.95e-31 (cd07302)	3.01e-29 (cd07302)	Collected from calf and nearly identical to C parvum
Cpar	Cryptosporidium parvum		cryptosporidia	QOY42460.1	CPATCC_0032070	8.39e-31 (cI21460)	7.95e-31 (cd07302)	3.01e-29 (cd07302)	
Chom	Cryptosporidium hominus		cryptosporidia	PPS92874.1	GY17_00002634	4.91e-31 (cI21460)	3.30e-31 (cd07302)	3.00e-29 (cd07302)	
Gnip1	Gregarina niphandrodes		gregarines	XP_011133204.1	GNI_166520	2.87e-14 (cI21460)	3.32e-42 (pfam00211)	8.31e-34 (cd07302)	Missing some of flippases module and low homology in cyclase module
Gnip	Gregarina niphandrodes		gregarines	XP_011130546.1	GNI_078020	0.00e+00 (cd02073)	1.73e-22 (cd07302)	1.48e-36 (cd07302)	Has substantially longer N-terminus than XP_011133204.1. Low homology in cyclase-1 module.
Pgig	Porospora cf. gigantea	A	gregarines	KAH0481417.1	KVP18_000282	6.31e-43 (cI21460)	6.31e-43 (cI11967)	4.09e-36 (cI11967)	Added 41 aa to N-terminus due to alternate ATG start.
Pgig1	Porospora cf. gigantea	B	gregarines	XP_068374523.1	KVP17_000348	8.89e-8 (TIGR01652)	1.12e-37 (cd07302)	5.33e-36 (cd07302)	Missing kink and motif. Low homology in cyclase-2 module
Vbra	Vitrella brassicaformis	CCMP3155	colpodellids	CEL99327.1	Vbra_20607	0e+00 (cI21460)	2.10e-47 (cI11967)	7.32e-40 (cI11967)	
Vbra1	Vitrella brassicaformis	CCMP3156	colpodellids	CEM36626.1	Vbra_19183	0.00e+00 (cd02073)	1.67e-45 (cd07302)	6.14e-30 (cd07302)	Large insert in cyclase-2 module.
Cvel	Chromera velia	CCMP2878	colpodellids	CEM38262.1	Cvel_5641	1.16e-166 (cd02073)	1.19e-27 (pfam00211)	5.83e-17 (cd07302)	
Pmar	Perkinsus marinus	ATCC 50983	perkinsids	XP_002775192	Pmar_PMAR024448	0e+00 (cI21460)	9.47e-47 (cI11967)	2.27e-36 (cI11967)	Missing M12, HD2 and part of CTE2 in cyclase-2 module.
	Perkinsus chesapeaki	ATCC PRA-425	perkinsids	KAF4666545.1	FOL47_004044	0e+00 (cI21460)	2.94e-45 (pfam00211)	1.09e-29 (cI11967)	Has several regions of inhomology.
Pche1	Perkinsus chesapeaki	ATCC PRA-425	perkinsids	KAF4666911.1		0.00e+00 (cd02073)	5.69e-38 (cd07302)	4.54e-31 (cd07302)	Has longer N-terminus than KAF4666545.1 and other dinoflagellates and missing cTM1. Low homology in cyclase module
Dtre	Durusdinium trenchii		dinoflagellates	CAK9114172.1	CCMP2556_LOCUS52806	0e+00 (cI21460)	2.94e-42 (cd07302)	9.28e-41 (smart00044)	Located on contig CAXAMN010027961.1
	Durusdinium trenchii		dinoflagellates	CAK9114170.1	CCMP2556_LOCUS52806	0.00e+00 (cI21460)	2.99e-42 (cd07302)	8.99e-40 (cd07302)	Has longer N-terminus than CAK9114172.1 and same contig CAXAMN010027961.1; likely a splice variant.
Evor	Effrenium voratum		dinoflagellates	CAJ1373389.1		0.00e+00 (cI21460)	5.39e-46 (cd07302)	6.27e-44 (smart00044)	

Abbr	Species	Strain	Taxon	Protein ID	Gene ID (or contig)	Flippase	Cyclase 1	Cyclase 2	Comment
	Effrenium voratum		dinoflagellates	CAJ1365404.1	EVOC421_LOCUS29553	0.00e+00 (cd02073)	4.32e-46 (cd07302)	missing	Nearly identical to CAJ1373389.1 in overlapping area and missing cyclase-2 module.
Cgor	Cladocopium goreaui		dinoflagellates	CAL1137706.1	C1SCF055_LOCUS11873	0e+00 (cI21460)	6.88e-42 (cd07302)	4.57e-40 (cI11967)	Missing sequence in A-domain
Cgor1	Cladocopium goreaui		dinoflagellates	CAL1170370.1	C1SCF055_LOCUS41673	0.00e+00 (cd02073)	5.44e-43 (cd07302)	3.62e-38 (smart00044)	
Smic	Symbiodinium microadriaticum		dinoflagellates	OLP78940.1	AK812_SmicGene40830	0e+00 (cI21460)	3.83e-41 (cd07302)	3.83e-41 (smart00044)	
Spil	Symbiodinium pilosum		dinoflagellates	CAE7720361.1	SPIL2461_LOCUS20518	0e+00 (cI21460)	1.18e-42 (cd07302)	2.69e-44 (smart00044)	
	Symbiodinium sp.	KB8	dinoflagellates	CAE7948067.1	SMICCKB8_LOCUS40667	0e+00 (cI21460)	5.15e-40 (cd07302)	4.03e-42 (smart00044)	Has ubiquitin and SOD domains at C-terminus
Sspp	Symbiodinium sp.	CCMP2592	dinoflagellates	CAE7681267.1	STRI2592_LOCUS34051	0.00e+00 (cd02073)	1.64e-42 (cd07302)	1.72e-40 (smart00044)	Missing some of N-terminus
Snec	Symbiodinium necroappetens		dinoflagellates	CAE7915210.1	SNEC2469_LOCUS31341	0.00e+00 (cd02073)	2.41e-41 (cd07302)	4.15e-42 (smart00044)	
Snat	Symbiodinium natans		dinoflagellates	CAE7256225.1	SNAT2548_LOCUS13126	0.00e+00 (cd02073)	4.23e-43 (cd07302)	3.24e-43 (smart00044)	Missing N-terminus including some of A-domain
Pgla	Polarella glacialis		dinoflagellates	CAE8635430.1	PGLA1383_LOCUS51031	0e+00 (cI21460)	1.92e-47 (cd07302)	4.34e-39 (cI11967)	CAE8722507.1 is identical
Pgla1	Polarella glacialis		dinoflagellates	CAE8621686.1	PGLA1383_LOCUS39204	2.51e-129 (TIGR01652)	1.53e-48 (cd07302)	1.34e-41 (pfam00211)	Missing N-terminus
	Polarella glacialis		dinoflagellates	CAE8593318.1	PGLA1383_LOCUS11915	0.00e+00 (cd02073)	1.75e-36 (cd07302)	missing	Missing cyclase-2 module
Pcor	Prorocentrum cordatum	s5338	dinoflagellates	CAK0875724.1	PCOR1329_LOCUS60310	0.00e+00 (cd02073)	3.17e-49 (cd07302)	8.95e-39 (pfam00211)	Has extended N- and C-termini as compared to other dinoflagellates. Small deletion near KGAD of N-domain. Insert in cyclase-2.
Ptet	Paramecium tetraurelia		ciliates	XP_001450116.1	GSPATT00017090001	7.19e-21 (cI21460)	6.08e-42 (cd07302)	7.55e-47 (smart00044)	At least 11 paralogous alleles.
Poct	Paramecium octaurelia		ciliates	CAD8157712.1	POCTA_138.1.T0340110	9.96e-20 (cI21460)	5.81e-43 (cd07302)	6.69e-48 (smart00044)	At least 13 paralogous alleles, Deletion in sTM8.
Ppri	Paramecium primaurelia		ciliates	CAD8110446.1	PPRIM_AZ9-3.1.T1440053	1.74e-22 (cI21460)	1.18e-42 (cd07302)	9.34e-48 (smart00044)	At least 10 paralogous alleles.

Abbr	Species	Strain	Taxon	Protein ID	Gene ID (or contig)	Flippase	Cyclase 1	Cyclase 2	Comment
Ppen	Paramecium pentaurelia		ciliates	CAD8173491.1	PPENT_87.1.T0590152	1.35e-22 (cI21460)	5.78e-43 (cd07302)	1.46e-48 (smart00044)	At least 10 paralogous alleles.
Pson	Paramecium sonneborni		ciliates	CAD8121419.1	PSON_ATCC_30995.1.T1320070	9.26e-25 (cI21460)	1.76e-41 (cd07302)	1.51e-46 (smart00044)	At least 2 paralogous alleles.
Pbur	Paramecium bursaria	186b	ciliates	KAM3142064.1	pb186bvf_005937	3.06e-21 (cd02073)	3.06e-42 (cd07302)	1.86e-48 (smart00044)	Modeling ends after cTM5.
Imul	Ichthyophthirius multifiliis	G5	ciliates	XP_004032157.1	IMG5_129030	5.81e-40 (TIGR01652)	1.28e-38 (cd07302)	1.41e-49 (pfam00211)	At least 2 paralogous alleles. Long C-terminus. Start modeling in A-domain
Tmal	Tetrahymena malaccensis	23b	ciliates	KAL4438452.1	ABPG74_009491	1.87e-11 (TIGR01652)	6.07e-48 (cd07302)	4.51e-46 (smart00044)	17 amino-acid insert in cTM4
Tthe	Tetrahymena thermophila	SB210	ciliates	XP_012650719.1	TTHERM_000549649	3.46e-13 (cI21460)	4.94e-48 (cd07302)	9.36e-46 (smart00044)	
Tutr	Tetrahymena utriculariae		ciliates	KAL4486421.1		1.89e-13 (cI21460)	1.47e-47 (cd07302)	6.78e-45 (smart00044)	Modeling ends after cTM7.
Tpyr	Tetrahymena pyriformis		ciliates	CAB52247.1		2.41e-16 (cI21460)	9.19e-46 (cd07302)	5.59e-48 (smart00044)	Added 8 residues to N-terminus from CAB52252.1. Modeling ends after sTM8.
Bsto	Blepharisma stoltei		ciliates	CAG9334296.1	BSTOLATCC_MIC60915	0.00e+00 (cd02073)	5.05e-48 (cd07302)	2.53e-50 (smart00044)	
Scoe	Stentor coeruleus		ciliates	OMJ74766.1	SteCoe_26245	0.00e+00 (cI21460)	6.96e-49 (cd07302)	1.20e-51 (smart00044)	At least 8 paralogous alleles.
Ainv2	Aphanomyces invadans	NMJ9701	oomycetes	ETW10348.1	H310_00671	0e+00 (cd02073)	1.88e-48 (pfam00211)	1.28e-46 (smart00044)	Partial TxxLDGETxxK motif and insert in highly conserved region of A-domain
Ainv1	Aphanomyces invadans	NMJ9701	oomycetes	ETW08630.1	H310_01166	0.00e+00 (cd02073)	8.12e-47 (cd07302)	4.82e-51 (smart00044)	Low homology around M7-M8 or cyclase-2 module
Ainv	Aphanomyces invadans	NMJ9701	oomycetes	ETW08645.1	H310_01180	0e+00 (cI21460)	3.62e-52 (cI11967)	4.82e-51 (smart00044)	Insert between M1 and M2 of cyclase-1 module
Aast	Aphanomyces astaci	APO3	oomycetes	ETV89303.1	H257_00634	0e+00 (cd02073)	4.50e-52 (pfam00211)	2.48e-51 (smart00044)	Splice variant ETV89302.1 is 17 aa longer
Aast1	Aphanomyces astaci	APO3	oomycetes	ETV89324.1	H257_00650	0.00e+00 (cd02073)	6.47e-47 (cd07302)	5.71e-50 (smart00044)	Has A within DKGTG motif and missing small-conserved region within A-domain near cTM3
Aste	Aphanomyces stellatus	CBS578.67	oomycetes	KAF0683553.1	As57867_024334	0.00e+00 (cd02073)	7.85e-52 (pfam00211)	2.37e-54 (smart00044)	15 amino-acid insert in cTM2 and 20 amino acid insert in M6

Abbr	Species	Strain	Taxon	Protein ID	Gene ID (or contig)	Flippase	Cyclase 1	Cyclase 2	Comment
Aste1	Aphanomyces stellatus	CBS578.67	oomycetes	KAF0683566.1	As57867_024347	0.00e+00 (cd02073)	2.91e-47 (cd07302)	9.34e-49 (smart00044)	Has 17 amino-acid insert in the AxxGxGxxGxEGxQA motif. Nearly identical to Aste. Has extra A in DKTGT motif.
Aste2	Aphanomyces stellatus	CBS578.67	oomycetes	KAF0687277.1	As57867_020897	0e+00 (cd02073)	1.22e-41 (pfam00211)	2.21e-53 (smart00044)	Has 17 amino-acid insert in the AxxGxGxxGxEGxQA motif. Nearly identical to Aste. Region around M8-M9 adversely affects alignment
Aeut	Aphanomyces euteiches	ATCC 201684	oomycetes	KAF0738831.1	Ae201684_005442	0e+00 (cd02073)	1.89e-50 (pfam00211)	5.49e-51 (smart00044)	
	Aphanomyces cochlioides	103-1	oomycetes	KAG9416436.1	AC1031_000839	0e+00 (TIGR01652)	3.31e-38 (pfam00211)	missing	Missing cyclase-2 module
NspC	Nothophytophthora sp.	Chile5	oomycetes	RLN37488.1	BBJ28_00010401	0e+00 (cI21460)	2.72e-55 (pfam00211)	1.44e-45 (cd07302)	
Pram	Phytophthora ramorum		oomycetes	KAL4101936.1		0e+00 (cI21460)	2.13e-55 (pfam00211)	3.11e-32 (cI11967)	
Prub	Phytophthora rubi		oomycetes	KAE9037738.1		0e+00 (cI21460)	2.31e-55 (pfam00211)	4.09e-44 (smart00044)	
Pfra	Phytophthora fragariae		oomycetes	KAE9331399.1	,	0e+00 (cI21460)	3.74e-55 (pfam00211)	4.09e-44 (smart00044)	
Pnic	Phytophthora nicotianae		oomycetes	KUF82984.1		0e+00 (cI21460)	6.80e-55 (pfam00211)	1.90e-32 (cI11967)	
Pcac	Phytophthora cactorum	NZFS 3830	oomycetes	KAG6952375.1	JG687_00013078	0e+00 (TIGR01652)	3.71e-54 (COG2114)	1.96e-32 (smart00044)	Missing region of A-domain. Very long C-terminus
Plil	Phytophthora lilii		oomycetes	GMF34005.1		0e+00 (cd02073)	7.25e-56 (pfam00211)	3.03e-43 (smart00044)	
Pinf	Phytophthora infestans	1306	oomycetes	KAI9983100.1		0e+00 (cd02073)	5.20e-54 (pfam00211)	4.37e-32 (smart00044)	
Pmeg	Phytophthora megakarya	zdho120	oomycetes	OWZ17349.1		0e+00 (cd02073)	1.84e-55 (pfam00211)	2.63e-32 (smart00044)	
Ppse	Phytophthora pseudosyringae	SCRP734	oomycetes	KAG7390726.1		0e+00 (cd02073)	2.36e-54 (pfam00211)	6.45e-32 (smart00044)	
Pole	Phytophthora oleae	VK10A	oomycetes	KAL3669255.1		0e+00 (cd02073)	1.41e-55 (pfam00211)	5.95e-44 (smart00044)	N-terminus distinct from other Phytophora
Pcin	Phytophthora cinnamomi	VT-H3	oomycetes	KAJ8561897.1	ON010_g7781	0e+00 (TIGR01652)	1.93e-55 (pfam00211)	3.40e-45 (smart00044)	Missing region of A-domain.

Abbr	Species	Strain	Taxon	Protein ID	Gene ID (or contig)	Flippase	Cyclase 1	Cyclase 2	Comment
Psoj	Phytophthora sojae	P6497	oomycetes	XP_009537104.1	PHYSODRAFT_529491	0e+00 (cd02073)	7.42e-55 (pfam00211)	1.39e-43 (smart00044)	
Pker	Phytophthora kernoviae	Chile6	oomycetes	RLN61526.1	BBP00_00005346	0e+00 (cd02073)	6.24e-54 (pfam00211)	1.12e-33 (smart00044)	Missing large portion of flippase module and very long C-terminus
Pins	Pythium insidiosum	Pi050C3	oomycetes	KAJ0411864.1	ATCC90586_003017	0e+00 (cd02073)	2.97e-56 (pfam00211)	1.55e-36 (smart00044)	Missing a conserved portion of the N-domain
Lgig	Lagenidium giganteum	ARSEF 373	oomycetes	DAZ98689.1		0e+00 (TIGR01652)	9.54e-55 (pfam00211)	3.28e-37 (smart00044)	Missing some of N-terminus, moderately long C-terminus
Phal	Plasmopara halstedii		oomycetes	XP_024579800.1		0e+00 (TIGR01652)	6.03e-52 (pfam00211)	1.14e-33 (smart00044)	Missing DKTGT motif
Spar	Saprolegnia parasitica	CBS223.65	oomycetes	XP_012195916.1	SPRG_01963	1.40e-176 (TIGR01652)	1.10e-48 (pfam00211)	2.02e-50 (smart00044)	Missing N-terminus.
Sdic	Saprolegnia diclina	VS20	oomycetes	XP_008612444.1		0e+00 (TIGR01652)	2.66e-49 (pfam00211)	1.36e-51 (smart00044)	
Ahyp	Achlya hypogyna	ATCC 48635	oomycetes	OQS01643.1		0e+00 (cd02073)	5.55e-50 (pfam00211)	1.04e-51 (smart00044)	
Tcla	Thraustotheca clavata	ATCC 34112	oomycetes	OQS00002.1		0e+00 (cd02073)	1.07e-50 (pfam00211)	3.76e-53 (smart00044)	Missing conserved region of A-domain,
Poli	Pythium oligandrum	ATCC 38472_TT	oomycetes	TMW62031.1	Poli38472_009524	0e+00 (TIGR01652)	3.61e-55 (pfam00211)	8.81e-34 (smart00044)	Moderately long C-terminus
Gpol	Globisporangium polare	14-1	oomycetes	GAB9466777.1	Gpo141_00004142	0e+00 (TIGR01652)	4.00e-55 (cd07302)	3.87e-35 (smart00044)	Moderately long C-terminus
Pdes	Peronospora destructor	pd1	oomycetes	CAI5738239.1	PDE001_LOCUS6850	0e+00 (cd02073)	1.36e-54 (pfam00211)	2.08e-33 (smart00044)	
Pfar	Peronospora farinosa	pf2	oomycetes	CAI5741095.1	PFR002_LOCUS9470	0e+00 (cd02073)	5.02e-55 (pfam00211)	9.65e-35 (smart00044)	
	Sphaerotrichia firma	ET2F	stramenopile (brown alga)	CAN0238124.1	PHAE_SFIRF_6608.22743.1	4.73e-94 (cd02073)	9.26e-55 (pfam00211)	n.a.	Likely due to aberrant contig assembly and not a valid P4GC gene
Croe	Cafeteria roenbergensis	BVI	stramenopile (bicosoecid)	KAA0157557.1	FNF29_00133	9.64e-132 (cd02073)	4.03e-51 (pfam00211)	2.88e-28 (pfam00211)	
Cbur	Cafeteria burkhardae	ATCC 50561	stramenopile (bicosoecid)	n.a.	(TR13138_c0_g1_i1)	2.08e-117 (cd02073)	3.27e-48 (pfam00211)	4.32e-26 (pfam00211)	Missing some of N-terminus

Abbr	Species	Strain	Taxon	Protein ID	Gene ID (or contig)	Flippase	Cyclase 1	Cyclase 2	Comment
Cpse	Caecitellus pseudoparvulus		stramenopile (bicosoecid)	n.a.	(CAE72_contig_139_np1212)	6.99e-97 (cd02073)	1.28e-56 (pfam00211)	3.17e-32 (smart00044)	Missing some of N-terminus
	Halocafeteria seosinensis	EHF34	stramenopile (bicosoecid)	None detected					No combined P4GC gene detected, just separate flippase and cyclase genes.

The abbreviations (Abbr) consist of the first letter of the genus and the first three letters of species. A and B refer to the alpha and beta types respectively in the haemosporidans. Other paralogues are denoted with numbers. Lack of an abbreviation indicates that the sequence was compromised and not used for alignments or homology modeling. Protein ID for genes generated from partially annotated genomic sequence is designated as n.a. (not applicable) and the contig(s) are indicated in the Gene ID column in parentheses. The E-values (and CDD accession numbers) for the flippase, cyclase-1, and cyclase-2 modules are shown. The value of zero indicates a value less than 5e-324. Descriptions of the flippase conserved domains: cl21460, haloacid dehalogenase-like hydrolases superfamily (includes cd02073 and tigr01652); cd02073, aminophospholipid translocases similar to *Saccharomyces cerevisiae* Dnf1-3p, Drs2p, and human ATP8A2, -10D, -11B, -11C; and tigr01652, phospholipid-translocating P-type ATPase (flippase). Descriptions of the cyclase conserved domains: cl11967, nucleotidyl_cyc_III superfamily (includes pfam00211 and smart00044); pfam00211, adenylate and guanylate cyclase catalytic domain; smart00044, adenylyl- / guanylyl cyclase catalytic domain; cd07302, cyclase homology domain; and COG2114, adenylate cyclase class 3. [1]From Günay-Esiyok Ö, Gupta N, Chimeras of P4-ATPase and guanylate cyclase in pathogenic protists, Trends in Parasitology (2020) 36:382-392.
