# Supplementary material for: Origins and Molecular Features of a Chimeric Type-P4A ATPase—Guanylate Cyclase from Stramenopiles and Alveolates
Source: Int J Mol Sci. 2026 Jul 28;27(15):6734. doi: 10.3390/ijms27156734 (PMC13466520; doi:10.3390/ijms27156734)
Supplement: Supplementary file 1 [file ijms-27-06734-s001.zip › Supplemental Table S2.pdf]

Supplemental Table S2a. Highest scoring templates identified by Phyre®

		Flippase Module					Cyclase Module		
Group	Query	1st	2nd	3rd	4th	5th	1st	2nd	3rd
Oomycetes	Pram	6roh	7drx	6k7k	7py4	8ox9	8buz	-	-
	NspC	6k7k	7drx	9dzu	6roh	7py4	8sl4A	8sl4B	-
	Ainv	6k7k	7drx	8ox9	7py4	7vsh	8buz	8sl4A	8sl4B
Ciliates	Pson	8ox9	7py4	6roh	7drx	6k7k	6jt0A	-	-
	Tpyr	7drx	8ox9	6roh	6k7k	7py4	6jt0A*	-	-
	Bsto	6k7k	6roh	8ox9	7py4	9dzu	8buz	-	-
	Imul	7py4	7drx	7vsh	8ox9	6clr	8buz	8sl4A	8sl4B
	Scoe	6roh	6k7k	8ox9	7py4	9dzu	8buz	-	-
Dinoflagellates	Smic	6k7k	8ox9	7py4	6roh	7drx	8sl4A	-	-
	Spil	7py4	8ox9	6k7k	6roh	7drx	8sl4A	-	-
	Cgor	8ox9	7py4	6roh	6k7k	9dzu	8sl4A	-	-
	Pgla	6roh	6k7k	7drx	8ox9	7py4	8sl4A	-	-
	Dtre	7py4	8ox9	6roh	9dzu	6clr	8buz	-	-
	Evor	7py4	8ox9	6k7k	6roh	9dzu	8sl4A	-	-
Perkinsids	Pmar	7drx	6k7k	6roh	7py4	8ox9	8buz	-	-
	Pche	6k7k	6roh	7drx	8ox9	7py4	8sl4A	-	-
Colpodellids	Vbra	6k7k	6rohh	8ox9	9dzu	7drx	8sl4A	-	-
Gregarines	Pgig	8ox9	7py4	6k7k	7drx	7vsh	8buz	8sl4A	8sl4B
	Gnip	9dzu	7py4	8ox9	7vsh	7ky7	8buz	8sl4A*	8sl4B*
Coccidia	Tgon	7py4	8ox9	7drx	6k7k	7vsh	8buz	8sl4A	8sl4B
	Enec	7drx	6k7k	7py4	8ox9	7vsh	8sl4A	8sl4B	8buz
	Ccay	7drx	6lcr	7vsh	6roh	8ox9	8bv5	8sl4B*	-
	Scal	9dzu	7drx*	7ky7*	6clr*	7op3*	6r4p	-	-
Piroplasmids	Bdiv	6roh	7drx	9dzu	7vsh	6clr	8buz	8sl4A	8sl4B
	Bovi	6roh	7drx	6lcr	8ox9	7py4	8buz	8sl4A	8sl4B
	Tpar	7vsh	6roh	7drx	6k7k	9dzu	8sl4A	8sl4B	8buz
Haemosporidans-α	HtarA	7drx	7py4	8ox9	6k7k	7vsh	8buz	8sl4A	8sl4B
	PrelA	7drx	6roh	7py4	7vsh	7py4	8buz	8sl4A	8sl4B
	PfalA	7drx	6k7k	7vsh	7py4	8ox9	8buz	8sl4A	8sl4B
	PgonA	6roh	7drx	7py4	7vsh	9dzu	8buz	8sl4A	8sl4B
	PvivA	6roh	7drx	6k7k	7vsh	9dzu	8buz	8sl4A	8sl4B
	PmalA	7drx	6k7k	7py4	7vsh	8ox9	8buz	8sl4A	8sl4B
	PovCA	6roh	7drx	6k7k	7py4	9dzu	8buz	8sl4A	8sl4B
	HsppA	7drx	8ox9	7py4	6k7k	7vsh	8buz	8sl4A	8sl4B
	PberA	6roh	7drx	6k7k	7py4	8ox9	8buz	8sl4A	8sl4B
Haemosporidans-β	PrelB	6roh	7py4	8ox9	7vsh	7drx	8buz	8sl4A	8sl4B
	PfalB	6roh	7drx	7vsh	8ox9	7py4	8sl4A	8sl4B	8buz
	PgonB	6roh	7py4	7drx	8ox9	6k7k	8sl4A	8sl4B	8buz
	PvivB	7drx	8ox9	6k7k	6roh	7py4	8buz	8sl4A	8sl4B

	PmalB	7py4	6roh	7drx	8ox9	6k7k	8buz	8sl4A	8sl4B
	PovCB	7drx	7py4	8ox9	6roh	6k7k	8buz	8sl4A	8sl4B
	HsppB	6k7k	6lcr	6ln5	3tlm	6ln8	6jt0A	c1yk	6jt0B
	PvinB	6roh	7py4	8ox9	6k7k	7vsh	8buz	-	-

Shown are the PDB accession numbers of the top five templates that were identified for the flippases module and the top three templates that were identified for the cyclase module with the indicated P4GC sequence. Species abbreviations are described in Supplemental Table S1. An * denotes that only a portion of the module was modeled and a dash (-) indicates that less than three templates were identified for the cyclase module. The A and B notations on the cyclase templates denote proteins that have no c1b region and are true heterodimers (i.e., A subunit or B subunit) instead of two cyclase modules on a single polypeptide chain that form a pseudo-heterodimer.

Supplemental Table S2b. Template descriptions identified by Phyre®

Acc. No.	Species	Gene	Description
7drx	*Saccharomyces cerevisiae*	Dnf1	Type-P4A ATPase
6roh	*S. cerevisiae*	Drs2	Type-P4A ATPase
7py4	*Homo sapiens*	ATP8B1	Orthologue of Dnf1 and Dnf2
8ox9	*H. sapiens*	ATP8B1	Orthologue of Dnf1 and Dnf2
6k7k	*H. sapiens*	ATP8B1	Orthologue of Dnf1 and Dnf2
9dzu	*Cryptococcus neoformans*	Apt1	Orthologue of Drs2
7vsh	*H. sapiens*	ATP11C	Related to Dnf1, Dnf2, and Drs2
6lcr	*Thermochaetoides thermophila*	Dnf1	Type-P4A ATPase
7ky7	*S. cerevisiae*	Dnf2	Type-P4A ATPase
8buz	*Bos taurus*	AC8	Class IIIa calmodulin regulated adenylate cyclase
8sl4	*H. sapiens*	AC5	Dimeric class IIIa adenylate cyclase
6r4p	*B. taurus*	AC9	Soluble class IIIb adenylate cyclase
8bv5	*B. taurus*	AC8	Catalytic domain of class III adenylate cyclase
6jt0	*H. sapiens*	GC	Soluble guanylate cyclase
